# Supplementary material for: Vitamin D status among adolescents in Kuwait: a cross-sectional study
Source: BMJ Open. 2018 Aug 1;8(7):e021401. doi: 10.1136/bmjopen-2017-021401 (PMC6074625; doi:10.1136/bmjopen-2017-021401)
Supplement: Supplementary file 1 [file bmjopen-2017-021401supp001.pdf]

Supplementary Table (1). Factors associated with vitamin D deficiency among adolescents in univariate analysis.

Characteristics	Total	Prevalence of vitamin D deficiency n=1150	(%)	Prevalence Ratio [95%CI]		p
**Gender**						
Male	694	488	(70.32)	1	[Ref.]	<0.001
Female	722	662	(91.69)	1.30	[1.26- 1.33]	
**Age (year)**						
<12	527	409	(77.61)	1	[Ref.]	0.024
12-	439	369	(84.05)	1.07	[1.01- 1.12]	
≥13	450	372	(82.67)	1.06	[1.00- 1.10]	
**Nationality**						
Kuwaiti	1081	900	(83.26)	1	[Ref.]	<0.001
Non-Kuwait	335	250	(74.63)	0.90	[0.82-0.96]	
**Governorate**						
Capital	156	127	(81.41)	1	[Ref.]	0.003
Hawally	246	188	(76.42)	0.94	[0.82-1.03]	
Farawanya	236	183	(77.54)	0.95	[0.83-1.04]	
Jahra	239	214	(89.54)	1.10	[1.02-1.15]	
Mubarak al-Kabeer	148	124	(83.78)	1.03	[0.91-1.10]	
Ahmadi	371	296	(79.78)	0.98	[0.88-1.06]	
**Father's Education**						
Primary/Intermediate/no formal education	236	205	(86.86)	1	[Ref.]	<0.001
Secondary (high school)	344	297	(86.34)	0.99	[0.87-1.08]	
Diploma	261	220	(84.29)	0.95	[0.82-1.06]	
University & above	542	399	(73.62)	0.78	[0.65-0.90]	
**Mother's Education**						
Primary/Intermediate/no formal education	183	153	(83.61)	1	[Ref.]	<0.001
Secondary (high school)	304	263	(86.51)	1.04	[0.94-1.12]	
Diploma	304	259	(85.20)	1.02	[0.91-1.10]	
University & above	605	457	(75.54)	0.88	[0.76-0.99]	
**Total number of brother/sisters**						
Zero-two	298	223	(74.83)	1	[Ref.]	0.002
Three-four	538	434	(80.67)	1.05	[1.00-1.10]	
Five or more	553	469	(84.81)	1.10	[1.04-1.13]	
**Passive smoking at household**						
No	901	704	(78.14)	1	[Ref.]	<0.001
Yes	489	426	(87.12)	1.11	[1.06-1.15]	

**Times per week consumed breakfast not prepared at home**							
Zero	624	484	(77.56)	1	[Ref.]	0.019
One-two times	569	478	(84.01)	1.08	[1.02-1.12]	
Three-four	117	98	(83.76)	1.07	[0.97-1.14]	
Five or more	78	67	(85.90)	1.10	[1.00-1.17]	
**Times per week consumed dinner not prepared at home**						
Zero	157	114	(72.61)	1	[Ref.]	0.018
One-two times	878	719	(81.89)	1.09	[1.03-1.14]	
Three-four	257	211	(82.10)	1.09	[1.01-1.15]	
Five or more	86	75	(87.21)	1.14	[1.04-1.19]	
**Consumption of sugary drinks per week**						
None	171	123	(71.93)	1	[Ref.]	0.002
Once-Three times	772	633	(81.99)	1.09	[1.04-1.14]	
Four-Six	171	135	(78.95)	1.07	[0.98-1.13]	
Seven/more	299	256	(85.62)	1.12	[1.07-1.17]	
**Currently taking supplements**						
No	1,256	1,045	(83.20)	1	[Ref.]	<0.001
Yes	158	103	(65.19)	0.78	[0.68-0.88]	
**Number of times walking to school per week**						
None	1,158	982	(84.80)	1	[Ref.]	<0.001
1-8 times	155	99	(63.87)	0.74	[0.63-0.83]	
Every day	103	69	(66.99)	0.77	[0.65-0.89]	
**Time walking to school**						
None	1,158	982	(84.80)	1	[Ref.]	<0.001
≤5 minutes	80	50	(62.50)	0.71	[0.56-0.84]	
6- 10 minutes	76	53	(69.74)	0.80	[0.65-0.93]	
11-15	56	38	(67.86)	0.77	[0.60-0.92]	
16 minutes or more	45	26	(57.78)	0.65	[0.46-0.82]	
**Time spent on physical activity per week**						
Low (lower tertile)	469	396	(84.43)	1	[Ref.]	0.005
Medium (middle tertile)	473	391	(82.66)	0.97	[0.89-1.04]	
High (higher tertile)	474	363	(76.58)	0.89	[0.80-0.96]	
**Body Mass Index Categories**[1]						
Normal weight	601	465	(77.37)	1	[Ref.]	0.001
Overweight	320	270	(84.38)	1.07	[1.02-1.11]	

Obese	471	400 (84.93)	1.08	[1.03-1.11]	
Under weight	24	15 (62.50)	0.84	[0.59-1.02]	
**Time outside per day 10:00 am- 4:00 pm week days**					
Less than 30 minutes	657	563 (85.69)	1	[Ref.]	<0.001
31 minutes to 1 hour	450	342 (76.00)	0.87	[0.80-0.94]	
More than 1 hour to 2 hours	222	178 (80.18)	0.93	[0.84-1.00]	
More than 2 hours	87	67 (77.01)	0.88	[0.74-0.99]	
**Time outside per day 10:00 am- 4:00 pm week ends**					
Less than 30 minutes	494	425 (86.03)	1	[Ref.]	0.003
31 minutes to 1 hour	312	257 (82.37)	0.94	[0.85-1.02]	
More than 1 hour to 2 hours	255	198 (77.65)	0.87	[0.76-0.96]	
More than 2 hours to 3 hours	185	142 (76.76)	0.85	[0.74-0.96]	
More than 3 hours	169	127 (75.15)	0.83	[0.71-0.94]	
**Wearing sunscreen**					
Never	1,100	874 (79.45)	1	[Ref.]	0.013
Rarely	77	65 (84.42)	1.06	[0.94-1.13]	
Sometimes	160	140 (87.50)	1.09	[1.02-1.14]	
Often/always	79	71 (89.87)	1.12	[1.02-1.18]	
**Staying in shade or under an umbrella**					
Never	493	396 (80.32)	1	[Ref.]	0.04
Rarely	97	70 (72.16)	0.90	[0.78-1.01]	
Sometimes	363	301 (82.92)	1.03	[0.96-1.08]	
Often	205	162 (79.02)	0.98	[0.90-1.05]	
Always	257	220 (85.60)	1.06	[0.99-1.11]	

%s are row percentages. [1]according to WHO growth charts.
