# Supplementary material for: Vitamin D status among adolescents in Kuwait: a cross-sectional study
Source: BMJ Open. 2018 Aug 1;8(7):e021401. doi: 10.1136/bmjopen-2017-021401 (PMC6074625; doi:10.1136/bmjopen-2017-021401)
Supplement: Author's manuscript [file bmjopen-2017-021401.draft_revisions.pdf]

BMJ Open

BMJ Open is committed to open peer review. As part of this commitment we make the peer review history of every article we publish publicly available.

When an article is published we post the peer reviewers' comments and the authors' responses online. We also post the versions of the paper that were used during peer review. These are the versions that the peer review comments apply to.

The versions of the paper that follow are the versions that were submitted during the peer review process. They are not the versions of record or the final published versions. They should not be cited or distributed as the published version of this manuscript.

BMJ Open is an open access journal and the full, final, typeset and author-corrected version of record of the manuscript is available on our site with no access controls, subscription charges or pay-per-view fees (http://bmjopen.bmj.com).

If you have any questions on BMJ Open's open peer review process please email info.bmjopen@bmj.com

Vitamin D Status among Adolescents in Kuwait: A Cross-sectional Study.

Journal:	*BMJ Open*
Manuscript ID	bmjopen-2017-021401
Article Type:	Research
Date Submitted by the Author:	28-Dec-2017
Complete List of Authors:	Al-Taiar, Abdullah; Faculty of Medicine, Kuwait University, Department of Community Medicine and Behavioural Sciences<br>Rahman , Abdur ; College of Life Sciences, Kuwait University, Department of Food Science and Nutrition<br>Al-Sabah, Reem; Faculty of Medicine, Kuwait University, Department of Community Medicine and Behavioural Sciences<br>Shaban, Lemia ; College of Life Sciences, Kuwait University, Department of Food Science and Nutrition<br>Al-Harbi, Anwar ; Kuwait Institute for Scientific Research , Department of Science and Nutrition
Keywords:	PUBLIC HEALTH, Vitamin D, Adolescents, school children, Kuwait

SCHOLARONE™
Manuscripts

For peer review only

Vitamin D Status among Adolescents in Kuwait: A Cross-sectional Study.

Abdullah Al-Taiar[1], Abdur Rahman[2], Reem Al-Sabah[1], Lemia Shaban[2], Anwar Al-Harbi[3]

[1] Department of Community Medicine and Behavioural Sciences

Faculty of Medicine, Kuwait University

Box: 24923 Safat-13110 Kuwait.

[2] Department of Food Science and Nutrition

College of Life Sciences, Kuwait University.

Box 5969, Safat-13060, Kuwait

[3] Kuwait Institute for Scientific Research

Department of Science and Nutrition

Kuwait

Box: 24885 Safat-13109, Kuwait

<u>Before publication</u> corresponding author:

Dr Abdullah Al-Taiar

Associate Professor of Epidemiology

Dept. Community Medicine and Behavioural Sciences

Faculty of Medicine, Kuwait University

Box: 24923 Safat-13110 Kuwait.

Tel: +965-24986553

Fax: +965-5338948

E-mail: altaiar@hsc.edu.kw

For peer review only

For peer review only - http://bmjopen.bmj.com/site/about/guidelines.xhtml

After publication corresponding author:

Abdur Rahman

Associate Professor

Department of Food Science and Nutrition

College of Life Sciences, Kuwait University

Box: 5969 Safat-13060 Kuwait.

Tel: +965-249633055

Fax: +965-22513929

E-mail: abdurrahman.ahmad@ku.edu.kw

Word count: 3389

For peer review only - http://bmjopen.bmj.com/site/about/guidelines.xhtml

Abstract

Objectives

In Kuwait as in many Arab states in the gulf region, there are limited data on the prevalence of vitamin D deficiency among healthy adolescents. This study aimed to estimate the prevalence of vitamin D deficiency in a nationally representative sample of adolescents and investigate factors associated with vitamin D status.

Methods

Cross-sectional study was conducted on 1416 adolescents who were randomly selected from middle schools in all governorates of Kuwait. Data were collected from parents and adolescents; while vitamin D was measured using liquid chromatography-tandem mass spectrometry (LC-MS/MS). Logistic regression was used to investigate the independent factors associated with vitamin D status.

Results

The Prevalence of vitamin D deficiency was 1,150 (81.21%, 95%CI: 71.61-90.81) while severe deficiency was 559 (39.48%). Only 51 (3.60%) were vitamin D sufficient. Prevalence was significantly higher among females compared to males (91.69% vs. 70.32%; $p<0.001$). There was a significant inverse correlation between vitamin D and PTH (Spearman correlation=-0.35; $p<0.001$). In the final model, gender, age, governorate, parental education, body mass index, vitamin D supplement and number of times walking to school per week were all significantly related to vitamin D deficiency.

Conclusion

High prevalence of vitamin D deficiency was noted among adolescents in Kuwait despite the abundant sunshine which may reflect strong sun avoidance behavior. Adequate outdoor daytime activities should be encouraged especially for females. We call for locally tailored guidelines for supplement in which females should have a higher amount of vitamin D supplement compared to males.

Keywords: Vitamin D, adolescents, Kuwait, Middle East, school children

For peer review only

Strengths and limitations of this study

- This is the first study that aimed to estimate the prevalence of vitamin D on a large nationally representative sample of adolescents in Kuwait.
- We reported high prevalence of vitamin D particularly among females despite the abundant sunshine, and thus, skepticism about this issue should be cleared.
- We measured vitamin D using the recommended laboratory method and gathered data from both parents and adolescents.
- We did not measure the skin color, which can be an important determinant for vitamin D formulation in skin.

For peer review only

For peer review only - http://bmjopen.bmj.com/site/about/guidelines.xhtml

Introduction

An optimal vitamin D status is extremely important during adolescence for proper growth and bone mineral accrual [1]. In addition to its critical role in bone health, several studies have linked vitamin D deficiency with various disease conditions during adolescence or adulthood such as asthma and allergies [2], type 2 diabetes [3], depression[4], cancer and even all-cause mortality [5]. However, the impact of vitamin D supplement on the risk of these diseases has not yet been tested in randomized control trials and thus remain under intense debate.

Several studies have evaluated the prevalence of vitamin D deficiency and insufficiency during adolescence reporting high prevalence of vitamin D deficiency worldwide [6, 7]. This was the case even in countries with abundant sunshine, which is the main factor for endogenous vitamin D synthesis. For example, in India the prevalence of vitamin D deficiency among adolescents was reported to be 85-98% [8, 9] while in Saudi Arabia it was found to be around 96% [10].

In Arab states in the Gulf region or the broader Middle East, there is paucity of data on vitamin D status in pediatric and adolescents population. The few studies that showed a high prevalence of vitamin D deficiency suffered from major methodological weaknesses. It is also known that factors which influence vitamin D status such as avoiding sun exposure, skin pigmentation, high body mass index (BMI) and dietary factors, particularly the lack of vitamin D supplementation and intake of vitamin D rich food [11] are all on rise in Arab states in the Gulf region. The aim of the present study was therefore to assess vitamin D status among adolescents living in Kuwait, a country with a plenty of sun shine.

For peer review only

For peer review only - http://bmjopen.bmj.com/site/about/guidelines.xhtml

Methods

Kuwait is a small country at latitude (29.3759° N) with a population of 4.2 million. Approximately 25% of population is under the age of 19-year. The study population was students in the age between 11 and 16 years in the middle public schools. School based cross-sectional study was conducted on students from 12 middle public schools that were selected using stratified multistage cluster random sampling with probability proportional to size from all governorates of Kuwait. The sample allocation in each governorate was based on the relative size of that governorate as judged by the total number of students in the governorate. The study was funded by Kuwait University approved by The Ethics Committee at Ministry of Health in Kuwait as well as The Ethics Committee at Health Sciences Centre, Kuwait University.

An informed consent was sent to the parents along with a self-administered questionnaire on parents' level of education and income, type of housing, number of siblings of the index child, passive smoking at household and the number of times per week the index child had a meal prepared outside home during the last three months. After obtaining written informed consent from the parents and verbal consent from the school children, trained dedicated personnel carried out face-to-face interview with the students using structured questionnaire. The questionnaire was carefully developed after extensive review of the literature and was pilot tested on 20 students who were not included in the study. It comprised questions on habitual sun exposure during the last three months, which was assessed by the core questions developed to measure the exposure to sunlight in adolescents as described by Glanz et al. [12]; in addition to smoking habits, physical activity and dietary intake. Data on physical activity were collected using a questionnaire that was developed based on Youth Physical Activity Questionnaire in UK [13] and The Arab teens lifestyle study [14]. The questionnaire was validated among high school students and showed strong correlation with data collected by accelerometers (Spearman correlation 0.92; p<0.001 for total steps count) (not published).

For peer review only - http://bmjopen.bmj.com/site/about/guidelines.xhtml

Data on dietary intake of vitamin D were collected using food frequency questionnaire for calcium and vitamin D intake in adolescents [15], which have been validated in our settings [16]. Food models or serving containers were used to assist in estimating serving size. Measurements of standing height and body weight of the study subjects were assessed using digital weight and height scale in a standardized manner.

Five ml of venous blood was collected in gel-containing tubes (SST II advance, BD vacutainer) from each child by a trained nurse; and the samples were protected from light. On the same day, the samples were centrifuged at 2000 x g for 15 minutes and the serum was transferred to Eppendorf tubes and stored at -80°C until analysis. Serum 25-hydroxyvitamin D (25-OH-D) concentration is the best marker of vitamin D status as it is the major circulating form of vitamin D, reflecting both the amount produced in the skin after sun exposure and the amount consumed in foods [17]. It was measured in College of American Pathologists CAP-accredited laboratory by liquid chromatography-tandem mass spectrometry (LC-MS/MS), which is the recommended method of vitamin D assessment in epidemiological studies [18]. According to the Endocrine Society [19] and the Society for Adolescent Health and Medicine [20], we used the following cut-off of 25-OH-D to define vitamin D status: vitamin D deficiency < 50 nmol/L (20 ng/mL); vitamin D insufficiency 50-75 nmol/L (20-30 ng/mL); vitamin D sufficiency≥ 75 nmol/L (30 ng/mL). Thus, hypovitaminosis D was defined in the presence of 25-OH-D levels < 75.0 nmol/L (30 ng/mL). Furthermore, severe vitamin D deficiency was defined as 25-OH-D levels < 25.0 nmol/L (10 ng/mL) [21]. PTH level ≥ 65.0 ng/L was suggestive of hyperparathyroidism.

Data were double entered into specifically designed database using Epidata Entry. Data analysis was conducted using Stata (StataCorp. 2011. Release 12). BMI was calculated as weight (Kg) divided by height squared (m^2). Weight status was categorized into normal, overweight and obese according to WHO growth charts. Chi-squared test was used to test differences in categorical variables. A modified version of multiple logistic regression that calculates prevalence ratio was used to study the association between each

For peer review only - http://bmjopen.bmj.com/site/about/guidelines.xhtml

presumed risk factor and vitamin D deficiency (25-OH-D < 50 nmol/L). This is because vitamin D deficiency was a common outcome and using log-binomial regression was not possible because the model failed to converge. Variables that were found to be statistically significant at 15% level of significance were considered in multivariate analysis. Variables were divided into groups (e.g. socio-demographic, sun exposure, etc) and were added sequentially to the model. Statistical significance of variables was evaluated by likelihood ratio test which compares the model with and without the variable. Goodness of fit of the final model was evaluated by Hosmer-Lemeshow test. Factors with $p<0.05$ were deemed to be statistically significant. The analysis above was repeated using hypovitaminosis (25-OH-D levels < 75.0 nmol/L) as the binary outcome. We also conducted multiple linear regression analysis using vitamin D level as a continuous outcome (after log-transformation) and reported the results in the text. Because of the complex structure of this survey data, we used survey method, which gives more precise estimates of standard error.

Results

Of 1583 parents approached, 161 refused to participate (children, parents or both). Another 6 samples were not sufficient to conduct blood analysis, thus the analysis below comprised 1416 students. Table (1) shows the socio-demographic characteristics of the study group. The mean (SD) age was 12.48 (0.94) years and 694 (49.01%) were females.

For peer review only

Table (1). Socio-demographic characteristics of 1416 adolescents in public middle schools in Kuwait.

Characteristics		
**Age in years,** Mean (SD) years	12.48	0.94
	**n**	**(%)**
**Gender**		
Male	694	(49.01)
**Nationality**		
Kuwaiti	1,081	(76.34)
Non-Kuwait	335	(23.66)
**Father's Education**[1]		
No formal education	15	(1.08)
Primary/Intermediate	221	(15.98)
Secondary (high school)	344	(24.87)
Diploma	261	(18.87)
University & above	542	(39.19)
**Mother's Education**[2]		
No formal education	31	(2.22)
Primary/Intermediate	152	(10.89)
Secondary (high school)	304	(21.78)
Diploma	304	(21.78)
University & above	605	(43.34)
**Father's Income**[3] (Kuwaiti Dinars)		
Less than 500	91	(6.64)
500 to 1000	304	(22.19)
1001 to 1500	421	(30.73)
1501 to 2000	219	(15.99)
More than 2000	173	(12.63)
Do not wish to tell	162	(11.82)
**Mother's Employment Status**[4]		
Housewife	488	(35.16)
Paid employment	680	(48.99)
Others	220	(15.85)
**Housing**[5]		
Rented flat	510	(36.51)
Rented house	163	(11.67)
Owned flat	59	(4.22)
Owned house	665	(47.60)

[1]Missing for 33 participants; [2] Missing for 20 participants; [3] Missing for 46 participants; [4]Missing for 28 participants; [5]Missing for 19 participants

For peer review only - http://bmjopen.bmj.com/site/about/guidelines.xhtml

The median serum (Interquartile Range, IQR) 25-OH-D was 29.7 (19.2- 44.2) nmol/L. Prevalence of vitamin D deficiency was 1,150 (81.21%, 95%CI: 71.61-90.81) while severe deficiency was 559 (39.48%). Only 51 (3.60%) were vitamin D sufficient. Prevalence was significantly higher among females compared to males (91.69% vs. 70.32%; p<0.001). Also, the median (IQR) of vitamin D was 39.8 (29.4- 52.7) nmol/L and 21.5 (14.7- 30.7) among males and females, respectively (p<0.001). There was no difference in the prevalence of vitamin D deficiency between Kuwaitis and non-Kuwaitis after stratification by gender. There was significant inverse correlation between vitamin D and PTH (Spearman correlation=-0.35; p<0.001). In relation to vitamin D status, elevated PTH (secondary hyperparathyroidism PTH ≥65 ng/L. i.e. 6.89 pmol/L) was detected in 312 (55.81%), 185 (31.30%) and 59 (27.57%) of adolescents with vitamin D severe deficiency, deficiency and insufficiency, respectively. Serum median (IQR) PTH was significantly different dependent on vitamin D status (severe deficiency 7.37 [5.44-10.55] pmol/L; deficiency 5.63[4.13-7.40] pmol/L; insufficiency 5.14[3.87-6.99] pmol/L; sufficiency 4.31[3.75- 5.73] pmol/L; p<0.001). The actual relationship between 25-OH-D and PTH levels is shown in figure (1).

Factors that showed significant association with vitamin D deficiency (25-OH-D < 50 nmol/L) in univariate analysis were Gender, parental education, number of times per week the participants consumed breakfast and dinner prepared outside home, taking supplements, walking to school (instead of using school bus or car) in addition to BMI were all significant predictors in univariate analysis (Supplementary Table). Similarly, time spent outdoor per day (between 10:00 am and 4:00 pm) during weekdays and weekends in addition to wearing sunscreen and staying in shade or under an umbrella were all significant predictors in univariate analysis.

For peer review only - http://bmjopen.bmj.com/site/about/guidelines.xhtml

Table (2): Factors associated with vitamin D deficiency in multivariate analysis, 1416 middle school students.

Characteristics	Prevalence n (%)	Prevalence Ratio [95%CI]	p
**Gender**			
**Male**	488 (70.32)	1 [Ref.]	<0.001
**Female**	662 (91.69)	1.20 [1.17- 1.21]	
**Age (year)**			
**<12**	409 (77.61)	1 [Ref.]	0.003
**12-**	369 (84.05)	1.10 [1.04- 1.14]	
**≥13**	372 (82.67)	1.07 [1.01- 1.12]	
**Governorate[1]**			
**Capital**	127 (81.41)	1 [Ref.]	<0.001
**Hawally**	188 (76.42)	0.88 [0.73-1.01]	
**Farawanya**	183 (77.54)	0.85 [0.68-0.99]	
**Jahra**	214 (89.54)	1.10 [0.98-1.17]	
**Mubarak al-Kabeer**	124 (83.78)	1.06 [0.93-1.14]	
**Ahmadi**	296 (79.78)	1.06 [0.95-1.13]	
**Father's Education[2]**			
**Primary/Intermediate/no formal education**	205 (86.86)	1 [Ref.]	<0.009
**Secondary (high school)**	297 (86.34)	1.04 [0.92-1.12]	
**Diploma**	220 (84.29)	1.01 [0.88-1.10]	
**University & above**	399 (73.62)	0.89 [0.76-1.01]	
**Passive smoking in household[3]**			
**No**	704 (78.14)	1 [Ref.]	<0.006
**Yes**	426 (87.12)	1.08 [1.02-1.13]	
**Currently taking supplements[4]**			
**No**	1,045 (83.20)	1 [Ref.]	<0.001
**Yes**	103 (65.19)	0.63 [0.49-0.76]	
**Consumption of sugary drinks per week**			
**None**	123 (71.93)	1 [Ref.]	0.002
**Once-Three times**	633 (81.99)	1.11 [1.05-1.16]	
**Four-Six times**	135 (78.95)	1.07 [0.96-1.14]	
**Seven/more times**	256 (85.62)	1.15 [1.08-1.19]	
**Number of times walking to school per week[5]**			
**None**	982 (84.80)	1 [Ref.]	<0.001
**1-8 times**	99 (63.87)	0.81 [0.68-0.93]	
**Every day**	69 (66.99)	0.77 [0.61-0.92]	

For peer review only - http://bmjopen.bmj.com/site/about/guidelines.xhtml

**Body Mass Index Categories[6]**					
**Normal weight**	465	(77.37)	1	[Ref.]	0.001
**Overweight**	270	(84.38)	1.08	[1.01-1.13]	
**Obese**	400	(84.93)	1.12	[1.07-1.16]	
**Underweight**	15	(62.50)	0.93	[0.67-1.10]	

[1]Missing for 20 participants; [2] Missing for 33 participants; [3]Missing for 26 participants; [4] Missing for 2 participants; [5] Missing for 26 participants;[6] according to WHO growth charts.

Table (2) shows the independent predictors for vitamin D deficiency in multivariate analysis. In the final model, gender, age, governorate, parental education, vitamin D supplement, number of times walking to school per week were all significantly related to vitamin D deficiency. Dietary intake data were available only for a few hindered, and of all food items, only eggs were significantly related to vitamin D deficiency in multivariate model. We repeated the analysis above using hypovitaminosis (sufficient Vs. insufficient/deficient) as a binary outcome. The same factors were found to be significantly related to hypovitaminosis, although mother's education (instead of father's education) was found to be significant in this analysis. We also used linear regression to identify the factors associated with vitamin D level as a continuous variable. Factors that showed significant association with vitamin D level in multivariate analysis were age, gender, governorate, passive smoking at household, number of times of having breakfast meal outside home in the last three months, time spent outdoor during weekdays, preference to stay in shade or under the umbrella, and BMI categories. With respect to dietary factors, milk and cheese, fast food hamburger and tuna fish were significantly associated with vitamin D level in univariate linear regression. When these food items were introduced to the final model, only milk and tuna fish were significantly associated with vitamin D level. It is worth noting that in this analysis the best model was able to explain only 45% of the variability in the vitamin D level.

For peer review only - http://bmjopen.bmj.com/site/about/guidelines.xhtml

Discussion

Although several reports have described Vitamin D deficiency among adults in the Arab states in the Gulf region, there are limited data on the prevalence of vitamin D deficiency among healthy adolescents. Our study used a nationally representative sample to examine a large number of healthy adolescents, becoming to our knowledge the only study focusing on this group in Kuwait. We used the recommended method to measure vitamin D level and demonstrated that vitamin D deficiency is too common despite the abundant sunshine and that the secondary hyperparathyroidism due to vitamin D deficiency is also common.

In our setting, approximately, 81% and 15% of adolescents are vitamin D deficient or insufficient, respectively. This is higher than that reported among adolescents in European countries [22-24], southeastern United States [25, 26] or New Zealand [27]. Our result is more akin to that reported from Saudi Arabia (95.6%) [10], India (85-98%) [8, 9], or Korea where only 2.3% of adolescents were found to be vitamin D sufficient [28]. Our findings are also in accordance with the high prevalence reported consistently among adult population in Kuwait [29] or other countries in the Gulf region such as Saudi Arabia [30], Bahrain [31] and Qatar [32].

There has been a lot of skepticism of vitamin D deficiency in the Arab states in the Gulf region due the abundant sunshine. Informal discussions, even among scientific community, in the region show strong denial attributing the problem to wrong measurements or any other reasons. Nevertheless, the studies consistently reported high prevalence of vitamin D deficiency including those studies which used different measurement methods and also in different age groups. Factors that may hinder cutaneous synthesis of vitamin D despite sunshine may include dust storms (occur almost in one third of the days in Kuwait [33]), indoor lifestyle, head covering and other cultural practices in addition to the use of sunscreen. Given the fact that at our latitude, sun exposure is effective in synthesizing vitamin D throughout the entire year, the high

For peer review only - http://bmjopen.bmj.com/site/about/guidelines.xhtml

prevalence of vitamin D deficiency may reflect strong sun avoidance behavior. Only around 6% of the adolescents spent more than two hours outdoor per day between 10:00 am and 4:00 pm during weekdays (data not shown). This can be further supported by the counterintuitive seasonal variation that has been reported in the region, in which the prevalence of vitamin D deficiency is higher in summer compared to winter [34]. Owing to desert climate, avoiding sun exposure would be stronger during summer due to extremely high temperature. We collected blood samples in February, March and April and thus we are unable to describe seasonal variation in vitamin D status. Other factors such as using sunscreen and dust storms cannot also be excluded. Exposure to sunlight contributes up to 90–95% of vitamin D supply, while the number of foods naturally containing a significant quantity of vitamin D is very limited except for some oily fishes that rarely consumed by adolescents worldwide and in our setting [35] (~5% of adolescents in our setting consumed salmon once per week or more). As such, education of the parents on the safe amount of sun exposure in addition to changes in the school environment may produce the highest impact on vitamin D status among adolescents.

Like several studies that investigated vitamin D deficiency among adolescents, females had significantly higher prevalence of vitamin D deficiency compared to males even after adjusting for all potential confounders. Also, vitamin D level was significantly lower among females compared to males. This is similar to that reported among adolescents from Saudi Arabia [36], India [9], Korea [28] and Taiwan [37]. This pattern is not common in other settings as reported from a clinic-based cross-sectional study in US [25] or Italy [23]. In The Arab states in the Gulf region and India, cultural practices, such as type of clothing that cover whole body and indoor lifestyle, have been proposed to exacerbate vitamin D deficiency among females. Indeed, we found males consistently reporting higher amount of outdoor exposure to sunlight compared to females in addition to that females reported consistently higher sun avoidance behavior such as using sunscreen and staying in shade or under an umbrella. Adjusting for the exposure to sunlight did not explain the whole association between vitamin D status and gender which could be due residual

For peer review only - http://bmjopen.bmj.com/site/about/guidelines.xhtml

confounding. We enquired about the type of clothing among females using photo cards but vitamin D deficiency was too common among females, which did not allow for us to investigate the impact of type of dress on vitamin D status among females. In our setting, efforts to improve vitamin D level among adolescents should focus on females and the current recommended doses for vitamin D supplement should also take this into account and recommend higher doses for females compared to males.

Most of the students in Kuwait do not walk to schools and are mainly transported using private cars or school buses. The number of times the student walk to/from school as well as the time spent on walking to/from school was significantly associated with vitamin D deficiency. Those who walk to/from schools were less likely to have vitamin D deficiency compared to those who used school bus or private cars. Walking to schools can be a good opportunity for sunlight exposure but also as a physical activity itself may improve vitamin D level. In a study in USA, physical activity was inversely associated with hypovitaminosis D but not related to vitamin D level [25] while in another study, vigorous physical activity was significantly associated with vitamin D level [26]. It has been proposed that physical activity may increase the level of vitamin D through increasing the time spent outdoor in sunlight or through reducing the risk of obesity [11]. However, in our study the total time spent on other physical/sport activities was significantly associated with vitamin D deficiency in univariate but not in multivariate analysis. Unlike walking to the schools, most sport activities in our setting are practiced in closed areas or during night time due to severe hot weather which may explain the lack of association between total time spent on physical activities and vitamin D deficiency in multivariate analysis. Like many other studies [22, 25, 28], BMI was inversely associated with vitamin D deficiency.

In our study, sugary drinks were positively associated with the prevalence of vitamin D deficiency. This is similar to that reported from Saudi Arabia [34] and USA [25]. Consumption of sugary drinks among adolescents usually occur at the expense of milk consumption which contains vitamin D [38]. We have

For peer review only - http://bmjopen.bmj.com/site/about/guidelines.xhtml

noted this pattern in our data as those consuming sugary drinks were less likely to consume milk (data not shown). Although milk consumption was not related to vitamin D deficiency it was significantly related to the vitamin D level in linear regression analysis. It has been proposed that presence of vitamin D-fortified juices may help to alleviate the problem of vitamin D deficiency [25].

We found an association between passive smoking at household (reported by parents) and vitamin D deficiency among adolescents, which is interesting and have been reported before [37]. Only less than 1% of the adolescents in our setting reported active smoking. Smoking also has been reported to be a significant determinant for low vitamin D level among adults in some studies [24] but not in others [39] and this issue remains controversial [24]. Furthermore, a recent study found no significant association between cotinine level and vitamin D deficiency among Korean adolescents [28]. One of the hypothesized mechanism is that smoking may reflect a less healthy life style including less physical activity and bad dietary habits [24] but also a causal link has been proposed as metabolites in cigarette's smoke can inhibit CYP27A1 activity [40].

Strengths and limitation

This is the first study that measured vitamin D on a large nationally representative sample of adolescents in Kuwait. We used a measurement method that is recommended for epidemiological studies and gathered data from both parents and adolescents. However, we did not measure the skin color, which can be an important determinant for vitamin D formulation in skin during exposure to sunlight. Furthermore, there were adolescents with low vitamin D concentration which was not accompanied by secondary hyperparathyroidism (Figure 1). This has been noted in other studies [25, 34], the clinical significance of which is unknown in young children and deserves further study. We evaluated the dietary intake of vitamin D in only a subgroup of study participants. However, dietary intake of vitamin D is known to have a smaller

For peer review only

contribution to vitamin D status compared to cutaneous synthesis in response to sun exposure [35] and data on vitamin D supplement were obtained from all participants.

Conclusion

In conclusion, a high prevalence of vitamin D deficiency was noted among adolescents in Kuwait despite the abundant sunshine. This may reflect strong sun avoidance behavior as only less than 4% of adolescents were vitamin D sufficient and most of them were on vitamin D supplements. An optimal vitamin D level is essential during adolescents; and vitamin D deficiency at this age represents a public health problem that should be addressed. First, skepticism about the problem or its clinical significance may hinder efforts to put forward practical solutions. This cynicism particularly among scientific community and decision makers should be cleared. Second, sun exposure is the main source for vitamin D level in this age group, therefore adequate outdoor daytime activities should be encouraged especially for females. Slight modifications in school environment to facilitate exposure to sunlight during school breaks between classes can be fruitful. With respect to vitamin D supplementation, we call for locally tailored guidelines in which females should have a higher amount of vitamin D supplement compared to males. Vitamin D fortification particularly for food products that are popular for adolescents in our setting should be also considered. Increasing sunlight exposure and vitamin D fortification should not be considered mutually exclusive and that both strategies can be adopted.

Acknowledgements

The authors acknowledge the assistance of Nadien Sameeh Rushdi and the team of data collectors. Cooperation of all participating schools, and the facilitation of the project by the Ministry of Education is greatly acknowledged. We also acknowledge the support and cooperation of the staff and management of the United Genetics Laboratories, Kuwait in vitamin D analysis.

For peer review only

For peer review only - http://bmjopen.bmj.com/site/about/guidelines.xhtml

Authors' contributions

AT contributed to the study design and data collection, analyzed the data and drafted the paper. He had full access to all the data and took responsibility for the integrity of the data. AR contributed to the design of the study and data collection in addition to the writing and revising the manuscript. RS and LS contributed to the design of the study and data collection in addition to revising the manuscript with significant intellectual input. AH contributed to the data collection and revised the manuscript.

Funding: The study was supported and funded by Kuwait University, Research Sector (Project No. WF 02/13).

Competing interests: The authors declare that they have no competing interests

Data sharing statement: No additional data are available.

For peer review only

REFERENCES

1 Mouratidou T, Vicente-Rodriguez G, Gracia-Marco L, *et al.* Associations of dietary calcium, vitamin D, milk intakes, and 25-hydroxyvitamin D with bone mass in Spanish adolescents: the HELENA study. *Journal of clinical densitometry : the official journal of the International Society for Clinical Densitometry* 2013;**16**:110-7.

2 Sharief S, Jariwala S, Kumar J, *et al.* Vitamin D levels and food and environmental allergies in the United States: results from the National Health and Nutrition Examination Survey 2005-2006. *The Journal of allergy and clinical immunology* 2011;**127**:1195-202.

3 Lucato P, Solmi M, Maggi S, *et al.* Low vitamin D levels increase the risk of type 2 diabetes in older adults: A systematic review and meta-analysis. *Maturitas* 2017;**100**:8-15.

4 Bahrami A, Mazloum SR, Maghsoudi S, *et al.* High Dose Vitamin D Supplementation Is Associated With a Reduction in Depression Score Among Adolescent Girls: A Nine-Week Follow-Up Study. *Journal of dietary supplements* 2017:1-10.

5 Chowdhury R, Kunutsor S, Vitezova A, *et al.* Vitamin D and risk of cause specific death: systematic review and meta-analysis of observational cohort and randomised intervention studies. *BMJ (Clinical research ed)* 2014;**348**:g1903.

6 Hilger J, Friedel A, Herr R, *et al.* A systematic review of vitamin D status in populations worldwide. *The British journal of nutrition* 2014;**111**:23-45.

7 Basatemur E, Horsfall L, Marston L, *et al.* Trends in the Diagnosis of Vitamin D Deficiency. *Pediatrics* 2017;**139**.

8 Garg MK, Marwaha RK, Khadgawat R, *et al.* Efficacy of vitamin D loading doses on serum 25-hydroxy vitamin D levels in school going adolescents: an open label non-randomized prospective trial. *Journal of pediatric endocrinology & metabolism : JPEM* 2013;**26**:515-23.

9 Kapil U, Pandey RM, Goswami R, *et al.* Prevalence of Vitamin D deficiency and associated risk factors among children residing at high altitude in Shimla district, Himachal Pradesh, India. *Indian journal of endocrinology and metabolism* 2017;**21**:178-83.

10 AlBuhairan FS, Tamim H, Al Dubayee M, *et al.* Time for an Adolescent Health Surveillance System in Saudi Arabia: Findings From "Jeeluna". *The Journal of adolescent health : official publication of the Society for Adolescent Medicine* 2015;**57**:263-9.

11 Valtuena J, Gonzalez-Gross M, Huybrechts I, *et al.* Factors associated with vitamin D deficiency in European adolescents: the HELENA study. *Journal of nutritional science and vitaminology* 2013;**59**:161-71.

12 Glanz K, Yaroch AL, Dancel M, *et al.* Measures of sun exposure and sun protection practices for behavioral and epidemiologic research. *Archives of dermatology* 2008;**144**:217-22.

13 UEA. Youth Physical Activity Questionnaire. University of East Anglia 2011.

14 Al-Hazzaa HM, Al-Sobayel HI, Musaiger AO. Convergent validity of the Arab Teens Lifestyle Study (ATLS) physical activity questionnaire. *International journal of environmental research and public health* 2011;**8**:3810-20.

15 Taylor C, Lamparello B, Kruczek K, *et al.* Validation of a food frequency questionnaire for determining calcium and vitamin D intake by adolescent girls with anorexia nervosa. *Journal of the American Dietetic Association* 2009;**109**:479-85, 85 e1-3.

For peer review only

For peer review only - http://bmjopen.bmj.com/site/about/guidelines.xhtml

16 Papandreou D, achaniotis N, Lari M*, et al.* Validation of a Food Frequency Questionnaire for Vitamin D and Calcium Intake in Healthy Female College Students. *Food and Nutrition Sciences* 2014;**5**:2048-52.

17 Holick MF. Vitamin D deficiency. *The New England journal of medicine* 2007;**357**:266-81.

18 Vogeser M. Quantification of circulating 25-hydroxyvitamin D by liquid chromatography-tandem mass spectrometry. *The Journal of steroid biochemistry and molecular biology* 2010;**121**:565-73.

19 Holick MF, Binkley NC, Bischoff-Ferrari HA*, et al.* Evaluation, treatment, and prevention of vitamin D deficiency: an Endocrine Society clinical practice guideline. *The Journal of clinical endocrinology and metabolism* 2011;**96**:1911-30.

20 Medicine SfAHa. Recommended vitamin D intake and management of low vitamin D status in adolescents: a position statement of the society for adolescent health and medicine. *The Journal of adolescent health : official publication of the Society for Adolescent Medicine* 2013;**52**:801-3.

21 Braegger C, Campoy C, Colomb V*, et al.* Vitamin D in the healthy European paediatric population. *Journal of pediatric gastroenterology and nutrition* 2013;**56**:692-701.

22 Gonzalez-Gross M, Valtuena J, Breidenassel C*, et al.* Vitamin D status among adolescents in Europe: the Healthy Lifestyle in Europe by Nutrition in Adolescence study. *The British journal of nutrition* 2012;**107**:755-64.

23 Vierucci F, Del Pistoia M, Fanos M*, et al.* Vitamin D status and predictors of hypovitaminosis D in Italian children and adolescents: a cross-sectional study. *European journal of pediatrics* 2013;**172**:1607-17.

24 Kassi EN, Stavropoulos S, Kokkoris P*, et al.* Smoking is a significant determinant of low serum vitamin D in young and middle-aged healthy males. *Hormones (Athens, Greece)* 2015;**14**:245-50.

25 Gordon CM, DePeter KC, Feldman HA*, et al.* Prevalence of vitamin D deficiency among healthy adolescents. *Archives of pediatrics & adolescent medicine* 2004;**158**:531-7.

26 Dong Y, Pollock N, Stallmann-Jorgensen IS*, et al.* Low 25-hydroxyvitamin D levels in adolescents: race, season, adiposity, physical activity, and fitness. *Pediatrics* 2010;**125**:1104-11.

27 Rockell JE, Green TJ, Skeaff CM*, et al.* Season and ethnicity are determinants of serum 25-hydroxyvitamin D concentrations in New Zealand children aged 5-14 y. *The Journal of nutrition* 2005;**135**:2602-8.

28 Byun EJ, Heo J, Cho SH*, et al.* Suboptimal vitamin D status in Korean adolescents: a nationwide study on its prevalence, risk factors including cotinine-verified smoking status and association with atopic dermatitis and asthma. *BMJ open* 2017;**7**:e016409.

29 Zhang FF, Al Hooti S, Al Zenki S*, et al.* Vitamin D deficiency is associated with high prevalence of diabetes in Kuwaiti adults: results from a national survey. *BMC public health* 2016;**16**:100.

30 Al-Daghri NM. Vitamin D in Saudi Arabia: Prevalence,distribution and disease associations. *The Journal of steroid biochemistry and molecular biology* 2016.

31 Golbahar J, Al-Saffar N, Altayab Diab D*, et al.* Predictors of vitamin D deficiency and insufficiency in adult Bahrainis: a cross-sectional study. *Public health nutrition* 2014;**17**:732-8.

32 Mahdy S, Al-Emadi SA, Khanjar IA*, et al.* Vitamin D status in health care professionals in Qatar. *Saudi medical journal* 2010;**31**:74-7.

For peer review only - http://bmjopen.bmj.com/site/about/guidelines.xhtml

33 Thalib L, Al-Taiar A. Dust storms and the risk of asthma admissions to hospitals in Kuwait. *The Science of the total environment* 2012;**433**:347-51.

34 Sulimani RA, Mohammed AG, Alfadda AA*, et al.* Vitamin D deficiency and biochemical variations among urban Saudi adolescent girls according to season. *Saudi medical journal* 2016;**37**:1002-8.

35 Holick MF. The D-lightful vitamin D for child health. *JPEN Journal of parenteral and enteral nutrition* 2012;**36**:9S-19S.

36 Al-Daghri NM, Al-Saleh Y, Aljohani N*, et al.* Vitamin D Deficiency and Cardiometabolic Risks: A Juxtaposition of Arab Adolescents and Adults. *PloS one* 2015;**10**:e0131315.

37 Yao TC, Tu YL, Chang SW*, et al.* Suboptimal vitamin D status in a population-based study of Asian children: prevalence and relation to allergic diseases and atopy. *PloS one* 2014;**9**:e99105.

38 O'Connor TM, Yang SJ, Nicklas TA. Beverage intake among preschool children and its effect on weight status. *Pediatrics* 2006;**118**:e1010-8.

39 Scragg R, Holdaway I, Jackson R*, et al.* Plasma 25-hydroxyvitamin D3 and its relation to physical activity and other heart disease risk factors in the general population. *Annals of epidemiology* 1992;**2**:697-703.

40 Aboraia AS, Makowski B, Bahja A*, et al.* Synthesis and CYP24A1 inhibitory activity of (E)-2-(2-substituted benzylidene)- and 2-(2-substituted benzyl)-6-methoxy-tetralones. *European journal of medicinal chemistry* 2010;**45**:4427-34.

Figure legends

Figure (1): Parathyroid hormone (PTH) with Serum 25-hydroxyvitamin D (25-OH-D) concentration. Individuals above the horizontal line (PTH $\geq$ 65.0 ng/L) are adolescents with secondary hyperthyroidism. Vertical lines represent the 25-OH-D cut-off used to define vitamin D deficiency, insufficiency and sufficiency.

For peer review only - http://bmjopen.bmj.com/site/about/guidelines.xhtml

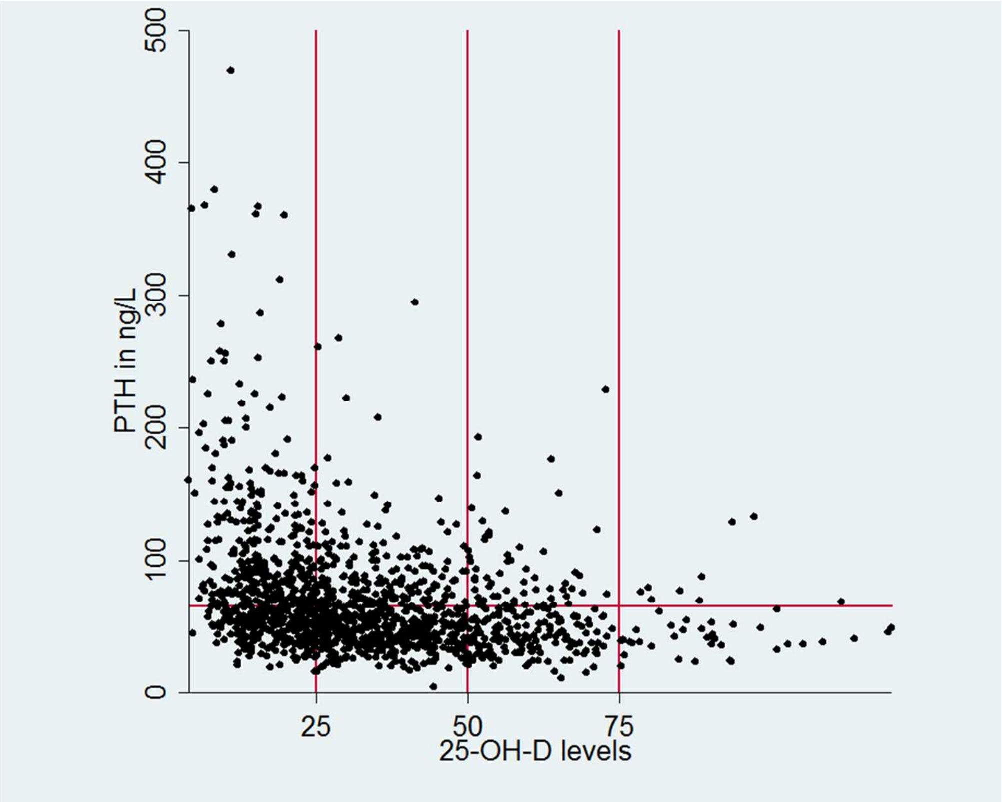

Figure (1): Parathyroid hormone (PTH) with Serum 25-hydroxyvitamin D (25-OH-D) concentration. Individuals above the horizontal line (PTH ≥ 65.0 ng/L) are adolescents with secondary hyperthyroidism. Vertical lines represent the 25-OH-D cut-off used to define vitamin D deficiency, insufficiency and sufficiency.

323x259mm (72 x 72 DPI)

Supplementary Table (1). Factors associated with vitamin D deficiency in univariate analysis, 1416 middle school students.

Characteristics	Prevalence n (%)	Prevalence Ratio [95%CI]	p
**Gender**			
Male	488 (70.32)	1 [Ref.]	<0.001
Female	662 (91.69)	1.30 [1.26- 1.33]	
**Age (year)**			
<12	409 (77.61)	1 [Ref.]	0.024
12-	369 (84.05)	1.07 [1.01- 1.12]	
≥13	372 (82.67)	1.06 [1.00- 1.10]	
**Nationality**			
Kuwaiti	900 (83.26)	1 [Ref.]	<0.001
Non-Kuwait	250 (74.63)	0.90 [0.82-0.96]	
**Governorate**[1]			
Capital	127 (81.41)	1 [Ref.]	0.003
Hawally	188 (76.42)	0.94 [0.82-1.03]	
Farawanya	183 (77.54)	0.95 [0.83-1.04]	
Jahra	214 (89.54)	1.10 [1.02-1.15]	
Mubarak al-Kabeer	124 (83.78)	1.03 [0.91-1.10]	
Ahmadi	296 (79.78)	0.98 [0.88-1.06]	
**Father's Education**[2]			
Primary/Intermediate/no formal education	205 (86.86)	1 [Ref.]	<0.001
Secondary (high school)	297 (86.34)	0.99 [0.87-1.08]	
Diploma	220 (84.29)	0.95 [0.82-1.06]	
University & above	399 (73.62)	0.78 [0.65-0.90]	
**Mother's Education**[3]			
Primary/Intermediate/no formal education	153 (83.61)	1 [Ref.]	<0.001
Secondary (high school)	263 (86.51)	1.04 [0.94-1.12]	
Diploma	259 (85.20)	1.02 [0.91-1.10]	
University & above	457 (75.54)	0.88 [0.76-0.99]	
**Total number of brother/sisters**[4]			
Zero-two	223 (74.83)	1 [Ref.]	0.002
Three-four	434 (80.67)	1.05 [1.00-1.10]	
Five or more	469 (84.81)	1.10 [1.04-1.13]	
**Passive smoking at household**[5]			
No	704 (78.14)	1 [Ref.]	<0.001
Yes	426 (87.12)	1.11 [1.06-1.15]	

Times per week consumed breakfast not prepared at home					
Zero	484	(77.56)	1	[Ref.]	0.019
One-two times	478	(84.01)	1.08	[1.02-1.12]	
Three-four	98	(83.76)	1.07	[0.97-1.14]	
Five or more	67	(85.90)	1.10	[1.00-1.17]	
**Times per week consumed dinner not prepared at home**					
Zero	114	(72.61)	1	[Ref.]	0.018
One-two times	719	(81.89)	1.09	[1.03-1.14]	
Three-four	211	(82.10)	1.09	[1.01-1.15]	
Five or more	75	(87.21)	1.14	[1.04-1.19]	
**Consumption of sugary drinks per week**					
None	123	(71.93)	1	[Ref.]	0.002
Once-Three times	633	(81.99)	1.09	[1.04-1.14]	
Four-Six	135	(78.95)	1.07	[0.98-1.13]	
Seven/more	256	(85.62)	1.12	[1.07-1.17]	
**Currently taking supplements[5]**					
No	1,045	(83.20)	1	[Ref.]	<0.001
Yes	103	(65.19)	0.78	[0.68-0.88]	
**Number of times walking to school per week[5]**					
None	982	(84.80)	1	[Ref.]	<0.001
1-8 times	99	(63.87)	0.74	[0.63-0.83]	
Every day	69	(66.99)	0.77	[0.65-0.89]	
**Time walking to school[5]**					
None	982	(84.80)	1	[Ref.]	<0.001
≤5 minutes	50	(62.50)	0.71	[0.56-0.84]	
6- 10 minutes	53	(69.74)	0.80	[0.65-0.93]	
11-15	38	(67.86)	0.77	[0.60-0.92]	
16 minutes or more	26	(57.78)	0.65	[0.46-0.82]	
**Time spent on physical activity per week**					
Low	396	(84.43)	1	[Ref.]	0.005
Medium	391	(82.66)	0.97	[0.89-1.04]	
High	363	(76.58)	0.89	[0.80-0.96]	
**Body Mass Index Categories[6]**					
Normal weight	465	(77.37)	1	[Ref.]	0.001
Overweight	270	(84.38)	1.07	[1.02-1.11]	
Obese	400	(84.93)	1.08	[1.03-1.11]	
Under weight	15	(62.50)	0.84	[0.59-1.02]	

**Time outside per day 10:00 am- 4:00 pm week days**					
Less than 30 minutes	563	(85.69)	1	[Ref.]	<0.001
31 minutes to 1 hour	342	(76.00)	0.87	[0.80-0.94]	
More than 1 hour to 2 hours	178	(80.18)	0.93	[0.84-1.00]	
More than 2 hours	67	(77.01)	0.88	[0.74-0.99]	
**Time outside per day 10:00 am- 4:00 pm week ends**					
Less than 30 minutes	425	(86.03)	1	[Ref.]	0.003
31 minutes to 1 hour	257	(82.37)	0.94	[0.85-1.02]	
More than 1 hour to 2 hours	198	(77.65)	0.87	[0.76-0.96]	
More than 2 hours to 3 hours	142	(76.76)	0.85	[0.74-0.96]	
More than 3 hours	127	(75.15)	0.83	[0.71-0.94]	
**Wearing sunscreen**					
Never	874	(79.45)	1	[Ref.]	
Rarely	65	(84.42)	1.06	[0.94-1.13]	
Sometimes	140	(87.50)	1.09	[1.02-1.14]	
Often/always	71	(89.87)	1.12	[1.02-1.18]	
**Staying in shade or under an umbrella**					
Never	396	(80.32)	1	[Ref.]	
Rarely	70	(72.16)	0.90	[0.78-1.01]	
Sometimes	301	(82.92)	1.03	[0.96-1.08]	
Often	162	(79.02)	0.98	[0.90-1.05]	
Always	220	(85.60)	1.06	[0.99-1.11]	

[1]Missing for 20 participants; [2] Missing for 33 participants; [3] Missing for 20 participants; [4]Missing for 27 participants; [5] Missing for 2 participants; [6]Missing for 26 participants; [7] according to WHO growth charts.

For peer review only - http://bmjopen.bmj.com/site/about/guidelines.xhtml

STROBE 2007 (v4) checklist of items to be included in reports of observational studies in epidemiology*

Checklist for cohort, case-control, and cross-sectional studies (combined)

Section/Topic	Item #	Recommendation	Reported on page #
**Title and abstract**	1	(*a*) Indicate the study's design with a commonly used term in the title or the abstract	Page 1
		(*b*) Provide in the abstract an informative and balanced summary of what was done and what was found	Page 3
**Introduction**			
Background/rationale	2	Explain the scientific background and rationale for the investigation being reported	Page 5
Objectives	3	State specific objectives, including any pre-specified hypotheses	
**Methods**			
Study design	4	Present key elements of study design early in the paper	
Setting	5	Describe the setting, locations, and relevant dates, including periods of recruitment, exposure, follow-up, and data collection	Page 6- Page 8 and Page 14
Participants	6	(*a*) *Cohort study*—Give the eligibility criteria, and the sources and methods of selection of participants. Describe methods of follow-up<br>*Case-control study*—Give the eligibility criteria, and the sources and methods of case ascertainment and control selection. Give the rationale for the choice of cases and controls<br>*Cross-sectional study*—Give the eligibility criteria, and the sources and methods of selection of participants	Page 6
		(*b*) *Cohort study*—For matched studies, give matching criteria and number of exposed and unexposed<br>*Case-control study*—For matched studies, give matching criteria and the number of controls per case	
Variables	7	Clearly define all outcomes, exposures, predictors, potential confounders, and effect modifiers. Give diagnostic criteria, if applicable	Page 6 and Page 7
Data sources/ measurement	8*	For each variable of interest, give sources of data and details of methods of assessment (measurement). Describe comparability of assessment methods if there is more than one group	
Bias	9	Describe any efforts to address potential sources of bias	
Study size	10	Explain how the study size was arrived at	
Quantitative variables	11	Explain how quantitative variables were handled in the analyses. If applicable, describe which groupings were chosen and why	Page 7 and Page 8
Statistical methods	12	(*a*) Describe all statistical methods, including those used to control for confounding	Page 7 and Page 8
		(*b*) Describe any methods used to examine subgroups and interactions	Page 7 and Page 8
		(*c*) Explain how missing data were addressed	
		(*d*) *Cohort study*—If applicable, explain how loss to follow-up was addressed<br>*Case-control study*—If applicable, explain how matching of cases and controls was addressed	Page 8

For peer review only

		Cross-sectional study—If applicable, describe analytical methods taking account of sampling strategy	
		(*e*) Describe any sensitivity analyses	
**Results**			
Participants	13*	(a) Report numbers of individuals at each stage of study—eg numbers potentially eligible, examined for eligibility, confirmed eligible, included in the study, completing follow-up, and analysed	Page 8
		(b) Give reasons for non-participation at each stage	Page 8
		(c) Consider use of a flow diagram	
Descriptive data	14*	(a) Give characteristics of study participants (eg demographic, clinical, social) and information on exposures and potential confounders	Table 1 page 9
		(b) Indicate number of participants with missing data for each variable of interest	Table 1 , Table 2 and also supplementary table
		(c) *Cohort study*—Summarise follow-up time (eg, average and total amount)	
Outcome data	15*	*Cohort study*—Report numbers of outcome events or summary measures over time	
		Case-control study—Report numbers in each exposure category, or summary measures of exposure	
		Cross-sectional study—Report numbers of outcome events or summary measures	Page 10
Main results	16	(*a*) Give unadjusted estimates and, if applicable, confounder-adjusted estimates and their precision (eg, 95% confidence interval). Make clear which confounders were adjusted for and why they were included	
		(*b*) Report category boundaries when continuous variables were categorized	All tables
		(*c*) If relevant, consider translating estimates of relative risk into absolute risk for a meaningful time period	
Other analyses	17	Report other analyses done—eg analyses of subgroups and interactions, and sensitivity analyses	Page 12
**Discussion**			
Key results	18	Summarise key results with reference to study objectives	Page 13
Limitations	19	Discuss limitations of the study, taking into account sources of potential bias or imprecision. Discuss both direction and magnitude of any potential bias	Page 16
Interpretation	20	Give a cautious overall interpretation of results considering objectives, limitations, multiplicity of analyses, results from similar studies, and other relevant evidence	Page 17
Generalisability	21	Discuss the generalisability (external validity) of the study results	Page 17
**Other information**			
Funding	22	Give the source of funding and the role of the funders for the present study and, if applicable, for the original study on which the present article is based	Page 18

*Give information separately for cases and controls in case-control studies and, if applicable, for exposed and unexposed groups in cohort and cross-sectional studies.

For peer review only

Note: An Explanation and Elaboration article discusses each checklist item and gives methodological background and published examples of transparent reporting. The STROBE checklist is best used in conjunction with this article (freely available on the Web sites of PLoS Medicine at http://www.plosmedicine.org/, Annals of Internal Medicine at http://www.annals.org/, and Epidemiology at http://www.epidem.com/). Information on the STROBE Initiative is available at www.strobe-statement.org.

For peer review only

BMJ Open

Vitamin D Status among Adolescents in Kuwait: A Cross-sectional Study.

Journal:	*BMJ Open*
Manuscript ID	bmjopen-2017-021401.R1
Article Type:	Research
Date Submitted by the Author:	09-Mar-2018
Complete List of Authors:	Al-Taiar, Abdullah; Faculty of Medicine, Kuwait University, Department of Community Medicine and Behavioural Sciences<br>Rahman , Abdur ; College of Life Sciences, Kuwait University, Department of Food Science and Nutrition<br>Al-Sabah, Reem; Faculty of Medicine, Kuwait University, Department of Community Medicine and Behavioural Sciences<br>Shaban, Lemia ; College of Life Sciences, Kuwait University, Department of Food Science and Nutrition<br>Al-Harbi, Anwar ; Kuwait Institute for Scientific Research , Department of Science and Nutrition
**Primary Subject Heading**:	Nutrition and metabolism
Secondary Subject Heading:	Epidemiology, Global health, Paediatrics, Public health
Keywords:	PUBLIC HEALTH, Vitamin D, Adolescents, school children, Kuwait

SCHOLARONE™
Manuscripts

Vitamin D Status among Adolescents in Kuwait: A Cross-sectional Study.

Abdullah Al-Taiar[1], Abdur Rahman[2], Reem Al-Sabah[1], Lemia Shaban[2], Anwar Al-Harbi[3]

[1] Department of Community Medicine and Behavioural Sciences

Faculty of Medicine, Kuwait University,

Box: 24923 Safat-13110 Kuwait.

[2] Department of Food Science and Nutrition

College of Life Sciences, Kuwait University,

Box 5969, Safat-13060, Kuwait.

[3] Kuwait Institute for Scientific Research,

Department of Science and Nutrition,

Box: 24885 Safat-13109, Kuwait.

Before publication corresponding author:

Dr Abdullah Al-Taiar

Associate Professor of Epidemiology

Department Community Medicine and Behavioural Sciences,

Faculty of Medicine, Kuwait University,

Box: 24923 Safat-13110 Kuwait.

Tel: +965-24986553

Fax: +965-5338948

E-mail: altaiar@hsc.edu.kw

For peer review only

For peer review only - http://bmjopen.bmj.com/site/about/guidelines.xhtml

After publication corresponding author:

Abdur Rahman

Associate Professor

Department of Food Science and Nutrition,

College of Life Sciences, Kuwait University,

Box: 5969 Safat-13060 Kuwait.

Tel: +965-249633055

Fax: +965-22513929

E-mail: abdurrahman.ahmad@ku.edu.kw

For peer review only

Abstract

Objectives

In Kuwait, as in many Arab states in the gulf region, there are limited data on the prevalence of vitamin D deficiency among healthy adolescents. This study aimed to estimate the prevalence of vitamin D deficiency in a nationally representative sample of adolescents and investigate factors associated with vitamin D status.

Methods

A cross-sectional study was conducted on 1416 adolescents aged 11-16 years, who were randomly selected from middle schools in all governorates of Kuwait. Data were collected from parents through self-administered questionnaire and from adolescents through face-to-face interview. Vitamin D was measured using liquid chromatography-tandem mass spectrometry (LC-MS/MS). Logistic regression was used to investigate the independent factors associated with vitamin D status.

Results

The Prevalence of vitamin D deficiency was 1,150 (81.21%, 95%CI: 71.61-90.81%) while severe deficiency was 559 (39.48%). Only 51 (3.60%) were vitamin D sufficient. Prevalence was significantly higher among females compared to males (91.69% vs. 70.32%; p<0.001). There was a significant inverse correlation between vitamin D and PTH (Spearman correlation=-0.35; p<0.001). In the final model, gender, age, governorate, parental education, body mass index, vitamin D supplement and number of times walking to school per week were all significantly related to vitamin D deficiency.

Conclusion

High prevalence of vitamin D deficiency was noted among adolescents in Kuwait despite the abundant sunshine which may reflect strong sun avoidance behavior. Adequate outdoor daytime activities should be encouraged especially for females. We call for locally tailored guidelines for vitamin D supplement in which females should have a higher dose compared to males.

Keywords: Vitamin D, adolescents, Kuwait, Middle East, school children

For peer review only - http://bmjopen.bmj.com/site/about/guidelines.xhtml

Strengths and limitations of this study

- This is the first study that aimed to estimate the prevalence of vitamin D on a large nationally representative sample of adolescents in Kuwait.
- We measured vitamin D using the recommended laboratory methodology and gathered data from both parents and adolescents.
- We did not measure the skin color, which can be an important determinant for vitamin D synthesis in skin.

For peer review only

For peer review only - http://bmjopen.bmj.com/site/about/guidelines.xhtml

Introduction

An optimal vitamin D status is extremely important during adolescence for proper growth and bone mineral accrual [1]. In addition to its critical role in bone health, several studies have linked vitamin D deficiency with various disease conditions during adolescence or adulthood such as asthma and allergies [2], type 2 diabetes [3], depression[4], cancer and even all-cause mortality [5]. However, the results of randomized control trials, which tested the impact of vitamin D supplement on the risk of these disease conditions, remain inconclusive.

Several studies have evaluated the prevalence of vitamin D deficiency and insufficiency during adolescence reporting high prevalence of vitamin D deficiency worldwide [6, 7]. This was the case even in countries with abundant sunshine, which is the main factor for endogenous vitamin D synthesis. For example, in India the prevalence of vitamin D deficiency among adolescents was reported to be 85-98% [8, 9] while in Saudi Arabia it was found to be around 96% [10]. In Arab states in the Gulf region or the broader Middle East, there is paucity of data on vitamin D status in pediatric and adolescents populations. The few studies that showed a high prevalence of vitamin D deficiency suffered from major methodological weaknesses [10-12]. These studies were small and hospital-based, lacked proper sampling technique or used less optimal laboratory method to measure vitamin D. It is also known that factors which influence vitamin D status such as avoiding sun exposure, skin pigmentation, high body mass index (BMI) and dietary factors, particularly the lack of vitamin D supplement and intake of vitamin D rich foods [13] are all on rise in Arab states in the Gulf region. The aim of the present study was therefore to assess vitamin D status among adolescents living in Kuwait, a country with a plenty of sun shine, and investigate factors associated with low vitamin D level.

For peer review only

Methods

Study population and study participants

Kuwait is a small country at latitude (29.3759° N) with a population of 4.2 million. Approximately 25% of population is under the age of 19-year. The study population comprised students in the age between 11 and 16 years in the public middle schools. School based cross-sectional study was conducted on students from 12 public middle schools that were selected using a stratified multistage cluster random sampling with a probability proportional to size from all governorates of Kuwait. The sample allocation in each governorate was based on the relative size of that governorate as judged by the total number of students in the governorate. Students with major chronic disease conditions are registered in each school and these were excluded from the study. However, students with minor illnesses or self-reported illness were included in the study. The type of illness was recorded during data collection and included in the analysis. The study was funded by Kuwait University and approved by The Ethics Committee at The Ministry of Health in Kuwait as well as The Ethics Committee at Health Sciences Centre, Kuwait University.

Data Collection

An informed consent was sent to the parents along with a self-administered questionnaire on parents' level of education (No formal education, primary/intermediate, high school, diploma, university and above), income, type of housing, number of siblings of the index child, passive smoking at household and the number of times per week the index child had a meal prepared outside home during the last three months. After obtaining written informed consent from the parents and verbal consent from the school children, trained dedicated personnel carried out face-to-face interview with the students using a structured questionnaire. The questionnaire was carefully developed after extensive review of the literature and was pilot tested on 20 students who were not included in the study. It comprised questions on habitual sun exposure during the last three months, which was assessed by the core questions developed to measure

For peer review only - http://bmjopen.bmj.com/site/about/guidelines.xhtml

the exposure to sunlight in adolescents as described by Glanz et al. [14]; in addition to smoking habits, physical activity and dietary intake. Data on physical activity were collected using a questionnaire that was developed based on the Youth Physical Activity Questionnaire in UK [15] and The Arab teens lifestyle study [16]. The questionnaire was validated among high school students and showed strong correlation with data collected by accelerometers (Spearman correlation 0.92; p<0.001 for total steps count) (not published). Data on dietary intake of vitamin D were collected from only 200 students using food frequency questionnaire for calcium and vitamin D intake in adolescents [17], which has been validated in our setting [18]. Food models or serving containers were used to assist in estimating serving size. Measurements of standing height and body weight of the study subjects were assessed using a digital weight and height scale (Detecto®) in a standardized manner.

Laboratory methods

Five ml of venous blood was collected in gel-containing tubes (SST II advance, BD vacutainer) from each child by a trained nurse; and the samples were protected from light. On the same day, the samples were centrifuged at 2000 x g for 15 minutes and the serum was transferred to Eppendorf tubes and stored at -80°C until analysis. Serum 25-hydroxyvitamin D (25-OH-D) concentration is the best marker of vitamin D status as it is the major circulating form of vitamin D, reflecting both the amount produced in the skin after sun exposure and the amount consumed in foods [19]. It was measured in College of American Pathologists CAP-accredited laboratory by liquid chromatography-tandem mass spectrometry (LC-MS/MS), which is the recommended method for vitamin D assessment in epidemiological studies [20]. According to the Endocrine Society [21] and the Society for Adolescent Health and Medicine [22], we used the following cut-off of 25-OH-D to define vitamin D status: vitamin D deficiency < 50 nmol/L (20 ng/mL); vitamin D insufficiency 50-75 nmol/L (20-30 ng/mL); and vitamin D sufficiency≥ 75 nmol/L (30 ng/mL). Thus, hypovitaminosis D was defined in the presence of 25-OH-D levels < 75.0 nmol/L (30 ng/mL). Furthermore, severe vitamin D deficiency was defined as 25-OH-D levels < 25.0 nmol/L (10 ng/mL) [23]. Serum intact Parathyroid hormone

For peer review only - http://bmjopen.bmj.com/site/about/guidelines.xhtml

(PTH) was measured using the Access Intact PTH chemiluminescent immunoassay with the Unicel DxI 800 Beckman Coulter analyzer using commercial kit (Cat. # A16972). Similar to other studies, PTH level ≥ 65.0 ng/L was suggestive of hyperparathyroidism [24] although there is no consensus on this cut-off point.

Patient and Public Involvement

Public were not directly involved in the design of this study. However, schools were involved in approaching the parents to obtain their consents. We also sent the results of the laboratory tests of each participant to the parents through schools using closed envelops to ensure confidentiality.

Data analysis

Data were double entered into specifically designed database using Epidata Entry. Data analysis was conducted using Stata (StataCorp. 2011. Release 12). BMI was calculated as weight (Kg) divided by height squared (m^2). Weight status was categorized into normal, overweight and obese according to WHO growth charts. Chi-squared test was used to test differences in categorical variables. A modified version of multiple logistic regression that calculates prevalence ratio was used to study the association between each presumed risk factor and vitamin D deficiency (25-OH-D < 50 nmol/L). This is because vitamin D deficiency was a common outcome and using log-binomial regression was not possible because the model failed to converge. Variables that were found to be statistically significant at 15% level of significance were considered in multivariate analysis. Variables were divided into groups (e.g. socio-demographic, sun exposure, etc) and were added sequentially to the model. Statistical significance of variables was evaluated by likelihood ratio test which compares the model with and without the variable. Goodness of fit of the final model was evaluated by Hosmer-Lemeshow test. Factors with $p<0.05$ were deemed to be statistically significant. The analysis above was repeated using hypovitaminosis (25-OH-D levels < 75.0 nmol/L) as the binary outcome. We also conducted multiple linear regression analysis using vitamin D level as a

For peer review only - http://bmjopen.bmj.com/site/about/guidelines.xhtml

continuous outcome (after log-transformation) and reported the results in the text. Because of the complex structure of this survey data, we used survey method, which gives more precise estimates of standard error.

Results

Of 1583 parents approached, 161 refused to participate (because children, parents or both refused). Another 6 samples were not sufficient to conduct blood analysis, thus the analysis below comprised 1416 students. Table (1) shows the socio-demographic characteristics of the study group. The mean (SD) age was 12.48 (0.94) years and 694 (49.01%) were males.

Table (1). Socio-demographic characteristics of 1416 adolescents in public middle schools in Kuwait.

**Characteristics**		
**Age in years,** Mean (SD) years	12.48	0.94
	**n**	**(%)**
**Gender**		
Male	694	(49.01)
**Nationality**		
Kuwaiti	1,081	(76.34)
Non-Kuwait	335	(23.66)
**Father's Education**[1]		
No formal education	15	(1.08)
Primary/Intermediate	221	(15.98)
Secondary (high school)	344	(24.87)
Diploma	261	(18.87)
University & above	542	(39.19)
**Mother's Education**[2]		
No formal education	31	(2.22)
Primary/Intermediate	152	(10.89)
Secondary (high school)	304	(21.78)
Diploma	304	(21.78)
University & above	605	(43.34)
**Father's Income**[3] (Kuwaiti Dinars)		
Less than 500	91	(6.64)
500 to 1000	304	(22.19)
1001 to 1500	421	(30.73)
1501 to 2000	219	(15.99)
More than 2000	173	(12.63)
Do not wish to tell	162	(11.82)
**Mother's Employment Status**[4]		

For peer review only - http://bmjopen.bmj.com/site/about/guidelines.xhtml

Housewife	488 (35.16)
Paid employment	680 (48.99)
Others	220 (15.85)
**Housing**[5]	
Rented flat	510 (36.51)
Rented house	163 (11.67)
Owned flat	59 (4.22)
Owned house	665 (47.60)

[1]Missing for 33 participants; [2] Missing for 20 participants; [3] Missing for 46 participants; [4]Missing for 28 participants; [5]Missing for 19 participants

For peer review only

For peer review only - http://bmjopen.bmj.com/site/about/guidelines.xhtml

Prevalence of vitamin D deficiency

The median (Interquartile Range, IQR) of 25-OH-D was 29.7 (19.2- 44.2) nmol/L. Prevalence of vitamin D deficiency was 1,150 (81.21%, 95%CI: 71.61-90.81%) while severe deficiency was 559 (39.48%). Only 51 (3.60%) participants were vitamin D sufficient. Prevalence of vitamin D was significantly higher among females compared to males (91.69% vs. 70.32%; p<0.001). Also, the median (IQR) of vitamin D was 39.8 (29.4- 52.7) nmol/L and 21.5 (14.7- 30.7) nmol/L among males and females, respectively (p<0.001). There was no difference in the prevalence of vitamin D deficiency between Kuwaitis and non-Kuwaitis after stratification by gender. There was significant inverse correlation between vitamin D and PTH (Spearman correlation=-0.35; p<0.001). In relation to vitamin D status, elevated PTH (secondary hyperparathyroidism PTH ≥65 ng/L. i.e. 6.89 pmol/L) was detected in 312 (55.81%), 185 (31.30%) and 59 (27.57%) of adolescents with vitamin D severe deficiency, deficiency and insufficiency, respectively. If we used the reference range of the laboratory where the samples were tested (PTH: 1.3-9.3 pmol/L), the prevalence of secondary hyperparathyroidism would be 182 (32.56%), 73 (12.35%), and 25 (11.68%) among adolescents with vitamin D severe deficiency, deficiency and insufficiency, respectively. The median (IQR) PTH was significantly different dependent on vitamin D status (severe deficiency 7.37 [5.44-10.55] pmol/L; deficiency 5.63[4.13-7.40] pmol/L; insufficiency 5.14[3.87-6.99] pmol/L; sufficiency 4.31[3.75- 5.73] pmol/L; p<0.001). The actual relationship between 25-OH-D and PTH levels is shown in figure (1).

Table (2): Factors associated with vitamin D deficiency in multivariate analysis, 1416 middle school students.

Characteristics	Prevalence of vitamin D deficiency n (%)	Prevalence Ratio [95%CI]	P
**Gender**			
Male	488 (70.32)	1 [Ref.]	<0.001
Female	662 (91.69)	1.20 [1.17- 1.21]	
**Age (year)**			
<12	409 (77.61)	1 [Ref.]	0.003
12-	369 (84.05)	1.10 [1.04- 1.14]	

For peer review only - http://bmjopen.bmj.com/site/about/guidelines.xhtml

≥13	372	(82.67)	1.07	[1.01- 1.12]	
**Governorate[1]**					
Capital	127	(81.41)	1	[Ref.]	<0.001
Hawally	188	(76.42)	0.88	[0.73-1.01]	
Farawanya	183	(77.54)	0.85	[0.68-0.99]	
Jahra	214	(89.54)	1.10	[0.98-1.17]	
Mubarak al-Kabeer	124	(83.78)	1.06	[0.93-1.14]	
Ahmadi	296	(79.78)	1.06	[0.95-1.13]	
**Father's Education[2]**					
Primary/Intermediate/no formal education	205	(86.86)	1	[Ref.]	<0.009
Secondary (high school)	297	(86.34)	1.04	[0.92-1.12]	
Diploma	220	(84.29)	1.01	[0.88-1.10]	
University & above	399	(73.62)	0.89	[0.76-1.01]	
**Passive smoking in household[3]**					
No	704	(78.14)	1	[Ref.]	<0.006
Yes	426	(87.12)	1.08	[1.02-1.13]	
**Currently taking supplements[4]**					
No	1,045	(83.20)	1	[Ref.]	<0.001
Yes	103	(65.19)	0.63	[0.49-0.76]	
**Consumption of sugary drinks per week**					
None	123	(71.93)	1	[Ref.]	0.002
Once-Three times	633	(81.99)	1.11	[1.05-1.16]	
Four-Six times	135	(78.95)	1.07	[0.96-1.14]	
Seven/more times	256	(85.62)	1.15	[1.08-1.19]	
**Number of times walking to school per week[5]**					
None	982	(84.80)	1	[Ref.]	<0.001
1-8 times	99	(63.87)	0.81	[0.68-0.93]	
Every day	69	(66.99)	0.77	[0.61-0.92]	
**Body Mass Index Categories[6]**					
Normal weight	465	(77.37)	1	[Ref.]	0.001
Overweight	270	(84.38)	1.08	[1.01-1.13]	
Obese	400	(84.93)	1.12	[1.07-1.16]	
Underweight	15	(62.50)	0.93	[0.67-1.10]	

[1]Missing for 20 participants; [2] Missing for 33 participants; [3]Missing for 26 participants; [4] Missing for 2 participants; [5] Missing for 26 participants;[6] according to WHO growth charts.

For peer review only - http://bmjopen.bmj.com/site/about/guidelines.xhtml

Factors associated with vitamin D status

Factors that showed significant association with vitamin D deficiency (25-OH-D < 50 nmol/L) in univariate analysis are shown in Supplementary Table. Table (2) shows the independent predictors for vitamin D deficiency in multivariate analysis. In the final model, gender, age, governorate, parental education, vitamin D supplement, and the number of times participant walks to school per week were all significantly related to vitamin D deficiency. Data from the food frequency questionnaire for calcium and vitamin D intake were available only for 200 study subjects, and of all food items, only eggs were significantly related to vitamin D deficiency in multivariate model (data not shown). We repeated the analysis above using hypovitaminosis (sufficient vs. insufficient/deficient) as a binary outcome. The same factors were found to be significantly related to hypovitaminosis, although mother's education (instead of father's education) was found to be significant in this analysis. We also used linear regression to identify the factors associated with vitamin D level as a continuous variable. Factors that showed significant association with vitamin D level in multivariate analysis were age, gender, governorate, passive smoking at household, number of times of having breakfast meal outside home in the last three months, time spent outdoor during weekdays, preference to stay in shade or under the umbrella, and BMI categories. With respect to dietary factors, milk and cheese, fast food hamburger and tuna fish were significantly associated with vitamin D level in univariate linear regression. When these food items were introduced to the final model, only milk and tuna fish were significantly associated with vitamin D level. It is worth noting that in this analysis the best model was able to explain only 45% of the variability in vitamin D level.

Discussion

Although several reports have described vitamin D deficiency among adults in the Arab states in the Gulf region, there are limited data on the prevalence of vitamin D deficiency among healthy adolescents. Our study used a nationally representative sample to examine a large number of healthy adolescents, becoming to our knowledge the largest study focusing on this group in Kuwait. We used the recommended

For peer review only - http://bmjopen.bmj.com/site/about/guidelines.xhtml

method to measure vitamin D level and demonstrated that vitamin D deficiency is too common despite the abundant sunshine. Secondary hyperparathyroidism due to vitamin D deficiency is also common and females had lower vitamin D level compared to males.

In our setting, approximately, 81% and 15% of adolescents were vitamin D deficient or insufficient, respectively. This is higher than that reported among adolescents in European countries [25-27], Southeastern United States [28, 29] or New Zealand [30]. Our finding is more akin to that reported from Saudi Arabia (95.6%) [10], India (85-98%) [8, 9], or Korea where only 2.3% of adolescents were found to be vitamin D sufficient [31]. Our findings are also in accordance with the high prevalence reported consistently among adult population in Kuwait [32] or other countries in the Gulf region such as Saudi Arabia [33], Bahrain [34] and Qatar [35].

Similar to other studies among adolescents [24, 28, 36], our findings showed that large number of those with low vitamin D level had evidence of secondary hyperparathyroidism. We reported a much higher prevalence of secondary hyperparathyroidism compared to other studies [24, 36], which could be due to using lower PTH cut-off point to define secondary hyperthyroidism or using different laboratory method to measure PTH. Hyperparathyroidism, which potentially lead to increased bone resorption, is particularly important during adolescence because it is the critical period to achieve Peak Bone Mass. Serum PTH decreased as 25-OH-D level increased with no clear threshold of serum 25-OH-D at which PTH level plateaued although very few participants had elevated PTH when 25-OH-D level was above 75 nmol/L (Figure 1). Although we did not measure markers of bone turnover, this provides an additional evidence that 25-OH-D should be maintained above 75 nmol/L, which is the current cut-off point used to define vitamin D sufficiency in adolescents [21, 22].

For peer review only

For peer review only - http://bmjopen.bmj.com/site/about/guidelines.xhtml

Because of the abundant sunshine in the Arab states in the Gulf region, there has been a lot of skepticism of vitamin D deficiency. Informal discussions, even among scientific community, in the region show strong denial attributing such low levels of vitamin D to poorly executed or faulty laboratory measurements, or any other reason. Our findings should eliminate doubts on vitamin D deficiency among adolescents in Kuwait and pave the way to look for practical solutions. Factors that may hinder cutaneous synthesis of vitamin D despite abundant sunshine may include dust storms (occur almost in one third of the year in Kuwait [37]) and an avoidance of sun exposure by indoor lifestyle, head covering or other cultural practices in addition to the use of sunscreen. Self-reported exposure to sunlight was a significant predictor for vitamin D deficiency in univariate analysis but lost statistical significance in multivariate analysis. Most of adolescents in our setting avoid sunlight as only around 6% of the participants spent more than two hours outdoor per day between 10:00 am and 4:00 pm during weekdays (data not shown). Avoidance of sunlight has been suggested to be the underlying reason for the counterintuitive seasonal variation that has been reported in the region, in which the prevalence of vitamin D deficiency is higher in the summer compared to the winter season [36]. Owing to the desert climate, avoiding sun exposure would be stronger during summer due to extremely high temperature. We collected blood samples in February, March and April and thus we were unable to describe seasonal variation in vitamin D status. Exposure to sunlight contributes up to 90–95% of the vitamin D supply, while the number of foods naturally containing a significant quantity of vitamin D is very limited except for some oily fish that is rarely consumed by adolescents worldwide and in our setting [38] (~5% of adolescents in our setting consumed salmon once per week or more). As such, education of the parents and adolescents on the safe amount of sun exposure in addition to changes in the school environment to facilitate exposure to sunlight may produce the highest impact on vitamin D status among adolescents. There is no consensus on the amount of time in which adolescents can safely be exposed to direct sunlight. However, exposure of legs and arms for at least 15 minutes two times per week has been reported to be sufficient for adequate sun-induced cutaneous vitamin D synthesis in adolescents [38].

For peer review only - http://bmjopen.bmj.com/site/about/guidelines.xhtml

Like several studies that investigated vitamin D deficiency among adolescents, females had a significantly higher prevalence of vitamin D deficiency compared to males even after adjusting for all potential confounders. Also, vitamin D level was significantly lower among females compared to males. This is similar to that reported among adolescents from Saudi Arabia [39], India [9], Korea [31] and Taiwan [40]. This pattern is not common in other settings as reported from a clinic-based cross-sectional study in the US [28] or Italy [26]. In the Arab states in the Gulf region and India, cultural practices, such as type of clothing that covers the whole body and indoor lifestyle, have been proposed to exacerbate vitamin D deficiency among females. Indeed, we found males consistently reporting higher amount of outdoor exposure to sunlight compared to females. In addition, females reported consistently higher sun avoidance behavior such as using sunscreen and staying in shade or under an umbrella. Adjusting for the exposure to sunlight did not explain the whole association between vitamin D status and gender which could be due to residual confounding. We also collected data on the type of clothing among females using photo cards (no head covering, head covering, head and face covering), but vitamin D deficiency was too common among females, which did not allow for us to investigate the impact of type of dress on vitamin D status among females. In our setting, efforts to improve vitamin D level among adolescents should focus on females through encouragement of safe amount of exposure to sunlight and intake of vitamin D rich foods. Also the current recommended doses for vitamin D supplement should take the difference between females and males into account and recommend higher doses for females compared to males.

Most of the students in Kuwait do not walk to school and are mainly transported using cars or school buses. The number of times student walk to/from school as well as the time spent on walking to/from school was significantly associated with vitamin D deficiency. Those who walk to/from schools were less likely to have vitamin D deficiency compared to those who used school bus or cars. Walking to schools can be a good opportunity for sunlight exposure but also as a physical activity itself may improve vitamin D level. In a study in the US, physical activity was inversely associated with hypovitaminosis D but not related to vitamin

For peer review only - http://bmjopen.bmj.com/site/about/guidelines.xhtml

D level [28] while in another study, vigorous physical activity was significantly associated with vitamin D level [29]. It has been proposed that physical activity may increase the level of vitamin D through increasing the time spent outdoor in sunlight or through reducing the risk of obesity [13]. However, in our study the total time spent on other physical/sport activities was significantly associated with vitamin D deficiency in univariate but not in multivariate analysis. Unlike walking to the schools, most sport activities in our setting are practiced indoor or during night time due to severe hot weather which may explain the lack of association between total time spent on physical activities and vitamin D deficiency in multivariate analysis. Like many other studies [25, 28, 31], BMI was inversely associated with vitamin D level, which has been attributed to sequestration of the this fat-soluble vitamin within the plentiful adipose tissue [41, 42]. It has also been suggested that leptin, an adipocyte-derived hormone, might activate a pathway that inhibits renal synthesis of the active form of vitamin D [43].

In our study, sugary drinks were positively associated with the prevalence of vitamin D deficiency. This is similar to that reported from Saudi Arabia [36] and US [28]. Consumption of sugary drinks among adolescents usually occurs at the expense of milk consumption which contains vitamin D [44]. As a result, it has been proposed that presence of vitamin D-fortified juices may help to reduce vitamin D deficiency in adolescents [28]. In our data those who consumed sugary drinks were less likely to consume milk (data not shown). Although milk consumption was not related to vitamin D deficiency it was significantly related to the vitamin D level in linear regression analysis.

We found an association between passive smoking at household (reported by parents) and vitamin D deficiency among adolescents, which is interesting and has been reported before [40]. Only less than 1% of the adolescents in our setting reported active smoking. Smoking has also been reported to be a significant determinant for low vitamin D level among adults in some studies [27] but not in others [45] and this issue remains controversial [27]. Furthermore, a recent study found no significant association between cotinine

For peer review only - http://bmjopen.bmj.com/site/about/guidelines.xhtml

level and vitamin D deficiency among Korean adolescents [31]. One of the hypothesized mechanism is that smoking may reflect a less healthy life style including less physical activity and poor dietary habits [27] but also a causal link has been proposed as metabolites in cigarette's smoke can inhibit CYP27A1 activity [46].

Strengths and limitation

This is the first study that measured vitamin D in a large nationally representative sample of adolescents in Kuwait. We used a measurement method that is recommended for epidemiological studies and gathered data from both parents and adolescents. However, we did not measure the skin color, which can be an important determinant for vitamin D formulation in skin during exposure to sunlight. Furthermore, there were adolescents with low vitamin D concentration which was not accompanied by secondary hyperparathyroidism (Figure 1). This has been noted in other studies [28, 36], the clinical significance of which is unknown in children and deserves further study. We evaluated the dietary intake of vitamin D in only a subgroup of study participants. However, dietary intake of vitamin D is known to have a smaller contribution to vitamin D status compared to cutaneous synthesis in response to sun exposure [38] and data on vitamin D supplement were obtained from all participants.

Conclusion

In conclusion, a high prevalence of vitamin D deficiency was noted among adolescents in Kuwait despite the abundant sunshine. This may reflect strong sun avoidance behavior as only less than 4% of adolescents were vitamin D sufficient and most of them were on vitamin D supplements. An optimal vitamin D level is essential during adolescence; and vitamin D deficiency at this age represents a public health problem that should be addressed. First, our findings should help clear doubts on vitamin D deficiency among adolescents in Kuwait, which is a prerequisite for prevention initiatives. Second, sun exposure is the main source for vitamin D in this age group; therefore adequate outdoor daytime activities should be encouraged especially for females. Slight modifications in school environment to facilitate exposure to sunlight during school breaks between classes can be fruitful. With respect to vitamin D supplementation, we call for locally

For peer review only - http://bmjopen.bmj.com/site/about/guidelines.xhtml

tailored guidelines in which females should have a higher amount of vitamin D supplement compared to males. Vitamin D fortification particularly for food products that are popular for adolescents in our setting should also be considered. Increasing exposure to sunlight and vitamin D food fortification should not be considered mutually exclusive and both strategies can be adopted.

Acknowledgements

The authors acknowledge the assistance of Nadien Sameeh Rushdi and the team of data collectors. We also acknowledge the cooperation of all participating schools and the facilitation of the project by the Ministry of Education. We also appreciate the support and cooperation of the staff and management of The United Genetics Laboratories (Kuwait) in vitamin D analysis.

Authors' contributions

AT contributed to the study design and data collection, analyzed the data and drafted the paper. He had full access to all the data and took responsibility for the integrity of the data. AR contributed to the design of the study and data collection in addition to the writing and revising the manuscript. RS and LS contributed to the design of the study and data collection in addition to revising the manuscript with significant intellectual input. AH contributed to the data collection and revised the manuscript.

Funding: The study was supported and funded by Kuwait University, Research Sector (Project No. WF 02/13).

Competing interests: The authors declare that they have no competing interests

Data sharing statement: No additional data are available.

For peer review only - http://bmjopen.bmj.com/site/about/guidelines.xhtml

References

1 Mouratidou T, Vicente-Rodriguez G, Gracia-Marco L*, et al.* Associations of dietary calcium, vitamin D, milk intakes, and 25-hydroxyvitamin D with bone mass in Spanish adolescents: the HELENA study. *Journal of clinical densitometry : the official journal of the International Society for Clinical Densitometry* 2013;**16**:110-7.

2 Sharief S, Jariwala S, Kumar J*, et al.* Vitamin D levels and food and environmental allergies in the United States: results from the National Health and Nutrition Examination Survey 2005-2006. *The Journal of allergy and clinical immunology* 2011;**127**:1195-202.

3 Lucato P, Solmi M, Maggi S*, et al.* Low vitamin D levels increase the risk of type 2 diabetes in older adults: A systematic review and meta-analysis. *Maturitas* 2017;**100**:8-15.

4 Bahrami A, Mazloum SR, Maghsoudi S*, et al.* High Dose Vitamin D Supplementation Is Associated With a Reduction in Depression Score Among Adolescent Girls: A Nine-Week Follow-Up Study. *Journal of dietary supplements* 2017:1-10.

5 Chowdhury R, Kunutsor S, Vitezova A*, et al.* Vitamin D and risk of cause specific death: systematic review and meta-analysis of observational cohort and randomised intervention studies. *BMJ (Clinical research ed)* 2014;**348**:g1903.

6 Hilger J, Friedel A, Herr R*, et al.* A systematic review of vitamin D status in populations worldwide. *The British journal of nutrition* 2014;**111**:23-45.

7 Basatemur E, Horsfall L, Marston L*, et al.* Trends in the Diagnosis of Vitamin D Deficiency. *Pediatrics* 2017;**139**.

8 Garg MK, Marwaha RK, Khadgawat R*, et al.* Efficacy of vitamin D loading doses on serum 25-hydroxy vitamin D levels in school going adolescents: an open label non-randomized prospective trial. *Journal of pediatric endocrinology & metabolism : JPEM* 2013;**26**:515-23.

9 Kapil U, Pandey RM, Goswami R*, et al.* Prevalence of Vitamin D deficiency and associated risk factors among children residing at high altitude in Shimla district, Himachal Pradesh, India. *Indian journal of endocrinology and metabolism* 2017;**21**:178-83.

10 AlBuhairan FS, Tamim H, Al Dubayee M*, et al.* Time for an Adolescent Health Surveillance System in Saudi Arabia: Findings From "Jeeluna". *The Journal of adolescent health : official publication of the Society for Adolescent Medicine* 2015;**57**:263-9.

11 Kaddam IM, Al-Shaikh AM, Abaalkhail BA*, et al.* Prevalence of vitamin D deficiency and its associated factors in three regions of Saudi Arabia. *Saudi medical journal* 2017;**38**:381-90.

12 Mansour MM, Alhadidi KM. Vitamin D deficiency in children living in Jeddah, Saudi Arabia. *Indian journal of endocrinology and metabolism* 2012;**16**:263-9.

13 Valtuena J, Gonzalez-Gross M, Huybrechts I*, et al.* Factors associated with vitamin D deficiency in European adolescents: the HELENA study. *Journal of nutritional science and vitaminology* 2013;**59**:161-71.

14 Glanz K, Yaroch AL, Dancel M*, et al.* Measures of sun exposure and sun protection practices for behavioral and epidemiologic research. *Archives of dermatology* 2008;**144**:217-22.

15 UEA. Youth Physical Activity Questionnaire. University of East Anglia 2011.

For peer review only - http://bmjopen.bmj.com/site/about/guidelines.xhtml

16 Al-Hazzaa HM, Al-Sobayel HI, Musaiger AO. Convergent validity of the Arab Teens Lifestyle Study (ATLS) physical activity questionnaire. *International journal of environmental research and public health* 2011;**8**:3810-20.

17 Taylor C, Lamparello B, Kruczek K*, et al.* Validation of a food frequency questionnaire for determining calcium and vitamin D intake by adolescent girls with anorexia nervosa. *Journal of the American Dietetic Association* 2009;**109**:479-85, 85 e1-3.

18 Papandreou D, achaniotis N, Lari M*, et al.* Validation of a Food Frequency Questionnaire for Vitamin D and Calcium Intake in Healthy Female College Students. *Food and Nutrition Sciences* 2014;**5**:2048-52.

19 Holick MF. Vitamin D deficiency. *The New England journal of medicine* 2007;**357**:266-81.

20 Vogeser M. Quantification of circulating 25-hydroxyvitamin D by liquid chromatography-tandem mass spectrometry. *The Journal of steroid biochemistry and molecular biology* 2010;**121**:565-73.

21 Holick MF, Binkley NC, Bischoff-Ferrari HA*, et al.* Evaluation, treatment, and prevention of vitamin D deficiency: an Endocrine Society clinical practice guideline. *The Journal of clinical endocrinology and metabolism* 2011;**96**:1911-30.

22 Medicine SfAHa. Recommended vitamin D intake and management of low vitamin D status in adolescents: a position statement of the society for adolescent health and medicine. *The Journal of adolescent health : official publication of the Society for Adolescent Medicine* 2013;**52**:801-3.

23 Braegger C, Campoy C, Colomb V*, et al.* Vitamin D in the healthy European paediatric population. *Journal of pediatric gastroenterology and nutrition* 2013;**56**:692-701.

24 Vierucci F, Del Pistoia M, Fanos M*, et al.* Prevalence of hypovitaminosis D and predictors of vitamin D status in Italian healthy adolescents. *Italian journal of pediatrics* 2014;**40**:54.

25 Gonzalez-Gross M, Valtuena J, Breidenassel C*, et al.* Vitamin D status among adolescents in Europe: the Healthy Lifestyle in Europe by Nutrition in Adolescence study. *The British journal of nutrition* 2012;**107**:755-64.

26 Vierucci F, Del Pistoia M, Fanos M*, et al.* Vitamin D status and predictors of hypovitaminosis D in Italian children and adolescents: a cross-sectional study. *European journal of pediatrics* 2013;**172**:1607-17.

27 Kassi EN, Stavropoulos S, Kokkoris P*, et al.* Smoking is a significant determinant of low serum vitamin D in young and middle-aged healthy males. *Hormones (Athens, Greece)* 2015;**14**:245-50.

28 Gordon CM, DePeter KC, Feldman HA*, et al.* Prevalence of vitamin D deficiency among healthy adolescents. *Archives of pediatrics & adolescent medicine* 2004;**158**:531-7.

29 Dong Y, Pollock N, Stallmann-Jorgensen IS*, et al.* Low 25-hydroxyvitamin D levels in adolescents: race, season, adiposity, physical activity, and fitness. *Pediatrics* 2010;**125**:1104-11.

30 Rockell JE, Green TJ, Skeaff CM*, et al.* Season and ethnicity are determinants of serum 25-hydroxyvitamin D concentrations in New Zealand children aged 5-14 y. *The Journal of nutrition* 2005;**135**:2602-8.

31 Byun EJ, Heo J, Cho SH*, et al.* Suboptimal vitamin D status in Korean adolescents: a nationwide study on its prevalence, risk factors including cotinine-verified smoking status and association with atopic dermatitis and asthma. *BMJ open* 2017;**7**:e016409.

32 Zhang FF, Al Hooti S, Al Zenki S*, et al.* Vitamin D deficiency is associated with high prevalence of diabetes in Kuwaiti adults: results from a national survey. *BMC public health* 2016;**16**:100.

For peer review only - http://bmjopen.bmj.com/site/about/guidelines.xhtml

33 Al-Daghri NM. Vitamin D in Saudi Arabia: Prevalence,distribution and disease associations. *The Journal of steroid biochemistry and molecular biology* 2016.

34 Golbahar J, Al-Saffar N, Altayab Diab D*, et al.* Predictors of vitamin D deficiency and insufficiency in adult Bahrainis: a cross-sectional study. *Public health nutrition* 2014;**17**:732-8.

35 Mahdy S, Al-Emadi SA, Khanjar IA*, et al.* Vitamin D status in health care professionals in Qatar. *Saudi medical journal* 2010;**31**:74-7.

36 Sulimani RA, Mohammed AG, Alfadda AA*, et al.* Vitamin D deficiency and biochemical variations among urban Saudi adolescent girls according to season. *Saudi medical journal* 2016;**37**:1002-8.

37 Thalib L, Al-Taiar A. Dust storms and the risk of asthma admissions to hospitals in Kuwait. *The Science of the total environment* 2012;**433**:347-51.

38 Holick MF. The D-lightful vitamin D for child health. *JPEN Journal of parenteral and enteral nutrition* 2012;**36**:9S-19S.

39 Al-Daghri NM, Al-Saleh Y, Aljohani N*, et al.* Vitamin D Deficiency and Cardiometabolic Risks: A Juxtaposition of Arab Adolescents and Adults. *PloS one* 2015;**10**:e0131315.

40 Yao TC, Tu YL, Chang SW*, et al.* Suboptimal vitamin D status in a population-based study of Asian children: prevalence and relation to allergic diseases and atopy. *PloS one* 2014;**9**:e99105.

41 Wortsman J, Matsuoka LY, Chen TC*, et al.* Decreased bioavailability of vitamin D in obesity. *The American journal of clinical nutrition* 2000;**72**:690-3.

42 Walker GE, Ricotti R, Roccio M*, et al.* Pediatric obesity and vitamin D deficiency: a proteomic approach identifies multimeric adiponectin as a key link between these conditions. *PloS one* 2014;**9**:e83685.

43 Tsuji K, Maeda T, Kawane T*, et al.* Leptin stimulates fibroblast growth factor 23 expression in bone and suppresses renal 1alpha,25-dihydroxyvitamin D3 synthesis in leptin-deficient mice. *J Bone Miner Res* 2010;**25**:1711-23.

44 O'Connor TM, Yang SJ, Nicklas TA. Beverage intake among preschool children and its effect on weight status. *Pediatrics* 2006;**118**:e1010-8.

45 Scragg R, Holdaway I, Jackson R*, et al.* Plasma 25-hydroxyvitamin D3 and its relation to physical activity and other heart disease risk factors in the general population. *Annals of epidemiology* 1992;**2**:697-703.

46 Aboraia AS, Makowski B, Bahja A*, et al.* Synthesis and CYP24A1 inhibitory activity of (E)-2-(2-substituted benzylidene)- and 2-(2-substituted benzyl)-6-methoxy-tetralones. *European journal of medicinal chemistry* 2010;**45**:4427-34.

For peer review only - http://bmjopen.bmj.com/site/about/guidelines.xhtml

Figure legends

Figure (1): Parathyroid hormone (PTH) with Serum 25-hydroxyvitamin D (25-OH-D) concentration. Individuals above the horizontal line (PTH ≥ 65.0 ng/L) are adolescents with secondary hyperthyroidism. Vertical lines represent the 25-OH-D cut-off used to define vitamin D deficiency, insufficiency and sufficiency (seven participants with 25-OH-D>150 nmol/L were omitted).

For peer review only

For peer review only - http://bmjopen.bmj.com/site/about/guidelines.xhtml

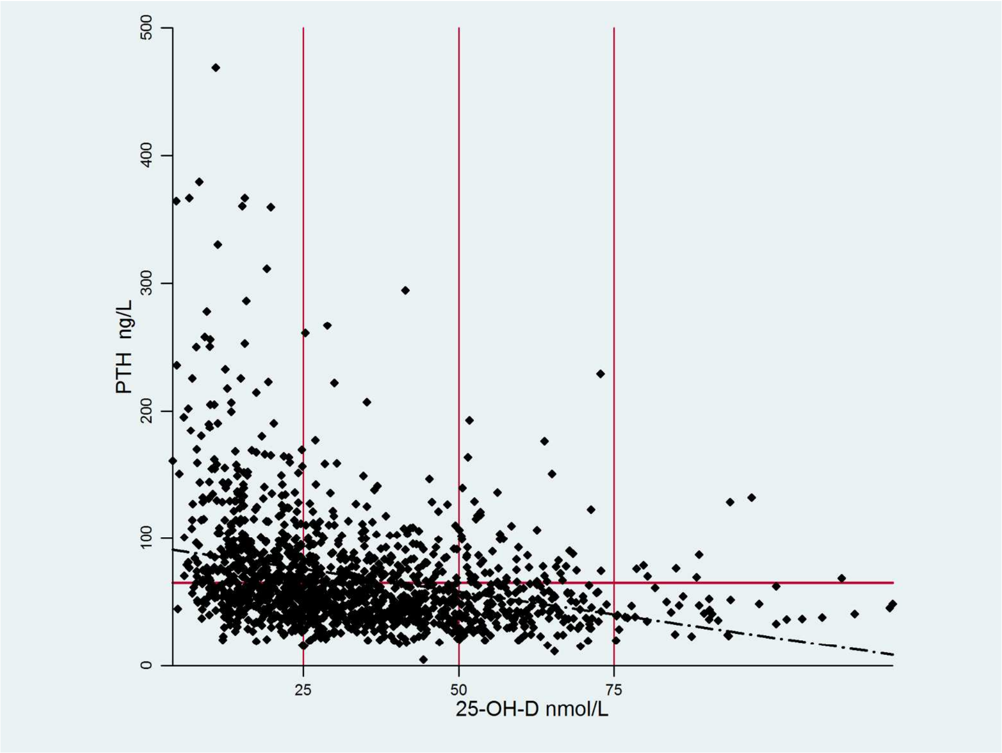

Figure (1): Parathyroid hormone (PTH) with Serum 25-hydroxyvitamin D (25-OH-D) concentration. Individuals above the horizontal line (PTH ≥ 65.0 ng/L) are adolescents with secondary hyperthyroidism. Vertical lines represent the 25-OH-D cut-off used to define vitamin D deficiency, insufficiency and sufficiency (seven participants with 25-OH-D>150 nmol/L were omitted).

705x529mm (72 x 72 DPI)

Supplementary Table (1). Factors associated with vitamin D deficiency in univariate analysis, 1416 middle school students.

Characteristics	Prevalence of vitamin D deficiency n (%)	Prevalence Ratio [95%CI]	p
**Gender**			
Male	488 (70.32)	1 [Ref.]	<0.001
Female	662 (91.69)	1.30 [1.26- 1.33]	
**Age (year)**			
<12	409 (77.61)	1 [Ref.]	0.024
12-	369 (84.05)	1.07 [1.01- 1.12]	
≥13	372 (82.67)	1.06 [1.00- 1.10]	
**Nationality**			
Kuwaiti	900 (83.26)	1 [Ref.]	<0.001
Non-Kuwait	250 (74.63)	0.90 [0.82-0.96]	
**Governorate**[1]			
Capital	127 (81.41)	1 [Ref.]	0.003
Hawally	188 (76.42)	0.94 [0.82-1.03]	
Farawanya	183 (77.54)	0.95 [0.83-1.04]	
Jahra	214 (89.54)	1.10 [1.02-1.15]	
Mubarak al-Kabeer	124 (83.78)	1.03 [0.91-1.10]	
Ahmadi	296 (79.78)	0.98 [0.88-1.06]	
**Father's Education**[2]			
Primary/Intermediate/no formal education	205 (86.86)	1 [Ref.]	<0.001
Secondary (high school)	297 (86.34)	0.99 [0.87-1.08]	
Diploma	220 (84.29)	0.95 [0.82-1.06]	
University & above	399 (73.62)	0.78 [0.65-0.90]	
**Mother's Education**[3]			
Primary/Intermediate/no formal education	153 (83.61)	1 [Ref.]	<0.001
Secondary (high school)	263 (86.51)	1.04 [0.94-1.12]	
Diploma	259 (85.20)	1.02 [0.91-1.10]	
University & above	457 (75.54)	0.88 [0.76-0.99]	
**Total number of brother/sisters**[4]			
Zero-two	223 (74.83)	1 [Ref.]	0.002
Three-four	434 (80.67)	1.05 [1.00-1.10]	
Five or more	469 (84.81)	1.10 [1.04-1.13]	
**Passive smoking at household**[5]			
No	704 (78.14)	1 [Ref.]	<0.001
Yes	426 (87.12)	1.11 [1.06-1.15]	

**Times per week consumed breakfast not prepared at home**					
Zero	484	(77.56)	1	[Ref.]	0.019
One-two times	478	(84.01)	1.08	[1.02-1.12]	
Three-four	98	(83.76)	1.07	[0.97-1.14]	
Five or more	67	(85.90)	1.10	[1.00-1.17]	
**Times per week consumed dinner not prepared at home**					
Zero	114	(72.61)	1	[Ref.]	0.018
One-two times	719	(81.89)	1.09	[1.03-1.14]	
Three-four	211	(82.10)	1.09	[1.01-1.15]	
Five or more	75	(87.21)	1.14	[1.04-1.19]	
**Consumption of sugary drinks per week**					
None	123	(71.93)	1	[Ref.]	0.002
Once-Three times	633	(81.99)	1.09	[1.04-1.14]	
Four-Six	135	(78.95)	1.07	[0.98-1.13]	
Seven/more	256	(85.62)	1.12	[1.07-1.17]	
**Currently taking supplements**[5]					
No	1,045	(83.20)	1	[Ref.]	<0.001
Yes	103	(65.19)	0.78	[0.68-0.88]	
**Number of times walking to school per week**[5]					
None	982	(84.80)	1	[Ref.]	<0.001
1-8 times	99	(63.87)	0.74	[0.63-0.83]	
Every day	69	(66.99)	0.77	[0.65-0.89]	
**Time walking to school**[5]					
None	982	(84.80)	1	[Ref.]	<0.001
≤5 minutes	50	(62.50)	0.71	[0.56-0.84]	
6- 10 minutes	53	(69.74)	0.80	[0.65-0.93]	
11-15	38	(67.86)	0.77	[0.60-0.92]	
16 minutes or more	26	(57.78)	0.65	[0.46-0.82]	
**Time spent on physical activity per week**					
Low	396	(84.43)	1	[Ref.]	0.005
Medium	391	(82.66)	0.97	[0.89-1.04]	
High	363	(76.58)	0.89	[0.80-0.96]	
**Body Mass Index Categories**[6]					
Normal weight	465	(77.37)	1	[Ref.]	0.001
Overweight	270	(84.38)	1.07	[1.02-1.11]	
Obese	400	(84.93)	1.08	[1.03-1.11]	
Under weight	15	(62.50)	0.84	[0.59-1.02]	

**Time outside per day 10:00 am- 4:00 pm week days**					
Less than 30 minutes	563	(85.69)	1	[Ref.]	<0.001
31 minutes to 1 hour	342	(76.00)	0.87	[0.80-0.94]	
More than 1 hour to 2 hours	178	(80.18)	0.93	[0.84-1.00]	
More than 2 hours	67	(77.01)	0.88	[0.74-0.99]	
**Time outside per day 10:00 am- 4:00 pm week ends**					
Less than 30 minutes	425	(86.03)	1	[Ref.]	0.003
31 minutes to 1 hour	257	(82.37)	0.94	[0.85-1.02]	
More than 1 hour to 2 hours	198	(77.65)	0.87	[0.76-0.96]	
More than 2 hours to 3 hours	142	(76.76)	0.85	[0.74-0.96]	
More than 3 hours	127	(75.15)	0.83	[0.71-0.94]	
**Wearing sunscreen**					
Never	874	(79.45)	1	[Ref.]	0.013
Rarely	65	(84.42)	1.06	[0.94-1.13]	
Sometimes	140	(87.50)	1.09	[1.02-1.14]	
Often/always	71	(89.87)	1.12	[1.02-1.18]	
**Staying in shade or under an umbrella**					
Never	396	(80.32)	1	[Ref.]	0.04
Rarely	70	(72.16)	0.90	[0.78-1.01]	
Sometimes	301	(82.92)	1.03	[0.96-1.08]	
Often	162	(79.02)	0.98	[0.90-1.05]	
Always	220	(85.60)	1.06	[0.99-1.11]	

[1]Missing for 20 participants; [2] Missing for 33 participants; [3] Missing for 20 participants; [4]Missing for 27 participants; [5] Missing for 2 participants; [6]Missing for 26 participants; [7] according to WHO growth charts.

only

STROBE 2007 (v4) checklist of items to be included in reports of observational studies in epidemiology*
Checklist for cohort, case-control, and cross-sectional studies (combined)

Section/Topic	Item #	Recommendation	Reported on page #
**Title and abstract**	1	(*a*) Indicate the study's design with a commonly used term in the title or the abstract	Page 1
		(*b*) Provide in the abstract an informative and balanced summary of what was done and what was found	Page 3
**Introduction**			
Background/rationale	2	Explain the scientific background and rationale for the investigation being reported	Page 5
Objectives	3	State specific objectives, including any pre-specified hypotheses	Page 5
**Methods**			
Study design	4	Present key elements of study design early in the paper	Page 6
Setting	5	Describe the setting, locations, and relevant dates, including periods of recruitment, exposure, follow-up, and data collection	Page 6- Page 8 and Page 14
Participants	6	(*a*) *Cohort study*—Give the eligibility criteria, and the sources and methods of selection of participants. Describe methods of follow-up<br>*Case-control study*—Give the eligibility criteria, and the sources and methods of case ascertainment and control selection. Give the rationale for the choice of cases and controls<br>*Cross-sectional study*—Give the eligibility criteria, and the sources and methods of selection of participants	Page 6
		(*b*) *Cohort study*—For matched studies, give matching criteria and number of exposed and unexposed<br>*Case-control study*—For matched studies, give matching criteria and the number of controls per case	
Variables	7	Clearly define all outcomes, exposures, predictors, potential confounders, and effect modifiers. Give diagnostic criteria, if applicable	Page 6 and Page 7
Data sources/ measurement	8*	For each variable of interest, give sources of data and details of methods of assessment (measurement). Describe comparability of assessment methods if there is more than one group	*Page 6 and Page 7*
Bias	9	Describe any efforts to address potential sources of bias	Page 6, Page 12 and Page 17
Study size	10	Explain how the study size was arrived at	Page 13
Quantitative variables	11	Explain how quantitative variables were handled in the analyses. If applicable, describe which groupings were chosen and why	Page 7 and Page 8
Statistical methods	12	(*a*) Describe all statistical methods, including those used to control for confounding	Page 7 and Page 8
		(*b*) Describe any methods used to examine subgroups and interactions	Page 7 and Page 8
		(*c*) Explain how missing data were addressed	These were minimum.

For peer review only

		(*d*) *Cohort study*—If applicable, explain how loss to follow-up was addressed<br>*Case-control study*—If applicable, explain how matching of cases and controls was addressed<br>*Cross-sectional study*—If applicable, describe analytical methods taking account of sampling strategy	Page 8
		(*e*) Describe any sensitivity analyses	
**Results**			
Participants	13*	(a) Report numbers of individuals at each stage of study—eg numbers potentially eligible, examined for eligibility, confirmed eligible, included in the study, completing follow-up, and analysed	Page 8
		(b) Give reasons for non-participation at each stage	Page 8
		(c) Consider use of a flow diagram	
Descriptive data	14*	(a) Give characteristics of study participants (eg demographic, clinical, social) and information on exposures and potential confounders	Table 1 page 9
		(b) Indicate number of participants with missing data for each variable of interest	Table 1 , Table 2 and also supplementary table
		(c) *Cohort study*—Summarise follow-up time (eg, average and total amount)	
Outcome data	15*	*Cohort study*—Report numbers of outcome events or summary measures over time	
		Case-control study—Report numbers in each exposure category, or summary measures of exposure	
		Cross-sectional study—Report numbers of outcome events or summary measures	Page 10
Main results	16	(*a*) Give unadjusted estimates and, if applicable, confounder-adjusted estimates and their precision (eg, 95% confidence interval). Make clear which confounders were adjusted for and why they were included	
		(*b*) Report category boundaries when continuous variables were categorized	All tables
		(*c*) If relevant, consider translating estimates of relative risk into absolute risk for a meaningful time period	
Other analyses	17	Report other analyses done—eg analyses of subgroups and interactions, and sensitivity analyses	Page 12
**Discussion**			
Key results	18	Summarise key results with reference to study objectives	Page 13
Limitations	19	Discuss limitations of the study, taking into account sources of potential bias or imprecision. Discuss both direction and magnitude of any potential bias	Page 16
Interpretation	20	Give a cautious overall interpretation of results considering objectives, limitations, multiplicity of analyses, results from similar studies, and other relevant evidence	Page 17
Generalisability	21	Discuss the generalisability (external validity) of the study results	Page 17
**Other information**			
Funding	22	Give the source of funding and the role of the funders for the present study and, if applicable, for the original study on which the present article is based	Page 18

For peer review only

*Give information separately for cases and controls in case-control studies and, if applicable, for exposed and unexposed groups in cohort and cross-sectional studies.
Note: An Explanation and Elaboration article discusses each checklist item and gives methodological background and published examples of transparent reporting. The STROBE checklist is best used in conjunction with this article (freely available on the Web sites of PLoS Medicine at http://www.plosmedicine.org/, Annals of Internal Medicine at http://www.annals.org/, and Epidemiology at http://www.epidem.com/). Information on the STROBE Initiative is available at www.strobe-statement.org.

For peer review only

BMJ Open

Vitamin D Status among Adolescents in Kuwait: A Cross-sectional Study.

Journal:	*BMJ Open*
Manuscript ID	bmjopen-2017-021401.R2
Article Type:	Research
Date Submitted by the Author:	01-May-2018
Complete List of Authors:	Al-Taiar, Abdullah; Faculty of Medicine, Kuwait University, Department of Community Medicine and Behavioural Sciences<br>Rahman , Abdur ; College of Life Sciences, Kuwait University, Department of Food Science and Nutrition<br>Al-Sabah, Reem; Faculty of Medicine, Kuwait University, Department of Community Medicine and Behavioural Sciences<br>Shaban, Lemia ; College of Life Sciences, Kuwait University, Department of Food Science and Nutrition<br>Al-Harbi, Anwar ; Kuwait Institute for Scientific Research , Department of Science and Nutrition
**Primary Subject Heading**:	Nutrition and metabolism
Secondary Subject Heading:	Epidemiology, Global health, Paediatrics, Public health
Keywords:	PUBLIC HEALTH, Vitamin D, Adolescents, school children, Kuwait

SCHOLARONE™ Manuscripts

For peer review only

Vitamin D Status among Adolescents in Kuwait: A Cross-sectional Study.

Abdullah Al-Taiar[1], Abdur Rahman[2], Reem Al-Sabah[1], Lemia Shaban[2], Anwar Al-Harbi[3]

[1] Department of Community Medicine and Behavioural Sciences

Faculty of Medicine, Kuwait University,

Box: 24923 Safat-13110 Kuwait.

[2] Department of Food Science and Nutrition

College of Life Sciences, Kuwait University,

Box 5969, Safat-13060, Kuwait.

[3] Kuwait Institute for Scientific Research,

Department of Science and Nutrition,

Box: 24885 Safat-13109, Kuwait.

Before publication corresponding author:

Dr Abdullah Al-Taiar

Associate Professor of Epidemiology

Department Community Medicine and Behavioural Sciences,

Faculty of Medicine, Kuwait University,

Box: 24923 Safat-13110 Kuwait.

Tel: +965-24986553

Fax: +965-5338948

E-mail: altaiar@hsc.edu.kw

For peer review only

For peer review only - http://bmjopen.bmj.com/site/about/guidelines.xhtml

<u>After publication</u> corresponding author:

Abdur Rahman

Associate Professor

Department of Food Science and Nutrition,

College of Life Sciences, Kuwait University,

Box: 5969 Safat-13060 Kuwait.

Tel: +965-249633055

Fax: +965-22513929

E-mail: abdurrahman.ahmad@ku.edu.kw

For peer review only

For peer review only - http://bmjopen.bmj.com/site/about/guidelines.xhtml

Abstract

Objectives

In Kuwait, as in many Arab states in the gulf region, there are limited data on the prevalence of vitamin D deficiency among healthy adolescents. This study aimed to estimate the prevalence of vitamin D deficiency in a nationally representative sample of adolescents and investigate factors associated with vitamin D status.

Methods

A cross-sectional study was conducted on 1416 adolescents aged 11-16 years, who were randomly selected from middle schools in all governorates of Kuwait. Data were collected from parents through self-administered questionnaire and from adolescents through face-to-face interview. Vitamin D was measured using liquid chromatography-tandem mass spectrometry (LC-MS/MS). Logistic regression was used to investigate the independent factors associated with vitamin D status.

Results

The Prevalence of vitamin D deficiency was 1,150 (81.21%, 95%CI: 71.61-90.81%) while severe deficiency was 559 (39.48%). Only 51 (3.60%) were vitamin D sufficient. Prevalence of vitamin D deficiency was significantly higher among females compared to males (91.69% vs. 70.32%; p<0.001). There was a significant inverse correlation between vitamin D and PTH (Spearman correlation=-0.35; p<0.001). In the final model, gender, age, governorate, parental education, body mass index, vitamin D supplement and number of times walking to school per week were all significantly related to vitamin D deficiency.

Conclusion

High prevalence of vitamin D deficiency was noted among adolescents in Kuwait despite the abundant sunshine which may reflect strong sun avoidance behavior. Adequate outdoor daytime activities should be encouraged especially for females. We call for locally tailored guidelines for vitamin D supplement in which females should have a higher dose compared to males.

Keywords: Vitamin D, adolescents, Kuwait, Middle East, school children

For peer review only - http://bmjopen.bmj.com/site/about/guidelines.xhtml

Strengths and limitations of this study

- This is the first study that aimed to estimate the prevalence of vitamin D on a large nationally representative sample of adolescents in Kuwait.
- We measured vitamin D using the recommended laboratory methodology and gathered data from both parents and adolescents.
- We did not measure the skin color, which can be an important determinant for vitamin D synthesis in the skin.

For peer review only

For peer review only - http://bmjopen.bmj.com/site/about/guidelines.xhtml

Introduction

An optimal vitamin D status is extremely important during adolescence for proper growth and bone mineral accrual [1]. In addition to its critical role in bone health, several studies have linked vitamin D deficiency with various disease conditions during adolescence or adulthood such as asthma and allergies [2], type 2 diabetes [3], depression[4], cancer and even all-cause mortality [5]. However, the results of randomized control trials, which tested the impact of vitamin D supplement on the risk of these disease conditions, remain inconclusive.

Several studies have evaluated the prevalence of vitamin D deficiency and insufficiency during adolescence reporting high prevalence of vitamin D deficiency worldwide [6 7]. This was the case even in countries with abundant sunshine, which is the main factor for endogenous vitamin D synthesis. For example, in India the prevalence of vitamin D deficiency among adolescents was reported to be 85-98% [8 9] while in Saudi Arabia it was found to be around 96% [10]. In Arab states in the Gulf region or the broader Middle East, there is paucity of data on vitamin D status in pediatric and adolescents populations. The few studies that showed a high prevalence of vitamin D deficiency in adolescents suffered from major methodological weaknesses [10-12]. They were either small hospital-based, lacked proper sampling technique or used less optimal laboratory method to measure vitamin D. It is also known that factors which influence vitamin D status such as avoiding sun exposure, skin pigmentation, high body mass index (BMI) and dietary factors, particularly the lack of vitamin D supplement and intake of vitamin D rich foods [13] are all on rise in Arab states in the Gulf region. The aim of the present study was therefore to assess vitamin D status among adolescents living in Kuwait, a country with a plenty of sun shine, and to investigate factors associated with low vitamin D level.

For peer review only - http://bmjopen.bmj.com/site/about/guidelines.xhtml

Methods

Study population and study participants

Kuwait is a small country at latitude (29.3759° N) with a population of 4.2 million. Approximately, 25% of population is under the age of 19-year. The study population comprised students in the age between 11 and 16 years in the public middle schools from all governorates of Kuwait. A school based cross-sectional study was conducted on students from 12 public middle schools that were selected using a stratified multistage cluster random sampling with a probability proportional to size. The sample allocation in each governorate was based on the relative size of that governorate as judged by the total number of students in the governorate. Students with major chronic disease conditions are registered in each school and these were excluded from the study. However, students with minor illnesses or self-reported illness were included in the study. The type of illness was recorded during data collection and included in the analysis. The study was funded by Kuwait University and approved by The Ethics Committee at The Ministry of Health in Kuwait as well as The Ethics Committee at Health Sciences Centre, Kuwait University.

Data Collection

An informed consent was sent to the parents along with a self-administered questionnaire on parents' level of education (No formal education, primary/intermediate, high school, diploma, university and above), income, type of housing, number of siblings of the index child, passive smoking at household and the number of times per week the index child had a meal prepared outside home during the last three months. After obtaining written informed consent from the parents and verbal consent from the school children, trained dedicated personnel carried out face-to-face interviews with the students using a structured questionnaire. The questionnaire was carefully developed after extensive review of the literature and was pilot tested on 20 students who were not included in the study. It comprised questions on habitual sun exposure during the last three months, which was assessed by the core questions developed to measure

For peer review only - http://bmjopen.bmj.com/site/about/guidelines.xhtml

the exposure to sunlight in adolescents as described by Glanz et al. [14]; in addition to smoking habits, physical activity and dietary intake. Data on physical activity were collected using a questionnaire that was developed based on the Youth Physical Activity Questionnaire in UK [15] and The Arab teens lifestyle study [16]. The questionnaire was validated among high school students and showed strong correlation with data collected by accelerometers (Spearman correlation= 0.92; p<0.001 for total steps count) (not published). Data on dietary intake of vitamin D were collected from only 200 students using food frequency questionnaire for calcium and vitamin D intake in adolescents [17], which has been validated in our setting [18]. Food models or serving containers were used to assist in estimating serving size. Measurements of standing height and body weight of the study subjects were assessed using a digital weight and height scale (Detecto®) in a standardized manner.

Laboratory methods

Five ml of venous blood was collected in gel-containing tubes (SST II advance, BD vacutainer) from each participant by a trained nurse; and the samples were protected from light. On the same day, the samples were centrifuged at 2000 x g for 15 minutes and the serum was transferred to Eppendorf tubes and stored at -80°C until analysis. Serum 25-hydroxyvitamin D (25-OH-D) concentration is the best marker of vitamin D status because it is the major circulating form of vitamin D, reflecting both the amount produced in the skin after sun exposure and the amount consumed in foods [19]. It was measured in College of American Pathologists CAP-accredited laboratory by liquid chromatography-tandem mass spectrometry (LC-MS/MS), which is the recommended method for vitamin D assessment in epidemiological studies [20]. According to the Endocrine Society [21] and the Society for Adolescent Health and Medicine [22], we used the following cut-off of 25-OH-D to define vitamin D status: vitamin D deficiency < 50 nmol/L (20 ng/mL); vitamin D insufficiency 50-75 nmol/L (20-30 ng/mL); and vitamin D sufficiency≥ 75 nmol/L (30 ng/mL). Thus, hypovitaminosis D was defined in the presence of 25-OH-D levels < 75.0 nmol/L (30 ng/mL). Furthermore, severe vitamin D deficiency was defined as 25-OH-D levels < 25.0 nmol/L (10 ng/mL) [23]. Serum intact

For peer review only - http://bmjopen.bmj.com/site/about/guidelines.xhtml

Parathyroid hormone (PTH) was measured using the Access Intact PTH chemiluminescent immunoassay with the Unicel DxI 800 Beckman Coulter analyzer using commercial kit (Cat. # A16972). Similar to other studies, PTH level ≥ 65.0 ng/L was suggestive of hyperparathyroidism [24] although there is no consensus on this cut-off point.

Patient and Public Involvement

Public were not directly involved in the design of this study. However, schools were involved in approaching the parents to obtain their consents. We also sent the results of the laboratory tests of each participant to the parents through schools using closed envelops to ensure confidentiality.

Data analysis

Data were double entered into specifically designed database using Epidata Entry. Data analysis was conducted using Stata (StataCorp. 2011. Release 12). BMI was calculated as weight (Kg) divided by height squared (m^2). Weight status was categorized into normal, overweight and obese according to WHO growth charts. Chi-squared test was used to test differences in categorical variables. Because vitamin D deficiency was a common outcome and the log-binomial model failed to converge, a modified version of multiple logistic regression that calculates prevalence ratio was used to study the association between each presumed risk factor and vitamin D deficiency (25-OH-D < 50 nmol/L). Variables that were found to be statistically significant at 15% level of significance were considered in multivariate analysis. Variables were divided into groups (e.g. socio-demographic, sun exposure, etc) and were added sequentially to the model. Statistical significance of variables was evaluated by likelihood ratio test which compares the model with and without the variable. Goodness of fit of the final model was evaluated by Hosmer-Lemeshow test. Factors with $p<0.05$ were deemed to be statistically significant. The analysis above was repeated using hypovitaminosis (25-OH-D levels < 75.0 nmol/L) as the binary outcome. We also conducted multiple linear regression analysis using vitamin D level as a continuous outcome (after log-transformation) and reported

For peer review only - http://bmjopen.bmj.com/site/about/guidelines.xhtml

the results in the text. Because of the complex structure of this survey data, we used survey method, which gives more precise estimates of standard error.

Results

Of 1583 parents approached, 161 refused to participate (because children, parents or both refused). Another 6 samples were not sufficient to conduct blood analysis, thus the analysis below comprised 1416 students. Table (1) shows the socio-demographic characteristics of the study group. The mean (SD) age was 12.48 (0.94) years and 694 (49.01%) were males.

Table (1). Socio-demographic characteristics and vitamin D level among adolescents in public middle schools in Kuwait.

Characteristics	Males n=694		Females n=722		Total N=1416	
**Age in years,** Mean (SD) years	12.56	(0.94)	12.41	(0.92)	12.48	0.94
**25-OH-D nmol/L, Median (IQR)**	39.8	(29.4-52.7)	21.5	(14.7-30.7)	29.7	(19.2-44.2)
	**n**	**(%)**	**n**	**(%)**	**n**	**(%)**
**Nationality** (N= 1416)						
Kuwaiti	423	(60.95)	658	(91.14)	1,081	(76.34)
Non-Kuwait	271	(39.05)	64	(8.86)	335	(23.66)
**Father's Education** (N=1383)						
No formal education	9	(1.33)	6	(0.85)	15	(1.08)
Primary/Intermediate	95	(14.05)	126	(17.82)	221	(15.98)
Secondary (high school)	157	(23.22)	187	(26.45)	344	(24.87)
Diploma	126	(18.64)	135	(19.09)	261	(18.87)
University & above	289	(42.75)	253	(35.79)	542	(39.19)
**Mother's Education** (N=1396)						
No formal education	18	(2.64)	13	(1.82)	31	(2.22)
Primary/Intermediate	79	(11.60)	73	(10.21)	152	(10.89)
Secondary (high school)	140	(20.56)	164	(22.94)	304	(21.78)
Diploma	136	(19.97)	168	(23.50)	304	(21.78)
University & above	308	(45.23)	297	(41.54)	605	(43.34)
**Father's Income** (Kuwaiti Dinars) (N=1370)						
Less than 500	64	(9.61)	27	(3.84)	91	(6.64)
500 to 1000	168	(25.23)	136	(19.32)	304	(22.19)
1001 to 1500	175	(26.28)	246	(34.94)	421	(30.73)

For peer review only - http://bmjopen.bmj.com/site/about/guidelines.xhtml

1501 to 2000	100 (15.02)	119 (16.90)	219 (15.99)
More than 2000	83 (12.46)	90 (12.78)	173 (12.63)
Do not wish to tell	76 (11.41)	86 (12.22)	162 (11.82)
**Mother's Employment Status** (N=1388)			
Housewife	267 (39.38)	221 (31.13)	488 (35.16)
Paid employment	307 (45.28)	373 (52.54)	680 (48.99)
Others	104 (15.34)	116 (16.34)	220 (15.85)
**Housing** (N=1397)			
Rented flat	301 (44.20)	209 (29.19)	510 (36.51)
Rented house	94 (13.80)	69 (9.64)	163 (11.67)
Owned flat	17 (2.50)	42 (5.87)	59 (4.22)
Owned house	269 (39.50)	396 (55.31)	665 (47.60)

IQR: Interquartile Range. All %s are column percentages.

For peer review only - http://bmjopen.bmj.com/site/about/guidelines.xhtml

Prevalence of vitamin D deficiency

The median (Interquartile Range, IQR) of 25-OH-D was 29.7 (19.2- 44.2) nmol/L. Prevalence of vitamin D deficiency was 1,150 (81.21%, 95%CI: 71.61-90.81%) while severe deficiency was 559 (39.48%). Only 51 (3.60%) participants were vitamin D sufficient. Prevalence of vitamin D deficiency was significantly higher among females compared to males (91.69% vs. 70.32%; p<0.001). Also, the median (IQR) of vitamin D was 39.8 (29.4- 52.7) nmol/L and 21.5 (14.7- 30.7) nmol/L among males and females, respectively (p<0.001). There was no difference in the prevalence of vitamin D deficiency between Kuwaitis and non-Kuwaitis after stratification by gender. There was significant inverse correlation between vitamin D and PTH (Spearman correlation=-0.35; p<0.001). In relation to vitamin D status, elevated PTH (secondary hyperparathyroidism PTH ≥65 ng/L. i.e. 6.89 pmol/L) was detected in 312 (55.81%), 185 (31.30%) and 59 (27.57%) of adolescents with vitamin D severe deficiency, deficiency and insufficiency, respectively. If we used the reference range of the laboratory where the samples were tested (PTH: 1.3-9.3 pmol/L), the prevalence of secondary hyperparathyroidism would be 182 (32.56%), 73 (12.35%), and 25 (11.68%) among adolescents with vitamin D severe deficiency, deficiency and insufficiency, respectively. The median [IQR] PTH was significantly different dependent on vitamin D status (severe deficiency 7.37 [5.44-10.55] pmol/L; deficiency 5.63[4.13-7.40] pmol/L; insufficiency 5.14[3.87-6.99] pmol/L; sufficiency 4.31[3.75- 5.73] pmol/L; p<0.001). The actual relationship between 25-OH-D and PTH levels is shown in figure (1).

Table (2): Factors associated with vitamin D deficiency among adolescence in multivariate analysis.

Characteristics	Total	Prevalence of vitamin D deficiency n (%)	Prevalence Ratio [95%CI]	P
**Gender**				
Male	694	488 (70.32)	1 [Ref.]	<0.001
Female	722	662 (91.69)	1.20 [1.17- 1.21]	
**Age (year)**				
<12	527	409 (77.61)	1 [Ref.]	0.003
12-	439	369 (84.05)	1.10 [1.04- 1.14]	

For peer review only - http://bmjopen.bmj.com/site/about/guidelines.xhtml

≥13	450	372 (82.67)	1.07	[1.01- 1.12]	
**Governorate**					
Capital	156	127 (81.41)	1	[Ref.]	<0.001
Hawally	246	188 (76.42)	0.88	[0.73-1.01]	
Farawanya	236	183 (77.54)	0.85	[0.68-0.99]	
Jahra	239	214 (89.54)	1.10	[0.98-1.17]	
Mubarak al-Kabeer	148	124 (83.78)	1.06	[0.93-1.14]	
Ahmadi	371	296 (79.78)	1.06	[0.95-1.13]	
**Father's Education**					
Primary/Intermediate/no formal education	236	205 (86.86)	1	[Ref.]	<0.009
Secondary (high school)	344	297 (86.34)	1.04	[0.92-1.12]	
Diploma	261	220 (84.29)	1.01	[0.88-1.10]	
University & above	542	399 (73.62)	0.89	[0.76-1.01]	
**Passive smoking in household**					
No	901	704 (78.14)	1	[Ref.]	<0.006
Yes	489	426 (87.12)	1.08	[1.02-1.13]	
**Currently taking supplements**					
No	1,256	1,045 (83.20)	1	[Ref.]	<0.001
Yes	158	103 (65.19)	0.63	[0.49-0.76]	
**Consumption of sugary drinks per week**					
None	171	123 (71.93)	1	[Ref.]	0.002
Once-Three times	772	633 (81.99)	1.11	[1.05-1.16]	
Four-Six times	171	135 (78.95)	1.07	[0.96-1.14]	
Seven/more times	299	256 (85.62)	1.15	[1.08-1.19]	
**Number of times walking to school per week**					
None	1,158	982 (84.80)	1	[Ref.]	<0.001
1-8 times	155	99 (63.87)	0.81	[0.68-0.93]	
Every day	103	69 (66.99)	0.77	[0.61-0.92]	
**Body Mass Index Categories**[1]					
Normal weight	601	465 (77.37)	1	[Ref.]	0.001
Overweight	320	270 (84.38)	1.08	[1.01-1.13]	
Obese	471	400 (84.93)	1.12	[1.07-1.16]	
Underweight	24	15 (62.50)	0.93	[0.67-1.10]	

%s are row percentages. [1]according to WHO growth charts.

For peer review only - http://bmjopen.bmj.com/site/about/guidelines.xhtml

Factors associated with vitamin D status

Factors that showed significant association with vitamin D deficiency (25-OH-D < 50 nmol/L) in univariate analysis were gender, parental education, number of times per week the participants consumed breakfast or dinner prepared outside home, taking supplements, walking to school (instead of using school bus or car) in addition to BMI (Supplementary Table). Similarly, time spent outdoor per day (between 10:00 am and 4:00 pm) during weekdays and weekends in addition to wearing sunscreen and staying in shade or under an umbrella were all significant predictors in univariate analysis.

Table (2) shows the independent predictors for vitamin D deficiency in multivariate analysis. In the final model, gender, age, governorate, parental education, vitamin D supplement, and the number of times participant walks to school per week were all significantly related to vitamin D deficiency. Data from the food frequency questionnaire for calcium and vitamin D intake were available only for 200 study subjects, and of all food items, only eggs were significantly related to vitamin D deficiency in multivariate model (data not shown). We repeated the analysis above using hypovitaminosis (sufficient vs. insufficient/deficient) as a binary outcome. The same factors were found to be significantly related to hypovitaminosis, although mother's education (instead of father's education) was found to be significant in this analysis. We also used linear regression to identify the factors associated with vitamin D level as a continuous variable. Factors that showed significant association with vitamin D level in multivariate analysis were age, gender, governorate, passive smoking at household, number of times per week the participants consumed breakfast prepared outside home in the last three months, time spent outdoor during weekdays, preference to stay in shade or under the umbrella, and BMI categories. With respect to dietary factors, milk and cheese, fast food hamburger and tuna fish were significantly associated with vitamin D level in univariate linear regression. When these food items were introduced to the final model, only milk and tuna fish were significantly associated with vitamin D level. It is worth noting that the best model was able to explain only 45% of the variability in vitamin D level in this analysis.

For peer review only - http://bmjopen.bmj.com/site/about/guidelines.xhtml

Discussion

Although several reports have described vitamin D deficiency among adults in the Arab states in the Gulf region, there are limited data on the prevalence of vitamin D deficiency among healthy adolescents. We used a nationally representative sample to examine a large number of healthy adolescents, becoming to our knowledge the largest study focusing on this group in Kuwait. We used the recommended method to measure vitamin D level and demonstrated that vitamin D deficiency is too common despite the abundant sunshine. Secondary hyperparathyroidism due to vitamin D deficiency is also common and females had lower vitamin D levels compared to males.

In our setting, approximately, 81% and 15% of adolescents were vitamin D deficient or insufficient, respectively. This is higher than that reported among adolescents in European countries [25-27], Southeastern United States [28 29] or New Zealand [30]. Our finding is more akin to that reported from Saudi Arabia (95.6%) [10], India (85-98%) [8 9], or Korea where only 2.3% of adolescents were found to be vitamin D sufficient [31]. Our findings are also in accordance with the high prevalence reported consistently among adult population in Kuwait [32] or other countries in the Gulf region such as Saudi Arabia [33], Bahrain [34] and Qatar [35].

Similar to other studies among adolescents [24 28 36], our findings showed that a large number of those with low vitamin D level had evidence of secondary hyperparathyroidism. We reported a much higher prevalence of secondary hyperparathyroidism compared to other studies [24 36], which could be due to using lower PTH cut-off point to define secondary hyperthyroidism or using different laboratory method to measure PTH. Hyperparathyroidism, which potentially leads to increased bone resorption, is particularly important during adolescence because it is the critical period to achieve peak bone mass. Serum PTH decreased as 25-OH-D level increased with no clear threshold of serum 25-OH-D at which PTH level

For peer review only - http://bmjopen.bmj.com/site/about/guidelines.xhtml

plateaued although very few participants had elevated PTH when 25-OH-D level was above 75 nmol/L (Figure 1). Although we did not measure markers of bone turnover, this provides an additional evidence that 25-OH-D should be maintained above 75 nmol/L, which is the current cut-off point used to define vitamin D sufficiency in adolescents [21 22]. There were, however, adolescents with low vitamin D concentration without secondary hyperparathyroidism (Figure 1). This has been noted in other studies [28 36], the clinical significance of which is unknown in children and deserves further investigation.

Because of the abundant sunshine in the Arab states in the Gulf region, there has been a lot of skepticism of vitamin D deficiency. Informal discussions, even among scientific community, in the region show strong denial attributing such low levels of vitamin D to poorly executed or faulty laboratory measurements. Our findings should eliminate doubts on vitamin D deficiency among adolescents in Kuwait and pave the way to look for practical solutions. Factors that may hinder cutaneous synthesis of vitamin D despite abundant sunshine may include dust storms (which occur almost in one third of the year in Kuwait [37]), an avoidance of sun exposure by indoor lifestyle, head/body covering or other cultural practices and the use of sunscreen. Self-reported exposure to sunlight was a significant predictor for vitamin D deficiency in univariate analysis but lost statistical significance in multivariate analysis. Most of adolescents in our setting avoid sunlight as only around 6% of the participants spent more than two hours outdoor per day between 10:00 am and 4:00 pm during weekdays (data not shown). Avoidance of sunlight has been suggested to be the underlying reason for the counterintuitive seasonal variation that has been reported in the region, where the prevalence of vitamin D deficiency is higher in the summer compared to the winter season [36]. Owing to the desert climate, avoiding sun exposure would be stronger during summer due to extremely high temperature. We collected blood samples in February-April and thus we were unable to describe seasonal variation in vitamin D status. Exposure to sunlight contributes up to 90–95% of the vitamin D supply, while the number of foods naturally containing a significant quantity of vitamin D is very limited except for some

For peer review only - http://bmjopen.bmj.com/site/about/guidelines.xhtml

oily fish that is rarely consumed by adolescents worldwide and in our setting [38] (~5% of adolescents in our setting consumed salmon once per week or more). As such, education of parents and adolescents on the safe amount of sun exposure in addition to changes in the school environment to facilitate exposure to sunlight may produce the highest impact on vitamin D status among adolescents. There is no consensus on the duration of time, which adolescents can safely be exposed to direct sunlight. However, exposure of legs and arms for at least 15 minutes twice per week has been reported to be sufficient for adequate sun-induced cutaneous vitamin D synthesis in adolescents [38].

Similar to several studies that investigated vitamin D deficiency among adolescents, females had a significantly higher prevalence of vitamin D deficiency compared to males even after adjusting for all potential confounders. Also, vitamin D level was significantly lower among females compared to males. Similar findings have been reported among adolescents from Saudi Arabia [39], India [9], Korea [31] and Taiwan [40]. This pattern is not common in other settings as reported from a clinic-based cross-sectional study in the US [28] or Italy [26]. In the Arab states in the Gulf region and India, cultural practices, such as type of clothing that covers the whole body and indoor lifestyle, have been proposed to exacerbate vitamin D deficiency among females. Indeed, we found males consistently reporting higher amount of outdoor exposure to sunlight compared to females. In addition, females reported consistently higher sun avoidance behavior such as using sunscreen and staying in shade or under an umbrella. Adjusting for the exposure to sunlight did not explain the whole association between vitamin D status and gender which could be due to residual confounding. We also collected data on the type of clothing among females using photo cards (no head covering, head covering, head and face covering), but vitamin D deficiency was too common among females, which did not allow for us to investigate the impact of type of dress on vitamin D status among females. In our setting, efforts to improve vitamin D level among adolescents should focus on females through encouragement of safe amount of exposure to sunlight and intake of vitamin D rich foods. Also the

For peer review only - http://bmjopen.bmj.com/site/about/guidelines.xhtml

current recommended doses for vitamin D supplement should take the difference between females and males into account and recommend higher doses for females compared to males.

Most of the students in Kuwait do not walk to schools and are mainly transported by cars or school buses. The number of times student walk to/from school as well as the time spent on walking to/from school was significantly associated with vitamin D deficiency. Those who walk to/from schools were less likely to have vitamin D deficiency compared to those who used school bus or car. Walking to schools can be a good opportunity for sunlight exposure as well as increasing physical activity, which itself may improve vitamin D level. In a study in the US, physical activity was inversely associated with hypovitaminosis D but not related to vitamin D level [28] while in another study, vigorous physical activity was significantly associated with vitamin D level [29]. It has been proposed that physical activity may increase the level of vitamin D through increasing the time spent outdoor in sunlight or through reducing the risk of obesity [13]. However, in our study the total time spent on other physical/sport activities was significantly associated with vitamin D deficiency in univariate but not in multivariate analysis. Unlike walking to schools, most sport activities in our setting are practiced indoor or during night time due to severe hot weather which may explain the lack of association between total time spent on physical activities and vitamin D deficiency in multivariate analysis. Like many other studies [25 28 31], BMI was inversely associated with vitamin D level, which has been attributed to the sequestration of vitamin D within the plentiful adipose tissue [41 42]. It has also been suggested that leptin, an adipocyte-derived hormone, might activate a pathway that inhibits renal synthesis of the active form of vitamin D [43].

In our study, sugary drinks were positively associated with the prevalence of vitamin D deficiency. This is similar to that reported from Saudi Arabia [36] and US [28]. Consumption of sugary drinks among adolescents usually occurs at the expense of milk consumption which contains vitamin D [44]. As a result, it has been proposed that the availability of vitamin D-fortified juices may help reduce vitamin D deficiency in

For peer review only - http://bmjopen.bmj.com/site/about/guidelines.xhtml

adolescents [28]. In our data those who consumed sugary drinks were less likely to consume milk (data not shown). Although milk consumption was not related to vitamin D deficiency it was significantly related to the vitamin D level in linear regression analysis.

We found an association between passive smoking at household (reported by parents) and vitamin D deficiency among adolescents, which is interesting and has been reported before [40]. Only less than 1% of the adolescents in our setting reported active smoking. Smoking has also been reported to be a significant determinant for low vitamin D levels among adults in some studies [27] but not in others [45] and this issue remains controversial [27]. Furthermore, a recent study found no significant association between cotinine level and vitamin D deficiency among Korean adolescents [31]. One of the hypothesized mechanism is that smoking may reflect an overall less healthy life style including less physical activity and poor dietary habits [27]. A causal link has also been proposed as metabolites in cigarette's smoke can inhibit CYP27A1 activity [46], which is involved in vitamin D metabolism.

Strengths and limitation

This is the first study that measured vitamin D in a large nationally representative sample of adolescents in Kuwait. We used a measurement method that is recommended in epidemiological studies and gathered data from both parents and adolescents. However, we did not measure the skin color, which can be an important determinant for vitamin D synthesis in the skin during exposure to sunlight. We evaluated the dietary intake of vitamin D in only a subgroup of study participants. However, dietary intake of vitamin D is known to have a smaller contribution to vitamin D status compared to cutaneous synthesis in response to sun exposure [38] and data on vitamin D supplement were obtained from all participants.

Conclusion

In conclusion, a high prevalence of vitamin D deficiency was noted among adolescents in Kuwait despite the abundant sunshine. This may reflect strong sun avoidance behavior as only less than 4% of adolescents

For peer review only - http://bmjopen.bmj.com/site/about/guidelines.xhtml

were vitamin D sufficient and most of them were on vitamin D supplements. An optimal vitamin D level is essential during adolescence; and vitamin D deficiency at this age represents a public health problem that should be addressed. First, our findings should help clear doubts on vitamin D deficiency among adolescents in Kuwait, which is a prerequisite for prevention initiatives. Second, sun exposure is the main source for vitamin D in this age group; therefore adequate outdoor daytime activities should be encouraged especially for females. Slight modifications in school environment to facilitate exposure to sunlight during school breaks between classes can be fruitful. With respect to vitamin D supplementation, we call for locally tailored guidelines in which females should have a higher amount of vitamin D supplement compared to males. Vitamin D fortification particularly for food products that are popular for adolescents in our setting should also be considered. Increasing exposure to sunlight and food fortification with vitamin D should not be considered mutually exclusive and both strategies can be adopted.

Acknowledgements

The authors acknowledge the assistance of Nadien Sameeh Rushdi and the team of data collectors. We also acknowledge the cooperation of all participating schools and the facilitation of the project by the Ministry of Education. We also appreciate the support and cooperation of the staff and management of The United Genetics Laboratories (Kuwait) in vitamin D analysis.

Authors' contributions

AT contributed to the study design and data collection, analyzed the data and drafted the paper. He had full access to all the data and took responsibility for the integrity of the data. AR contributed to the design of the study and data collection in addition to the writing and revising the manuscript. RS and LS contributed to the design of the study and data collection in addition to revising the manuscript with significant intellectual input. AH contributed to the data collection and revised the manuscript.

For peer review only - http://bmjopen.bmj.com/site/about/guidelines.xhtml

Funding: The work was supported and funded by Kuwait University- Research Project No. WF 02/13.

Competing interests: The authors declare that they have no competing interests

Data sharing statement: No additional data are available.

For peer review only

For peer review only - http://bmjopen.bmj.com/site/about/guidelines.xhtml

References

1. Mouratidou T, Vicente-Rodriguez G, Gracia-Marco L, et al. Associations of dietary calcium, vitamin D, milk intakes, and 25-hydroxyvitamin D with bone mass in Spanish adolescents: the HELENA study. Journal of clinical densitometry : the official journal of the International Society for Clinical Densitometry 2013;**16**(1):110-7 doi: 10.1016/j.jocd.2012.07.008[published Online First: Epub Date]|.

2. Sharief S, Jariwala S, Kumar J, Muntner P, Melamed ML. Vitamin D levels and food and environmental allergies in the United States: results from the National Health and Nutrition Examination Survey 2005-2006. The Journal of allergy and clinical immunology 2011;**127**(5):1195-202 doi: 10.1016/j.jaci.2011.01.017[published Online First: Epub Date]|.

3. Lucato P, Solmi M, Maggi S, et al. Low vitamin D levels increase the risk of type 2 diabetes in older adults: A systematic review and meta-analysis. Maturitas 2017;**100**:8-15 doi: 10.1016/j.maturitas.2017.02.016[published Online First: Epub Date]|.

4. Bahrami A, Mazloum SR, Maghsoudi S, et al. High Dose Vitamin D Supplementation Is Associated With a Reduction in Depression Score Among Adolescent Girls: A Nine-Week Follow-Up Study. Journal of dietary supplements 2017:1-10 doi: 10.1080/19390211.2017.1334736[published Online First: Epub Date]|.

5. Chowdhury R, Kunutsor S, Vitezova A, et al. Vitamin D and risk of cause specific death: systematic review and meta-analysis of observational cohort and randomised intervention studies. BMJ (Clinical research ed.) 2014;**348**:g1903 doi: 10.1136/bmj.g1903[published Online First: Epub Date]|.

6. Hilger J, Friedel A, Herr R, et al. A systematic review of vitamin D status in populations worldwide. The British journal of nutrition 2014;**111**(1):23-45 doi: 10.1017/s0007114513001840[published Online First: Epub Date]|.

7. Basatemur E, Horsfall L, Marston L, Rait G, Sutcliffe A. Trends in the Diagnosis of Vitamin D Deficiency. Pediatrics 2017;**139**(3) doi: 10.1542/peds.2016-2748[published Online First: Epub Date]|.

8. Garg MK, Marwaha RK, Khadgawat R, et al. Efficacy of vitamin D loading doses on serum 25-hydroxy vitamin D levels in school going adolescents: an open label non-randomized prospective trial. Journal of pediatric endocrinology & metabolism : JPEM 2013;**26**(5-6):515-23 doi: 10.1515/jpem-2012-0390[published Online First: Epub Date]|.

9. Kapil U, Pandey RM, Goswami R, et al. Prevalence of Vitamin D deficiency and associated risk factors among children residing at high altitude in Shimla district, Himachal Pradesh, India. Indian journal of endocrinology and metabolism 2017;**21**(1):178-83 doi: 10.4103/2230-8210.196031[published Online First: Epub Date]|.

10. AlBuhairan FS, Tamim H, Al Dubayee M, et al. Time for an Adolescent Health Surveillance System in Saudi Arabia: Findings From "Jeeluna". The Journal of adolescent health : official publication of the Society for Adolescent Medicine 2015;**57**(3):263-9 doi: 10.1016/j.jadohealth.2015.06.009[published Online First: Epub Date]|.

11. Kaddam IM, Al-Shaikh AM, Abaalkhail BA, et al. Prevalence of vitamin D deficiency and its associated factors in three regions of Saudi Arabia. Saudi medical journal 2017;**38**(4):381-90 doi: 10.15537/smj.2017.4.18753[published Online First: Epub Date]|.

12. Mansour MM, Alhadidi KM. Vitamin D deficiency in children living in Jeddah, Saudi Arabia. Indian journal of endocrinology and metabolism 2012;**16**(2):263-9 doi: 10.4103/2230-8210.93746[published Online First: Epub Date]|.

13. Valtuena J, Gonzalez-Gross M, Huybrechts I, et al. Factors associated with vitamin D deficiency in European adolescents: the HELENA study. Journal of nutritional science and vitaminology 2013;**59**(3):161-71

For peer review only - http://bmjopen.bmj.com/site/about/guidelines.xhtml

14. Glanz K, Yaroch AL, Dancel M, et al. Measures of sun exposure and sun protection practices for behavioral and epidemiologic research. Archives of dermatology 2008;**144**(2):217-22 doi: 10.1001/archdermatol.2007.46[published Online First: Epub Date]|.

15. UEA. Youth Physical Activity Questionnaire. Secondary Youth Physical Activity Questionnaire 2011. http://epi-meta.medschl.cam.ac.uk/includes/speedy/pdf/PhysicalActivity%20V4%20SPEEDY-3.pdf.

16. Al-Hazzaa HM, Al-Sobayel HI, Musaiger AO. Convergent validity of the Arab Teens Lifestyle Study (ATLS) physical activity questionnaire. International journal of environmental research and public health 2011;**8**(9):3810-20 doi: 10.3390/ijerph8093810[published Online First: Epub Date]|.

17. Taylor C, Lamparello B, Kruczek K, Anderson EJ, Hubbard J, Misra M. Validation of a food frequency questionnaire for determining calcium and vitamin D intake by adolescent girls with anorexia nervosa. Journal of the American Dietetic Association 2009;**109**(3):479-85, 85 e1-3 doi: 10.1016/j.jada.2008.11.025[published Online First: Epub Date]|.

18. Papandreou D, achaniotis N, Lari M, Al Mussabi W. Validation of a Food Frequency Questionnaire for Vitamin D and Calcium Intake in Healthy Female College Students. Food and Nutrition Sciences 2014;**5**:2048-52

19. Holick MF. Vitamin D deficiency. The New England journal of medicine 2007;**357**(3):266-81 doi: 10.1056/NEJMra070553[published Online First: Epub Date]|.

20. Vogeser M. Quantification of circulating 25-hydroxyvitamin D by liquid chromatography-tandem mass spectrometry. The Journal of steroid biochemistry and molecular biology 2010;**121**(3-5):565-73 doi: 10.1016/j.jsbmb.2010.02.025[published Online First: Epub Date]|.

21. Holick MF, Binkley NC, Bischoff-Ferrari HA, et al. Evaluation, treatment, and prevention of vitamin D deficiency: an Endocrine Society clinical practice guideline. The Journal of clinical endocrinology and metabolism 2011;**96**(7):1911-30 doi: 10.1210/jc.2011-0385[published Online First: Epub Date]|.

22. Medicine SfAHa. Recommended vitamin D intake and management of low vitamin D status in adolescents: a position statement of the society for adolescent health and medicine. The Journal of adolescent health : official publication of the Society for Adolescent Medicine 2013;**52**(6):801-3 doi: 10.1016/j.jadohealth.2013.03.022[published Online First: Epub Date]|.

23. Braegger C, Campoy C, Colomb V, et al. Vitamin D in the healthy European paediatric population. Journal of pediatric gastroenterology and nutrition 2013;**56**(6):692-701 doi: 10.1097/MPG.0b013e31828f3c05[published Online First: Epub Date]|.

24. Vierucci F, Del Pistoia M, Fanos M, Erba P, Saggese G. Prevalence of hypovitaminosis D and predictors of vitamin D status in Italian healthy adolescents. Italian journal of pediatrics 2014;**40**:54 doi: 10.1186/1824-7288-40-54[published Online First: Epub Date]|.

25. Gonzalez-Gross M, Valtuena J, Breidenassel C, et al. Vitamin D status among adolescents in Europe: the Healthy Lifestyle in Europe by Nutrition in Adolescence study. The British journal of nutrition 2012;**107**(5):755-64 doi: 10.1017/s0007114511003527[published Online First: Epub Date]|.

26. Vierucci F, Del Pistoia M, Fanos M, et al. Vitamin D status and predictors of hypovitaminosis D in Italian children and adolescents: a cross-sectional study. European journal of pediatrics 2013;**172**(12):1607-17 doi: 10.1007/s00431-013-2119-z[published Online First: Epub Date]|.

27. Kassi EN, Stavropoulos S, Kokkoris P, et al. Smoking is a significant determinant of low serum vitamin D in young and middle-aged healthy males. Hormones (Athens, Greece) 2015;**14**(2):245-50 doi: 10.14310/horm.2002.1521[published Online First: Epub Date]|.

For peer review only - http://bmjopen.bmj.com/site/about/guidelines.xhtml

28. Gordon CM, DePeter KC, Feldman HA, Grace E, Emans SJ. Prevalence of vitamin D deficiency among healthy adolescents. Archives of pediatrics & adolescent medicine 2004;**158**(6):531-7 doi: 10.1001/archpedi.158.6.531[published Online First: Epub Date]|.

29. Dong Y, Pollock N, Stallmann-Jorgensen IS, et al. Low 25-hydroxyvitamin D levels in adolescents: race, season, adiposity, physical activity, and fitness. Pediatrics 2010;**125**(6):1104-11 doi: 10.1542/peds.2009-2055[published Online First: Epub Date]|.

30. Rockell JE, Green TJ, Skeaff CM, et al. Season and ethnicity are determinants of serum 25-hydroxyvitamin D concentrations in New Zealand children aged 5-14 y. The Journal of nutrition 2005;**135**(11):2602-8

31. Byun EJ, Heo J, Cho SH, Lee JD, Kim HS. Suboptimal vitamin D status in Korean adolescents: a nationwide study on its prevalence, risk factors including cotinine-verified smoking status and association with atopic dermatitis and asthma. BMJ open 2017;**7**(7):e016409 doi: 10.1136/bmjopen-2017-016409[published Online First: Epub Date]|.

32. Zhang FF, Al Hooti S, Al Zenki S, et al. Vitamin D deficiency is associated with high prevalence of diabetes in Kuwaiti adults: results from a national survey. BMC public health 2016;**16**:100 doi: 10.1186/s12889-016-2758-x[published Online First: Epub Date]|.

33. Al-Daghri NM. Vitamin D in Saudi Arabia: Prevalence,distribution and disease associations. The Journal of steroid biochemistry and molecular biology 2016 doi: 10.1016/j.jsbmb.2016.12.017[published Online First: Epub Date]|.

34. Golbahar J, Al-Saffar N, Altayab Diab D, Al-Othman S, Darwish A, Al-Kafaji G. Predictors of vitamin D deficiency and insufficiency in adult Bahrainis: a cross-sectional study. Public health nutrition 2014;**17**(4):732-8 doi: 10.1017/s136898001300030x[published Online First: Epub Date]|.

35. Mahdy S, Al-Emadi SA, Khanjar IA, et al. Vitamin D status in health care professionals in Qatar. Saudi medical journal 2010;**31**(1):74-7

36. Sulimani RA, Mohammed AG, Alfadda AA, et al. Vitamin D deficiency and biochemical variations among urban Saudi adolescent girls according to season. Saudi medical journal 2016;**37**(9):1002-8 doi: 10.15537/smj.2016.9.15248[published Online First: Epub Date]|.

37. Thalib L, Al-Taiar A. Dust storms and the risk of asthma admissions to hospitals in Kuwait. The Science of the total environment 2012;**433**:347-51 doi: 10.1016/j.scitotenv.2012.06.082[published Online First: Epub Date]|.

38. Holick MF. The D-lightful vitamin D for child health. JPEN. Journal of parenteral and enteral nutrition 2012;**36**(1 Suppl):9S-19S doi: 10.1177/0148607111430189[published Online First: Epub Date]|.

39. Al-Daghri NM, Al-Saleh Y, Aljohani N, et al. Vitamin D Deficiency and Cardiometabolic Risks: A Juxtaposition of Arab Adolescents and Adults. PloS one 2015;**10**(7):e0131315 doi: 10.1371/journal.pone.0131315[published Online First: Epub Date]|.

40. Yao TC, Tu YL, Chang SW, et al. Suboptimal vitamin D status in a population-based study of Asian children: prevalence and relation to allergic diseases and atopy. PloS one 2014;**9**(6):e99105 doi: 10.1371/journal.pone.0099105[published Online First: Epub Date]|.

41. Wortsman J, Matsuoka LY, Chen TC, Lu Z, Holick MF. Decreased bioavailability of vitamin D in obesity. The American journal of clinical nutrition 2000;**72**(3):690-3

42. Walker GE, Ricotti R, Roccio M, et al. Pediatric obesity and vitamin D deficiency: a proteomic approach identifies multimeric adiponectin as a key link between these conditions. PloS one 2014;**9**(1):e83685 doi: 10.1371/journal.pone.0083685[published Online First: Epub Date]|.

For peer review only - http://bmjopen.bmj.com/site/about/guidelines.xhtml

43. Tsuji K, Maeda T, Kawane T, Matsunuma A, Horiuchi N. Leptin stimulates fibroblast growth factor 23 expression in bone and suppresses renal 1alpha,25-dihydroxyvitamin D3 synthesis in leptin-deficient mice. J Bone Miner Res 2010;**25**(8):1711-23 doi: 10.1002/jbmr.65[published Online First: Epub Date]|.

44. O'Connor TM, Yang SJ, Nicklas TA. Beverage intake among preschool children and its effect on weight status. Pediatrics 2006;**118**(4):e1010-8 doi: 10.1542/peds.2005-2348[published Online First: Epub Date]|.

45. Scragg R, Holdaway I, Jackson R, Lim T. Plasma 25-hydroxyvitamin D3 and its relation to physical activity and other heart disease risk factors in the general population. Annals of epidemiology 1992;**2**(5):697-703

46. Aboraia AS, Makowski B, Bahja A, et al. Synthesis and CYP24A1 inhibitory activity of (E)-2-(2-substituted benzylidene)- and 2-(2-substituted benzyl)-6-methoxy-tetralones. European journal of medicinal chemistry 2010;**45**(10):4427-34 doi: 10.1016/j.ejmech.2010.07.001[published Online First: Epub Date]|.

For peer review only

Figure legends

Figure (1): Parathyroid hormone (PTH) with Serum 25-hydroxyvitamin D (25-OH-D) concentration. Individuals above the horizontal line (PTH ≥ 65.0 ng/L) are adolescents with secondary hyperthyroidism. Vertical lines represent the 25-OH-D cut-off used to define vitamin D deficiency, insufficiency and sufficiency (seven participants with 25-OH-D>150 nmol/L were omitted).

For peer review only - http://bmjopen.bmj.com/site/about/guidelines.xhtml

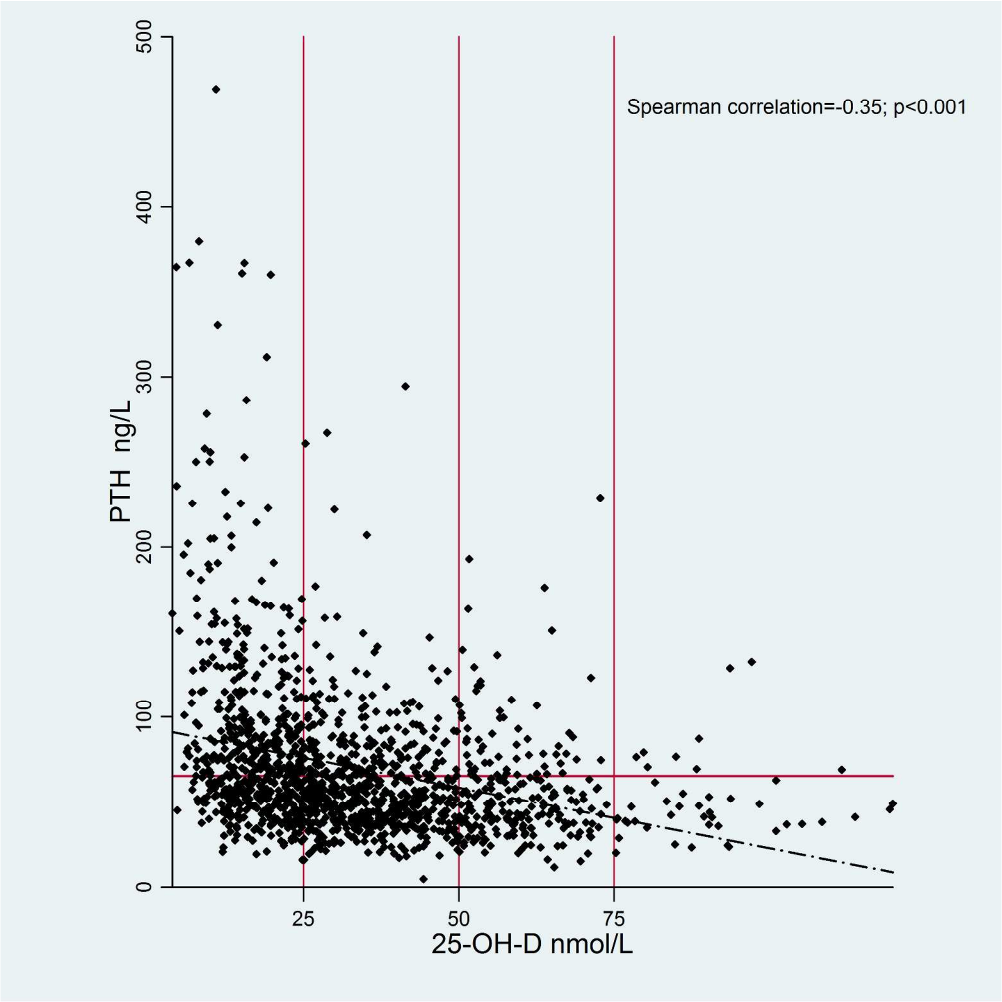

Figure (1): Parathyroid hormone (PTH) with Serum 25-hydroxyvitamin D (25-OH-D) concentration. Individuals above the horizontal line (PTH ≥ 65.0 ng/L) are adolescents with secondary hyperthyroidism. Vertical lines represent the 25-OH-D cut-off used to define vitamin D deficiency, insufficiency and sufficiency (seven participants with 25-OH-D>150 nmol/L were omitted).

1058x1058mm (72 x 72 DPI)

Supplementary Table (1). Factors associated with vitamin D deficiency among adolescents in univariate analysis.

Characteristics	Total	Prevalence of vitamin D deficiency n=1150	(%)	Prevalence Ratio [95%CI]		p
**Gender**						
Male	694	488	(70.32)	1	[Ref.]	<0.001
Female	722	662	(91.69)	1.30	[1.26- 1.33]	
**Age (year)**						
<12	527	409	(77.61)	1	[Ref.]	0.024
12-	439	369	(84.05)	1.07	[1.01- 1.12]	
≥13	450	372	(82.67)	1.06	[1.00- 1.10]	
**Nationality**						
Kuwaiti	1081	900	(83.26)	1	[Ref.]	<0.001
Non-Kuwait	335	250	(74.63)	0.90	[0.82-0.96]	
**Governorate**						
Capital	156	127	(81.41)	1	[Ref.]	0.003
Hawally	246	188	(76.42)	0.94	[0.82-1.03]	
Farawanya	236	183	(77.54)	0.95	[0.83-1.04]	
Jahra	239	214	(89.54)	1.10	[1.02-1.15]	
Mubarak al-Kabeer	148	124	(83.78)	1.03	[0.91-1.10]	
Ahmadi	371	296	(79.78)	0.98	[0.88-1.06]	
**Father's Education**						
Primary/Intermediate/no formal education	236	205	(86.86)	1	[Ref.]	<0.001
Secondary (high school)	344	297	(86.34)	0.99	[0.87-1.08]	
Diploma	261	220	(84.29)	0.95	[0.82-1.06]	
University & above	542	399	(73.62)	0.78	[0.65-0.90]	
**Mother's Education**						
Primary/Intermediate/no formal education	183	153	(83.61)	1	[Ref.]	<0.001
Secondary (high school)	304	263	(86.51)	1.04	[0.94-1.12]	
Diploma	304	259	(85.20)	1.02	[0.91-1.10]	
University & above	605	457	(75.54)	0.88	[0.76-0.99]	
**Total number of brother/sisters**						
Zero-two	298	223	(74.83)	1	[Ref.]	0.002
Three-four	538	434	(80.67)	1.05	[1.00-1.10]	
Five or more	553	469	(84.81)	1.10	[1.04-1.13]	
**Passive smoking at household**						
No	901	704	(78.14)	1	[Ref.]	<0.001
Yes	489	426	(87.12)	1.11	[1.06-1.15]	

**Times per week consumed breakfast not prepared at home**							
Zero	624	484	(77.56)	1	[Ref.]	0.019
One-two times	569	478	(84.01)	1.08	[1.02-1.12]	
Three-four	117	98	(83.76)	1.07	[0.97-1.14]	
Five or more	78	67	(85.90)	1.10	[1.00-1.17]	
**Times per week consumed dinner not prepared at home**						
Zero	157	114	(72.61)	1	[Ref.]	0.018
One-two times	878	719	(81.89)	1.09	[1.03-1.14]	
Three-four	257	211	(82.10)	1.09	[1.01-1.15]	
Five or more	86	75	(87.21)	1.14	[1.04-1.19]	
**Consumption of sugary drinks per week**						
None	171	123	(71.93)	1	[Ref.]	0.002
Once-Three times	772	633	(81.99)	1.09	[1.04-1.14]	
Four-Six	171	135	(78.95)	1.07	[0.98-1.13]	
Seven/more	299	256	(85.62)	1.12	[1.07-1.17]	
**Currently taking supplements**						
No	1,256	1,045	(83.20)	1	[Ref.]	<0.001
Yes	158	103	(65.19)	0.78	[0.68-0.88]	
**Number of times walking to school per week**						
None	1,158	982	(84.80)	1	[Ref.]	<0.001
1-8 times	155	99	(63.87)	0.74	[0.63-0.83]	
Every day	103	69	(66.99)	0.77	[0.65-0.89]	
**Time walking to school**						
None	1,158	982	(84.80)	1	[Ref.]	<0.001
≤5 minutes	80	50	(62.50)	0.71	[0.56-0.84]	
6- 10 minutes	76	53	(69.74)	0.80	[0.65-0.93]	
11-15	56	38	(67.86)	0.77	[0.60-0.92]	
16 minutes or more	45	26	(57.78)	0.65	[0.46-0.82]	
**Time spent on physical activity per week**						
Low (lower tertile)	469	396	(84.43)	1	[Ref.]	0.005
Medium (middle tertile)	473	391	(82.66)	0.97	[0.89-1.04]	
High (higher tertile)	474	363	(76.58)	0.89	[0.80-0.96]	
**Body Mass Index Categories[1]**						
Normal weight	601	465	(77.37)	1	[Ref.]	0.001
Overweight	320	270	(84.38)	1.07	[1.02-1.11]	

Obese	471	400 (84.93)	1.08	[1.03-1.11]	
Under weight	24	15 (62.50)	0.84	[0.59-1.02]	
**Time outside per day 10:00 am- 4:00 pm week days**					
Less than 30 minutes	657	563 (85.69)	1	[Ref.]	<0.001
31 minutes to 1 hour	450	342 (76.00)	0.87	[0.80-0.94]	
More than 1 hour to 2 hours	222	178 (80.18)	0.93	[0.84-1.00]	
More than 2 hours	87	67 (77.01)	0.88	[0.74-0.99]	
**Time outside per day 10:00 am- 4:00 pm week ends**					
Less than 30 minutes	494	425 (86.03)	1	[Ref.]	0.003
31 minutes to 1 hour	312	257 (82.37)	0.94	[0.85-1.02]	
More than 1 hour to 2 hours	255	198 (77.65)	0.87	[0.76-0.96]	
More than 2 hours to 3 hours	185	142 (76.76)	0.85	[0.74-0.96]	
More than 3 hours	169	127 (75.15)	0.83	[0.71-0.94]	
**Wearing sunscreen**					
Never	1,100	874 (79.45)	1	[Ref.]	0.013
Rarely	77	65 (84.42)	1.06	[0.94-1.13]	
Sometimes	160	140 (87.50)	1.09	[1.02-1.14]	
Often/always	79	71 (89.87)	1.12	[1.02-1.18]	
**Staying in shade or under an umbrella**					
Never	493	396 (80.32)	1	[Ref.]	0.04
Rarely	97	70 (72.16)	0.90	[0.78-1.01]	
Sometimes	363	301 (82.92)	1.03	[0.96-1.08]	
Often	205	162 (79.02)	0.98	[0.90-1.05]	
Always	257	220 (85.60)	1.06	[0.99-1.11]	

%s are row percentages. [1]according to WHO growth charts.

For peer review only - http://bmjopen.bmj.com/site/about/guidelines.xhtml

STROBE 2007 (v4) checklist of items to be included in reports of observational studies in epidemiology*
Checklist for cohort, case-control, and cross-sectional studies (combined)

Section/Topic	Item #	Recommendation	Reported on page #
**Title and abstract**	1	(*a*) Indicate the study's design with a commonly used term in the title or the abstract	Page 1
		(*b*) Provide in the abstract an informative and balanced summary of what was done and what was found	Page 3
**Introduction**			
Background/rationale	2	Explain the scientific background and rationale for the investigation being reported	Page 5
Objectives	3	State specific objectives, including any pre-specified hypotheses	Page 5
**Methods**			
Study design	4	Present key elements of study design early in the paper	Page 6
Setting	5	Describe the setting, locations, and relevant dates, including periods of recruitment, exposure, follow-up, and data collection	Page 6- Page 8 and Page 14
Participants	6	(*a*) *Cohort study*—Give the eligibility criteria, and the sources and methods of selection of participants. Describe methods of follow-up<br>*Case-control study*—Give the eligibility criteria, and the sources and methods of case ascertainment and control selection. Give the rationale for the choice of cases and controls<br>*Cross-sectional study*—Give the eligibility criteria, and the sources and methods of selection of participants	Page 6
		(*b*) *Cohort study*—For matched studies, give matching criteria and number of exposed and unexposed<br>*Case-control study*—For matched studies, give matching criteria and the number of controls per case	
Variables	7	Clearly define all outcomes, exposures, predictors, potential confounders, and effect modifiers. Give diagnostic criteria, if applicable	Page 6 and Page 7
Data sources/ measurement	8*	For each variable of interest, give sources of data and details of methods of assessment (measurement). Describe comparability of assessment methods if there is more than one group	*Page 6 and Page 7*
Bias	9	Describe any efforts to address potential sources of bias	Page 6, Page 12 and Page 17
Study size	10	Explain how the study size was arrived at	Page 13
Quantitative variables	11	Explain how quantitative variables were handled in the analyses. If applicable, describe which groupings were chosen and why	Page 7 and Page 8
Statistical methods	12	(*a*) Describe all statistical methods, including those used to control for confounding	Page 7 and Page 8
		(*b*) Describe any methods used to examine subgroups and interactions	Page 7 and Page 8
		(*c*) Explain how missing data were addressed	These were minimum.

For peer review only

		(*d*) *Cohort study*—If applicable, explain how loss to follow-up was addressed<br>*Case-control study*—If applicable, explain how matching of cases and controls was addressed<br>*Cross-sectional study*—If applicable, describe analytical methods taking account of sampling strategy	Page 8
		(*e*) Describe any sensitivity analyses	
**Results**			
Participants	13*	(a) Report numbers of individuals at each stage of study—eg numbers potentially eligible, examined for eligibility, confirmed eligible, included in the study, completing follow-up, and analysed	Page 8
		(b) Give reasons for non-participation at each stage	Page 8
		(c) Consider use of a flow diagram	
Descriptive data	14*	(a) Give characteristics of study participants (eg demographic, clinical, social) and information on exposures and potential confounders	Table 1 page 9
		(b) Indicate number of participants with missing data for each variable of interest	Table 1 , Table 2 and also supplementary table
		(c) *Cohort study*—Summarise follow-up time (eg, average and total amount)	
Outcome data	15*	*Cohort study*—Report numbers of outcome events or summary measures over time	
		Case-control study—Report numbers in each exposure category, or summary measures of exposure	
		Cross-sectional study—Report numbers of outcome events or summary measures	Page 10
Main results	16	(*a*) Give unadjusted estimates and, if applicable, confounder-adjusted estimates and their precision (eg, 95% confidence interval). Make clear which confounders were adjusted for and why they were included	
		(*b*) Report category boundaries when continuous variables were categorized	All tables
		(*c*) If relevant, consider translating estimates of relative risk into absolute risk for a meaningful time period	
Other analyses	17	Report other analyses done—eg analyses of subgroups and interactions, and sensitivity analyses	Page 12
**Discussion**			
Key results	18	Summarise key results with reference to study objectives	Page 13
Limitations	19	Discuss limitations of the study, taking into account sources of potential bias or imprecision. Discuss both direction and magnitude of any potential bias	Page 16
Interpretation	20	Give a cautious overall interpretation of results considering objectives, limitations, multiplicity of analyses, results from similar studies, and other relevant evidence	Page 17
Generalisability	21	Discuss the generalisability (external validity) of the study results	Page 17
**Other information**			
Funding	22	Give the source of funding and the role of the funders for the present study and, if applicable, for the original study on which the present article is based	Page 18

For peer review only

*Give information separately for cases and controls in case-control studies and, if applicable, for exposed and unexposed groups in cohort and cross-sectional studies.

Note: An Explanation and Elaboration article discusses each checklist item and gives methodological background and published examples of transparent reporting. The STROBE checklist is best used in conjunction with this article (freely available on the Web sites of PLoS Medicine at http://www.plosmedicine.org/, Annals of Internal Medicine at http://www.annals.org/, and Epidemiology at http://www.epidem.com/). Information on the STROBE Initiative is available at www.strobe-statement.org.

For peer review only

BMJ Open

Vitamin D Status among Adolescents in Kuwait: A Cross-sectional Study.

Journal:	*BMJ Open*
Manuscript ID	bmjopen-2017-021401.R3
Article Type:	Research
Date Submitted by the Author:	29-Jun-2018
Complete List of Authors:	Al-Taiar, Abdullah; Faculty of Medicine, Kuwait University, Department of Community Medicine and Behavioural Sciences<br>Rahman , Abdur ; College of Life Sciences, Kuwait University, Department of Food Science and Nutrition<br>Al-Sabah, Reem; Faculty of Medicine, Kuwait University, Department of Community Medicine and Behavioural Sciences<br>Shaban, Lemia ; College of Life Sciences, Kuwait University, Department of Food Science and Nutrition<br>Al-Harbi, Anwar ; Kuwait Institute for Scientific Research , Department of Science and Nutrition
**Primary Subject Heading**:	Nutrition and metabolism
Secondary Subject Heading:	Epidemiology, Global health, Paediatrics, Public health
Keywords:	PUBLIC HEALTH, Vitamin D, Adolescents, school children, Kuwait

SCHOLARONE™
Manuscripts

Vitamin D Status among Adolescents in Kuwait: A Cross-sectional Study.

Abdullah Al-Taiar[1], Abdur Rahman[2], Reem Al-Sabah[1], Lemia Shaban[2], Anwar Al-Harbi[3]

[1] Department of Community Medicine and Behavioural Sciences

Faculty of Medicine, Kuwait University,

Box: 24923 Safat-13110 Kuwait.

[2] Department of Food Science and Nutrition

College of Life Sciences, Kuwait University,

Box 5969, Safat-13060, Kuwait.

[3] Kuwait Institute for Scientific Research,

Department of Science and Nutrition,

Box: 24885 Safat-13109, Kuwait.

Before publication corresponding author:

Dr Abdullah Al-Taiar

Associate Professor of Epidemiology

Department Community Medicine and Behavioural Sciences,

Faculty of Medicine, Kuwait University,

Box: 24923 Safat-13110 Kuwait.

Tel: +965-24986553

Fax: +965-5338948

E-mail: altaiar@hsc.edu.kw

For peer review only

For peer review only - http://bmjopen.bmj.com/site/about/guidelines.xhtml

After publication corresponding author:

Abdur Rahman

Associate Professor

Department of Food Science and Nutrition,

College of Life Sciences, Kuwait University,

Box: 5969 Safat-13060 Kuwait.

Tel: +965-249633055

Fax: +965-22513929

E-mail: abdurrahman.ahmad@ku.edu.kw

For peer review only

Abstract

Objectives

In Kuwait, as in many Arab states in the gulf region, there are limited data on the prevalence of vitamin D deficiency among healthy adolescents. This study aimed to estimate the prevalence of vitamin D deficiency in a nationally representative sample of adolescents and investigate factors associated with vitamin D status.

Methods

A cross-sectional study was conducted on 1416 adolescents aged 11-16 years, who were randomly selected from middle schools in all governorates of Kuwait. Data were collected from parents through self-administered questionnaire and from adolescents through face-to-face interview. Vitamin D was measured using liquid chromatography-tandem mass spectrometry (LC-MS/MS). Logistic regression was used to investigate the independent factors associated with vitamin D status.

Results

The Prevalence of vitamin D deficiency was 81.21% (95%CI: 71.61-90.81%) while severe deficiency was 39.48%. Only 3.60% of adolescents were vitamin D sufficient. Prevalence of vitamin D deficiency was significantly higher among females compared to males (91.69% vs. 70.32%; p<0.001). There was a significant inverse correlation between vitamin D and Parathyroid hormone (Spearman correlation=-0.35; p<0.001). In the final model, gender, age, governorate, parental education, body mass index, vitamin D supplement and number of times the adolescents walk to schools per week were all significantly related to vitamin D deficiency.

Conclusion

High prevalence of vitamin D deficiency was noted among adolescents in Kuwait despite the abundant sunshine which may reflect strong sun avoidance behavior. Adequate outdoor daytime activities should be encouraged especially for females. We call for locally tailored guidelines for vitamin D supplement in which females should have a higher dose compared to males.

Keywords: Vitamin D, adolescents, Kuwait, Middle East, school children

For peer review only

For peer review only - http://bmjopen.bmj.com/site/about/guidelines.xhtml

Strengths and limitations of this study

- This is the first study that aimed to estimate the prevalence of vitamin D on a large nationally representative sample of healthy adolescents in Kuwait.
- We measured vitamin D using the recommended laboratory methodology and gathered data from both parents and adolescents.
- We did not measure the skin color, which can be an important determinant for vitamin D synthesis in the skin.

For peer review only

For peer review only - http://bmjopen.bmj.com/site/about/guidelines.xhtml

Introduction

An optimal vitamin D status is extremely important during adolescence for proper growth and bone mineral accrual [1]. In addition to its critical role in bone health, several observational studies have linked vitamin D deficiency to various disease conditions during adolescence or adulthood such as asthma and allergies [2], type 2 diabetes [3], depression [4], cancer and even all-cause mortality [5]. However, the results of randomized control trials, which tested the impact of vitamin D supplement on the risk of these disease conditions, remain inconclusive. For example, there was no clear evidence from randomized controlled trials that vitamin D supplement reduces the risk of cancer [6], asthma [7], cardiovascular diseases [8], depression [9] or overall mortality [10].

Several studies have evaluated the prevalence of vitamin D deficiency and insufficiency during adolescence reporting high prevalence of vitamin D deficiency worldwide [11 12]. This was the case even in countries with abundant sunshine, which is the main factor for endogenous vitamin D synthesis. For example, in India the prevalence of vitamin D deficiency among adolescents was reported to be 85-98% [13 14] while in Saudi Arabia it was found to be around 96% [15]. In Arab states in the Gulf region or the broader Middle East, there is paucity of data on vitamin D status in pediatric and adolescents populations. The few studies that showed a high prevalence of vitamin D deficiency in adolescents suffered from major methodological weaknesses [15-17]. These include small sample size, lack of proper sampling technique or using less optimal laboratory method to measure vitamin D. It is also known that factors which influence vitamin D status such as avoiding sun exposure, skin pigmentation, high body mass index (BMI) and dietary factors, particularly the lack of vitamin D supplement and intake of vitamin D rich foods [18] are all on rise in Arab states in the Gulf region. The aim of the present study was therefore to assess vitamin D status among adolescents living in Kuwait, a country with a plenty of sun shine, and to investigate factors associated with low vitamin D level.

For peer review only - http://bmjopen.bmj.com/site/about/guidelines.xhtml

For peer review only

Methods

Study population and study participants

Kuwait is a small country at latitude (29.3759° N) with a population of 4.2 million. Approximately, 25% of population is under the age of 19-year. The study population comprised students in the age between 11 and 16 years in the public middle schools from all governorates of Kuwait. A school based cross-sectional study was conducted on students from 12 public middle schools that were selected using a stratified multistage cluster random sampling with a probability proportional to size. The sample allocation in each governorate was based on the relative size of that governorate as judged by the total number of students in the governorate. Students with major chronic disease conditions are registered in each school and these were excluded from the study. However, students with minor illnesses or self-reported illness were included in the study. The type of illness was recorded during data collection and included in the analysis. The study was funded by Kuwait University and approved by The Ethics Committee at The Ministry of Health in Kuwait (Ref. No. 2015/248) as well as The Ethics Committee at Health Sciences Centre, Kuwait University (Ref. No. DR/EC/2338).

Data Collection

Data were collected (including blood samples) in February, March and April, 2016. An informed consent was sent to the parents along with a self-administered questionnaire on parents' level of education (No formal education, primary/intermediate, high school, diploma, university and above), income, type of housing, number of siblings of the index child, passive smoking at household and the number of times per week the index child had a meal prepared outside home during the last three months. After obtaining written informed consent from the parents and verbal consent from the school children, trained dedicated personnel carried out face-to-face interviews with the students using a structured questionnaire. The questionnaire was carefully developed after extensive review of the literature and was pilot tested on 20 students who were not included in the study. It comprised questions on habitual sun exposure during the last three

For peer review only - http://bmjopen.bmj.com/site/about/guidelines.xhtml

months, which was assessed by the core questions developed to measure the exposure to sunlight in adolescents as described by Glanz et al. [19]. We also collected data on smoking habits, physical activity and dietary intake. Data on physical activity were collected using a questionnaire that was developed based on the Youth Physical Activity Questionnaire in UK [20] and The Arab teens lifestyle study [21]. The questionnaire was validated among high school students and showed strong correlation with data collected by accelerometers (Spearman correlation= 0.92; $p<0.001$ for total steps count) (not published). Data on dietary intake of vitamin D were collected from only 200 students using food frequency questionnaire for calcium and vitamin D intake in adolescents [22], which has been validated in our setting [23]. Food models or serving containers were used to assist in estimating serving size. Measurements of standing height and body weight of the study subjects were assessed using a digital weight and height scale (Detecto®) in a standardized manner.

Laboratory methods

Five ml of venous blood was collected in gel-containing tubes (SST II advance, BD vacutainer) from each participant by a trained nurse; and the samples were protected from light. On the same day, the samples were centrifuged at 2000 x g for 15 minutes and the serum was transferred to Eppendorf tubes and stored at -80°C until analysis. Serum 25-hydroxyvitamin D (25-OH-D) concentration is the best marker of vitamin D status because it is the major circulating form of vitamin D, reflecting both the amount produced in the skin after sun exposure and the amount consumed in foods [24]. It was measured in College of American Pathologists CAP-accredited laboratory by liquid chromatography-tandem mass spectrometry (LC-MS/MS), which is the recommended method for vitamin D assessment in epidemiological studies [25]. According to the Endocrine Society [26] and the Society for Adolescent Health and Medicine [27], we used the following cut-off of 25-OH-D to define vitamin D status: vitamin D deficiency < 50 nmol/L (20 ng/mL); vitamin D insufficiency 50-75 nmol/L (20-30 ng/mL); and vitamin D sufficiency≥ 75 nmol/L (30 ng/mL). Thus, hypovitaminosis D was defined in the presence of 25-OH-D levels < 75.0 nmol/L (30 ng/mL). Furthermore,

For peer review only - http://bmjopen.bmj.com/site/about/guidelines.xhtml

severe vitamin D deficiency was defined as 25-OH-D levels < 25.0 nmol/L (10 ng/mL) [28]. Serum intact Parathyroid hormone (PTH) was measured using the Access Intact PTH chemiluminescent immunoassay with the Unicel DxI 800 Beckman Coulter analyzer using commercial kit (Cat. # A16972). Similar to other studies, PTH level ≥ 65.0 ng/L was suggestive of hyperparathyroidism [29] although there is no consensus on this cut-off point.

Patient and Public Involvement

Public were not directly involved in the design of this study. However, schools were involved in approaching the parents to obtain their consents. We also sent the results of the laboratory tests of each participant to the parents through schools using closed envelops to ensure confidentiality.

Data analysis

Data were double entered into specifically designed database using Epidata Entry. Data analysis was conducted using Stata (StataCorp. 2011. Release 12). BMI was calculated as weight (Kg) divided by height squared (m^2). Weight status was categorized into normal, overweight and obese according to WHO growth charts. Chi-squared test was used to test differences in categorical variables. Because vitamin D deficiency was a common outcome and the log-binomial model failed to converge, a modified version of multiple logistic regression that calculates prevalence ratio was used to study the association between each presumed risk factor and vitamin D deficiency (25-OH-D < 50 nmol/L). Variables that were found to be statistically significant at 15% level of significance were considered in multivariate analysis. Variables were divided into groups (e.g. socio-demographic, sun exposure, etc) and were added sequentially to the model. Statistical significance of variables was evaluated by likelihood ratio test which compares the model with and without the variable. Goodness of fit of the final model was evaluated by Hosmer-Lemeshow test. Factors with p<0.05 were deemed to be statistically significant. The analysis above was repeated using hypovitaminosis (25-OH-D < 75.0 nmol/L) as the binary outcome. We also conducted multiple linear

For peer review only - http://bmjopen.bmj.com/site/about/guidelines.xhtml

regression analysis using vitamin D level as a continuous outcome (after log-transformation) and reported the results in the text. Because of the complex structure of this survey data, we used survey method, which gives more precise estimates of standard error.

Results

Of 1583 parents approached, 161 refused to participate (because children, parents or both refused). Another 6 samples were not sufficient to conduct blood analysis, thus the analysis below comprised 1416 students. Table (1) shows the socio-demographic characteristics of the study group. The mean (SD) age was 12.48 (0.94) years and 694 (49.01%) were males.

For peer review only - http://bmjopen.bmj.com/site/about/guidelines.xhtml

Table (1). Socio-demographic characteristics and vitamin D levels among adolescents in public middle schools in Kuwait.

Characteristics	Males n=694		Females n=722		Total N=1416	
**Age in years,** Mean (SD) years	12.56	(0.94)	12.41	(0.92)*	12.48	(0.94)
**25-OH-D nmol/L, Median (IQR)**	39.80	(29.4-52.7)	21.50	(14.7-30.7)*	29.70	(19.2-44.2)
	**n**	**(%)**	**n**	**(%)**	**n**	**(%)**
**Nationality** (N= 1416)						
Kuwaiti	423	(60.95)	658	(91.14)*	1,081	(76.34)
Non-Kuwait	271	(39.05)	64	(8.86)	335	(23.66)
**Father's Education** (N=1383)						
No formal education	9	(1.33)	6	(0.85)*	15	(1.08)
Primary/Intermediate	95	(14.05)	126	(17.82)	221	(15.98)
Secondary (high school)	157	(23.22)	187	(26.45)	344	(24.87)
Diploma	126	(18.64)	135	(19.09)	261	(18.87)
University & above	289	(42.75)	253	(35.79)	542	(39.19)
**Mother's Education** (N=1396)						
No formal education	18	(2.64)	13	(1.82)	31	(2.22)
Primary/Intermediate	79	(11.60)	73	(10.21)	152	(10.89)
Secondary (high school)	140	(20.56)	164	(22.94)	304	(21.78)
Diploma	136	(19.97)	168	(23.50)	304	(21.78)
University & above	308	(45.23)	297	(41.54)	605	(43.34)
**Father's Income** (Kuwaiti Dinars) (N=1370)						
Less than 500	64	(9.61)	27	(3.84)*	91	(6.64)
500 to 1000	168	(25.23)	136	(19.32)	304	(22.19)
1001 to 1500	175	(26.28)	246	(34.94)	421	(30.73)
1501 to 2000	100	(15.02)	119	(16.90)	219	(15.99)
More than 2000	83	(12.46)	90	(12.78)	173	(12.63)
Do not wish to tell	76	(11.41)	86	(12.22)	162	(11.82)
**Mother's Employment Status** (N=1388)						
Housewife	267	(39.38)	221	(31.13)*	488	(35.16)
Paid employment	307	(45.28)	373	(52.54)	680	(48.99)
Others	104	(15.34)	116	(16.34)	220	(15.85)
**Housing** (N=1397)						
Rented flat	301	(44.20)	209	(29.19)*	510	(36.51)
Rented house	94	(13.80)	69	(9.64)	163	(11.67)
Owned flat	17	(2.50)	42	(5.87)	59	(4.22)
Owned house	269	(39.50)	396	(55.31)	665	(47.60)

IQR: Interquartile Range. All %s are column percentages. * p<0.05 i.e. significant difference between males and females at 5% level of significance.

For peer review only - http://bmjopen.bmj.com/site/about/guidelines.xhtml

Prevalence of vitamin D deficiency

The median (Interquartile Range, IQR) of 25-OH-D was 29.7 (19.2- 44.2) nmol/L. Prevalence of vitamin D deficiency was 81.21% (95%CI: 71.61-90.81%) while severe deficiency was 39.48%. Only 3.60% of the participants were vitamin D sufficient. Prevalence of vitamin D deficiency was significantly higher among females compared to males (91.69% vs. 70.32%; $p<0.001$). Also, the median (IQR) of 25-OH-D was 39.8 (29.4- 52.7) nmol/L and 21.5 (14.7- 30.7) nmol/L among males and females, respectively ($p<0.001$). There was no difference in the prevalence of vitamin D deficiency between Kuwaitis and non-Kuwaitis after stratification by gender. There was significant inverse correlation between 25-OH-D and PTH (Spearman correlation=-0.35; $p<0.001$). In relation to vitamin D status, elevated PTH (secondary hyperparathyroidism PTH ≥65 ng/L. i.e. 6.89 pmol/L) was detected in 55.81%, 31.30% and 27.57% of adolescents with vitamin D severe deficiency, deficiency and insufficiency, respectively. If we used the reference range of the laboratory where the samples were tested (PTH: 1.3-9.3 pmol/L), the prevalence of secondary hyperparathyroidism would be 32.56%, 12.35%, and 11.68% among adolescents with vitamin D severe deficiency, deficiency and insufficiency, respectively. The median [IQR] PTH was significantly different dependent on vitamin D status (severe deficiency 7.37 [5.44-10.55] pmol/L; deficiency 5.63[4.13-7.40] pmol/L; insufficiency 5.14[3.87-6.99] pmol/L; sufficiency 4.31[3.75- 5.73] pmol/L; $p<0.001$). The actual relationship between 25-OH-D and PTH levels is shown in figure (1).

For peer review only - http://bmjopen.bmj.com/site/about/guidelines.xhtml

Table (2): Factors associated with vitamin D deficiency among adolescence in multivariate analysis.

Characteristics	Total	Prevalence of vitamin D deficiency n (%)	Prevalence Ratio [95%CI]	P
**Gender**				
Male	694	488 (70.32)	1 [Ref.]	<0.001
Female	722	662 (91.69)	1.20 [1.17- 1.21]	
**Age (year)**				
<12	527	409 (77.61)	1 [Ref.]	0.003
12-	439	369 (84.05)	1.10 [1.04- 1.14]	
≥13	450	372 (82.67)	1.07 [1.01- 1.12]	
**Governorate**				
Capital	156	127 (81.41)	1 [Ref.]	<0.001
Hawally	246	188 (76.42)	0.88 [0.73-1.01]	
Farawanya	236	183 (77.54)	0.85 [0.68-0.99]	
Jahra	239	214 (89.54)	1.10 [0.98-1.17]	
Mubarak al-Kabeer	148	124 (83.78)	1.06 [0.93-1.14]	
Ahmadi	371	296 (79.78)	1.06 [0.95-1.13]	
**Father's Education**				
Primary/Intermediate/no formal education	236	205 (86.86)	1 [Ref.]	<0.009
Secondary (high school)	344	297 (86.34)	1.04 [0.92-1.12]	
Diploma	261	220 (84.29)	1.01 [0.88-1.10]	
University & above	542	399 (73.62)	0.89 [0.76-1.01]	
**Passive smoking in household**				
No	901	704 (78.14)	1 [Ref.]	<0.006
Yes	489	426 (87.12)	1.08 [1.02-1.13]	
**Currently taking supplements**				
No	1,256	1,045 (83.20)	1 [Ref.]	<0.001
Yes	158	103 (65.19)	0.63 [0.49-0.76]	
**Consumption of sugary drinks per week**				
None	171	123 (71.93)	1 [Ref.]	0.002
Once-Three times	772	633 (81.99)	1.11 [1.05-1.16]	
Four-Six times	171	135 (78.95)	1.07 [0.96-1.14]	
Seven/more times	299	256 (85.62)	1.15 [1.08-1.19]	
**Number of times walking to/from school per week**				
None	1,158	982 (84.80)	1 [Ref.]	<0.001
1-8 times	155	99 (63.87)	0.81 [0.68-0.93]	
Every day	103	69 (66.99)	0.77 [0.61-0.92]	

For peer review only - http://bmjopen.bmj.com/site/about/guidelines.xhtml

**Body Mass Index Categories**[1]					
Normal weight	601	465 (77.37)	1	[Ref.]	0.001
Overweight	320	270 (84.38)	1.08	[1.01-1.13]	
Obese	471	400 (84.93)	1.12	[1.07-1.16]	
Underweight	24	15 (62.50)	0.93	[0.67-1.10]	

%s are row percentages. [1]according to WHO growth charts.

Factors associated with vitamin D status

Factors that showed significant association with vitamin D deficiency (25-OH-D < 50 nmol/L) in univariate analysis were gender, parental education, number of times per week the participants consumed breakfast or dinner prepared outside home, taking supplements, walking to/from school (instead of using school bus or car) in addition to BMI (Supplementary Table). Similarly, time spent outdoor per day (between 10:00 am and 4:00 pm) during weekdays and weekends in addition to wearing sunscreen and staying in shade or under an umbrella were all significant predictors in univariate analysis.

Table (2) shows the independent predictors for vitamin D deficiency in multivariate analysis. In the final model, gender, age, governorate, parental education, vitamin D supplement, and the number of times participant walks to/from school per week were all significantly related to vitamin D deficiency. Data from the food frequency questionnaire for calcium and vitamin D intake were available only for 200 study subjects, and of all food items, only eggs were significantly related to vitamin D deficiency in multivariate model (data not shown). We repeated the analysis above using hypovitaminosis (sufficient vs. insufficient/deficient) as a binary outcome. The same factors were found to be significantly related to hypovitaminosis, although mother's education (instead of father's education) was found to be significant in this analysis. We also used linear regression to identify the factors associated with vitamin D level as a continuous variable. Factors that showed significant association with vitamin D level in multivariate analysis were age, gender, governorate,

For peer review only - http://bmjopen.bmj.com/site/about/guidelines.xhtml

passive smoking at household, number of times per week the participants consumed breakfast prepared outside home in the last three months, time spent outdoor during weekdays, preference to stay in shade or under the umbrella, and BMI categories. With respect to dietary factors, milk and cheese, fast food hamburger and tuna fish were significantly associated with vitamin D level in univariate linear regression. When these food items were introduced to the final model, only milk and tuna fish were significantly associated with vitamin D level. It is worth noting that the best model was able to explain only 45% of the variability in vitamin D level in this analysis.

Discussion

Although several reports have described vitamin D deficiency among adults in the Arab states in the Gulf region, there are limited data on the prevalence of vitamin D deficiency among healthy adolescents. We used a nationally representative sample to examine vitamin D levels in a large number of healthy adolescents, becoming to our knowledge the largest study focusing on this group in Kuwait. We used the recommended method to measure vitamin D level and demonstrated that vitamin D deficiency is too common despite the abundant sunshine. Secondary hyperparathyroidism due to vitamin D deficiency is also common and females had lower vitamin D levels compared to males.

In our setting, approximately, 81% and 15% of adolescents were vitamin D deficient or insufficient, respectively. This is higher than that reported among adolescents in European countries [30-32], Southeastern United States [33 34] or New Zealand [35]. Our finding is more akin to that reported from Saudi Arabia (95.6%) [15], India (85-98%) [13 14], or Korea where only 2.3% of adolescents were found to be vitamin D sufficient [36]. Our findings are also in accordance with the high prevalence reported consistently among adult population in Kuwait [37] or other countries in the Gulf region such as Saudi Arabia [38], Bahrain [39] and Qatar [40].

For peer review only

For peer review only - http://bmjopen.bmj.com/site/about/guidelines.xhtml

Similar to other studies among adolescents [29 33 41], our findings showed that a large number of those with low vitamin D level had evidence of secondary hyperparathyroidism. We reported a much higher prevalence of secondary hyperparathyroidism compared to other studies [29 41], which could be due to using lower PTH cut-off point to define secondary hyperthyroidism or using different laboratory method to measure PTH. Hyperparathyroidism, which potentially leads to increased bone resorption, is particularly important during adolescence because it is the critical period to achieve peak bone mass. Serum PTH decreased as 25-OH-D level increased with no clear threshold of serum 25-OH-D at which PTH level plateaued although very few participants had elevated PTH when 25-OH-D level was above 75 nmol/L (Figure 1). Although we did not measure markers of bone turnover, this provides an additional evidence that 25-OH-D should be maintained above 75 nmol/L, which is the current cut-off point used to define vitamin D sufficiency in adolescents [26 27]. There were, however, adolescents with low vitamin D concentration without secondary hyperparathyroidism (Figure 1). This has been noted in other studies [33 41], the clinical significance of which is unknown in children and deserves further investigation.

Because of the abundant sunshine in the Arab states in the Gulf region, there has been a lot of skepticism of vitamin D deficiency. Informal discussions, even among scientific community, in the region show strong denial attributing such low levels of vitamin D to poorly executed or faulty laboratory measurements. Our findings should eliminate doubts on vitamin D deficiency among adolescents in Kuwait and pave the way to look for practical solutions. Factors that may hinder cutaneous synthesis of vitamin D despite abundant sunshine may include dust storms (which occur almost in one third of the year in Kuwait [42]), an avoidance of sun exposure by indoor lifestyle, head/body covering or other cultural practices and the use of sunscreen. Self-reported exposure to sunlight was a significant predictor for vitamin D deficiency in univariate analysis but lost statistical significance in multivariate analysis. Most of adolescents in our setting avoid sunlight as only around 6% of the participants spent more than two hours outdoor per day between

For peer review only - http://bmjopen.bmj.com/site/about/guidelines.xhtml

10:00 am and 4:00 pm during weekdays (data not shown). Avoidance of sunlight has been suggested to be the underlying reason for the counterintuitive seasonal variation that has been reported in the region, where the prevalence of vitamin D deficiency is higher in the summer compared to the winter season [41]. Owing to the desert climate, avoiding sun exposure would be stronger during summer due to extremely high temperature. We collected blood samples in February, March and April, thus we were unable to describe seasonal variation in vitamin D status. Exposure to sunlight contributes up to 90–95% of the vitamin D supply, while the number of foods naturally containing a significant quantity of vitamin D is very limited except for some oily fish that is rarely consumed by adolescents worldwide and in our setting [43] (~5% of adolescents in our setting consumed salmon once per week or more). As such, education of parents and adolescents on the safe amount of sun exposure in addition to changes in the school environment to facilitate exposure to sunlight may produce the highest impact on vitamin D status among adolescents. There is no consensus on the duration of time, which adolescents can safely be exposed to direct sunlight. However, exposure of legs and arms for at least 15 minutes twice per week has been reported to be sufficient for adequate sun-induced cutaneous vitamin D synthesis in adolescents [43].

Similar to other studies that investigated vitamin D deficiency among adolescents, females had a significantly higher prevalence of vitamin D deficiency compared to males even after adjusting for all potential confounders. Also, vitamin D level was significantly lower among females compared to males. Similar findings have been reported among adolescents from Saudi Arabia [44], India [14], Korea [36] and Taiwan [45]. This pattern is not common in other settings as reported from a clinic-based cross-sectional study in the US [33] or Italy [31]. In the Arab states in the Gulf region and India, cultural practices, such as type of clothing that covers the whole body and indoor lifestyle, have been proposed to exacerbate vitamin D deficiency among females. Indeed, we found males consistently reporting higher amount of outdoor exposure to sunlight compared to females. In addition, females reported consistently higher sun avoidance behavior such as using sunscreen and staying in shade or under an umbrella. Adjusting for self-reported

For peer review only - http://bmjopen.bmj.com/site/about/guidelines.xhtml

exposure to sunlight did not explain the whole association between vitamin D status and gender, which could be due to residual confounding. We also collected data on the type of clothing among females using photo cards (no head covering, head covering, head and face covering), but vitamin D deficiency was too common among females, which did not allow for us to investigate the impact of type of dress on vitamin D status among females. In our setting, efforts to improve vitamin D level among adolescents should focus on females through encouragement of safe amount of exposure to sunlight and/or intake of vitamin D rich foods. Also, the current guidelines for vitamin D supplement should take the difference between females and males into account and recommend higher doses for females compared to males.

Most of the students in Kuwait do not walk to schools and are mainly transported by cars or school buses. The number of times student walk to/from school as well as the time spent on walking to/from school was significantly associated with vitamin D deficiency. Those who walk to/from schools were less likely to have vitamin D deficiency compared to those who used school bus or car. Walking to/from schools can be a good opportunity for sunlight exposure as well as increasing physical activity, which itself may improve vitamin D level. In a study in the US, physical activity was inversely associated with hypovitaminosis D but not related to vitamin D level [33] while in another study, vigorous physical activity was significantly associated with vitamin D level [34]. It has been proposed that physical activity may increase the level of vitamin D through increasing the time spent outdoor in sunlight or through reducing the risk of obesity [18]. However, in our study the total time spent on other physical/sport activities was significantly associated with vitamin D deficiency in univariate but not in multivariate analysis. Unlike walking to/from schools, most sport activities in our setting are practiced indoor or during night time due to severe hot weather which may explain the lack of association between total time spent on physical activities and vitamin D deficiency in multivariate analysis. Like many other studies [30 33 36], BMI was inversely associated with vitamin D level, which has been attributed to the sequestration of vitamin D within the plentiful adipose tissue [46 47]. It has also been

For peer review only - http://bmjopen.bmj.com/site/about/guidelines.xhtml

suggested that leptin, an adipocyte-derived hormone, might activate a pathway that inhibits renal synthesis of the active form of vitamin D [48].

In our study, sugary drinks were positively associated with the prevalence of vitamin D deficiency. This is similar to that reported from Saudi Arabia [41] and US [33]. Consumption of sugary drinks among adolescents usually occurs at the expense of milk consumption which contains vitamin D [49]. As a result, it has been proposed that the availability of vitamin D-fortified juices may help reduce vitamin D deficiency in adolescents [33]. In our data those who consumed sugary drinks were less likely to consume milk (data not shown). Although milk consumption was not related to vitamin D deficiency it was significantly related to the vitamin D level in linear regression analysis.

We found an association between passive smoking at household (reported by parents) and vitamin D deficiency among adolescents, which is interesting and has been reported before [45]. Only less than 1% of the adolescents in our setting reported active smoking. Smoking has also been reported to be a significant determinant for low vitamin D levels among adults in some studies [32] but not in others [50] and this issue remains controversial [32]. Furthermore, a recent study found no significant association between cotinine level and vitamin D deficiency among Korean adolescents [36]. One of the hypothesized mechanism is that smoking may reflect an overall less healthy life style including less physical activity and poor dietary habits [32]. A causal link has also been proposed as metabolites in cigarette's smoke can inhibit CYP27A1 activity [51], which is involved in vitamin D metabolism.

Strengths and limitation

This is the first study that measured vitamin D in a large nationally representative sample of adolescents in Kuwait. We used a measurement method that is recommended in epidemiological studies and gathered data from both parents and adolescents. However, we did not measure the skin color, which can be an

For peer review only - http://bmjopen.bmj.com/site/about/guidelines.xhtml

important determinant for vitamin D synthesis in the skin during exposure to sunlight. We evaluated the dietary intake of vitamin D in only a subgroup of study participants. However, dietary intake of vitamin D is known to have a smaller contribution to vitamin D status compared to cutaneous synthesis in response to sun exposure [43] and data on vitamin D supplement were obtained from all participants.

Conclusion

In conclusion, a high prevalence of vitamin D deficiency was noted among adolescents in Kuwait despite the abundant sunshine. This may reflect strong sun avoidance behavior as only less than 4% of adolescents were vitamin D sufficient and most of them were on vitamin D supplements. An optimal vitamin D level is essential during adolescence; and vitamin D deficiency at this age represents a public health problem that should be addressed. First, our findings should help clear doubts on vitamin D deficiency among adolescents in Kuwait, which is a prerequisite for prevention initiatives. Second, sun exposure is the main source for vitamin D in this age group; therefore adequate outdoor daytime activities should be encouraged especially for females. Slight modifications in school environment to facilitate exposure to sunlight during school breaks between classes can be fruitful. With respect to vitamin D supplementation, we call for locally tailored guidelines in which females should have a higher amount of vitamin D supplement compared to males. Vitamin D fortification particularly for food products that are popular for adolescents in our setting should also be considered. Increasing exposure to sunlight and food fortification with vitamin D should not be considered mutually exclusive and both strategies can be adopted.

For peer review only

Acknowledgements

The authors acknowledge the assistance of Nadien Sameeh Rushdi and the team of data collectors. We also acknowledge the cooperation of all participating schools and the facilitation of the project by the Ministry of Education. We also appreciate the support and cooperation of the staff and management of The United Genetics Laboratories (Kuwait) in vitamin D analysis.

For peer review only - http://bmjopen.bmj.com/site/about/guidelines.xhtml

Authors' contributions

AT contributed to the study design and data collection, analyzed the data and drafted the paper. He had full access to all the data and took responsibility for the integrity of the data. AR contributed to the design of the study and data collection in addition to the writing and revising the manuscript. RS and LS contributed to the design of the study and data collection in addition to revising the manuscript with significant intellectual input. AH contributed to the data collection and revised the manuscript.

Funding: The work was supported and funded by Kuwait University- Research Project No. WF 02/13.

Competing interests: The authors declare that they have no competing interests

Data sharing statement: No additional data are available.

For peer review only

For peer review only - http://bmjopen.bmj.com/site/about/guidelines.xhtml

References

1. Mouratidou T, Vicente-Rodriguez G, Gracia-Marco L, et al. Associations of dietary calcium, vitamin D, milk intakes, and 25-hydroxyvitamin D with bone mass in Spanish adolescents: the HELENA study. Journal of clinical densitometry : the official journal of the International Society for Clinical Densitometry 2013;**16**(1):110-7 doi: 10.1016/j.jocd.2012.07.008[published Online First: Epub Date]|.

2. Sharief S, Jariwala S, Kumar J, Muntner P, Melamed ML. Vitamin D levels and food and environmental allergies in the United States: results from the National Health and Nutrition Examination Survey 2005-2006. The Journal of allergy and clinical immunology 2011;**127**(5):1195-202 doi: 10.1016/j.jaci.2011.01.017[published Online First: Epub Date]|.

3. Lucato P, Solmi M, Maggi S, et al. Low vitamin D levels increase the risk of type 2 diabetes in older adults: A systematic review and meta-analysis. Maturitas 2017;**100**:8-15 doi: 10.1016/j.maturitas.2017.02.016[published Online First: Epub Date]|.

4. Bahrami A, Mazloum SR, Maghsoudi S, et al. High Dose Vitamin D Supplementation Is Associated With a Reduction in Depression Score Among Adolescent Girls: A Nine-Week Follow-Up Study. Journal of dietary supplements 2017:1-10 doi: 10.1080/19390211.2017.1334736[published Online First: Epub Date]|.

5. Chowdhury R, Kunutsor S, Vitezova A, et al. Vitamin D and risk of cause specific death: systematic review and meta-analysis of observational cohort and randomised intervention studies. BMJ (Clinical research ed.) 2014;**348**:g1903 doi: 10.1136/bmj.g1903[published Online First: Epub Date]|.

6. Bjelakovic G, Gluud LL, Nikolova D, et al. Vitamin D supplementation for prevention of cancer in adults. The Cochrane database of systematic reviews 2014(6):CD007469 doi: 10.1002/14651858.CD007469.pub2[published Online First: Epub Date]|.

7. Riverin BD, Maguire JL, Li P. Vitamin D Supplementation for Childhood Asthma: A Systematic Review and Meta-Analysis. PloS one 2015;**10**(8):e0136841 doi: 10.1371/journal.pone.0136841[published Online First: Epub Date]|.

8. Swart KM, Lips P, Brouwer IA, et al. Effects of vitamin D supplementation on markers for cardiovascular disease and type 2 diabetes: an individual participant data meta-analysis of randomized controlled trials. The American journal of clinical nutrition 2018;**107**(6):1043-53 doi: 10.1093/ajcn/nqy078[published Online First: Epub Date]|.

9. Shaffer JA, Edmondson D, Wasson LT, et al. Vitamin D supplementation for depressive symptoms: a systematic review and meta-analysis of randomized controlled trials. Psychosomatic medicine 2014;**76**(3):190-6 doi: 10.1097/psy.0000000000000044[published Online First: Epub Date]|.

10. Bjelakovic G, Gluud LL, Nikolova D, et al. Vitamin D supplementation for prevention of mortality in adults. The Cochrane database of systematic reviews 2014(1):CD007470 doi: 10.1002/14651858.CD007470.pub3[published Online First: Epub Date]|.

11. Hilger J, Friedel A, Herr R, et al. A systematic review of vitamin D status in populations worldwide. The British journal of nutrition 2014;**111**(1):23-45 doi: 10.1017/s0007114513001840[published Online First: Epub Date]|.

12. Basatemur E, Horsfall L, Marston L, Rait G, Sutcliffe A. Trends in the Diagnosis of Vitamin D Deficiency. Pediatrics 2017;**139**(3) doi: 10.1542/peds.2016-2748[published Online First: Epub Date]|.

13. Garg MK, Marwaha RK, Khadgawat R, et al. Efficacy of vitamin D loading doses on serum 25-hydroxy vitamin D levels in school going adolescents: an open label non-randomized prospective trial. Journal of pediatric endocrinology & metabolism : JPEM 2013;**26**(5-6):515-23 doi: 10.1515/jpem-2012-0390[published Online First: Epub Date]|.

For peer review only - http://bmjopen.bmj.com/site/about/guidelines.xhtml

14. Kapil U, Pandey RM, Goswami R, et al. Prevalence of Vitamin D deficiency and associated risk factors among children residing at high altitude in Shimla district, Himachal Pradesh, India. Indian journal of endocrinology and metabolism 2017;**21**(1):178-83 doi: 10.4103/2230-8210.196031[published Online First: Epub Date]|.

15. AlBuhairan FS, Tamim H, Al Dubayee M, et al. Time for an Adolescent Health Surveillance System in Saudi Arabia: Findings From "Jeeluna". The Journal of adolescent health : official publication of the Society for Adolescent Medicine 2015;**57**(3):263-9 doi: 10.1016/j.jadohealth.2015.06.009[published Online First: Epub Date]|.

16. Kaddam IM, Al-Shaikh AM, Abaalkhail BA, et al. Prevalence of vitamin D deficiency and its associated factors in three regions of Saudi Arabia. Saudi medical journal 2017;**38**(4):381-90 doi: 10.15537/smj.2017.4.18753[published Online First: Epub Date]|.

17. Mansour MM, Alhadidi KM. Vitamin D deficiency in children living in Jeddah, Saudi Arabia. Indian journal of endocrinology and metabolism 2012;**16**(2):263-9 doi: 10.4103/2230-8210.93746[published Online First: Epub Date]|.

18. Valtuena J, Gonzalez-Gross M, Huybrechts I, et al. Factors associated with vitamin D deficiency in European adolescents: the HELENA study. Journal of nutritional science and vitaminology 2013;**59**(3):161-71

19. Glanz K, Yaroch AL, Dancel M, et al. Measures of sun exposure and sun protection practices for behavioral and epidemiologic research. Archives of dermatology 2008;**144**(2):217-22 doi: 10.1001/archdermatol.2007.46[published Online First: Epub Date]|.

20. UEA. Youth Physical Activity Questionnaire. Secondary Youth Physical Activity Questionnaire 2011. http://epi-meta.medschl.cam.ac.uk/includes/speedy/pdf/PhysicalActivity%20V4%20SPEEDY-3.pdf.

21. Al-Hazzaa HM, Al-Sobayel HI, Musaiger AO. Convergent validity of the Arab Teens Lifestyle Study (ATLS) physical activity questionnaire. International journal of environmental research and public health 2011;**8**(9):3810-20 doi: 10.3390/ijerph8093810[published Online First: Epub Date]|.

22. Taylor C, Lamparello B, Kruczek K, Anderson EJ, Hubbard J, Misra M. Validation of a food frequency questionnaire for determining calcium and vitamin D intake by adolescent girls with anorexia nervosa. Journal of the American Dietetic Association 2009;**109**(3):479-85, 85 e1-3 doi: 10.1016/j.jada.2008.11.025[published Online First: Epub Date]|.

23. Papandreou D, achaniotis N, Lari M, Al Mussabi W. Validation of a Food Frequency Questionnaire for Vitamin D and Calcium Intake in Healthy Female College Students. Food and Nutrition Sciences 2014;**5**:2048-52

24. Holick MF. Vitamin D deficiency. The New England journal of medicine 2007;**357**(3):266-81 doi: 10.1056/NEJMra070553[published Online First: Epub Date]|.

25. Vogeser M. Quantification of circulating 25-hydroxyvitamin D by liquid chromatography-tandem mass spectrometry. The Journal of steroid biochemistry and molecular biology 2010;**121**(3-5):565-73 doi: 10.1016/j.jsbmb.2010.02.025[published Online First: Epub Date]|.

26. Holick MF, Binkley NC, Bischoff-Ferrari HA, et al. Evaluation, treatment, and prevention of vitamin D deficiency: an Endocrine Society clinical practice guideline. The Journal of clinical endocrinology and metabolism 2011;**96**(7):1911-30 doi: 10.1210/jc.2011-0385[published Online First: Epub Date]|.

27. Medicine SfAHa. Recommended vitamin D intake and management of low vitamin D status in adolescents: a position statement of the society for adolescent health and medicine. The Journal of adolescent health : official publication of the Society for Adolescent Medicine 2013;**52**(6):801-3 doi: 10.1016/j.jadohealth.2013.03.022[published Online First: Epub Date]|.

For peer review only - http://bmjopen.bmj.com/site/about/guidelines.xhtml

28. Braegger C, Campoy C, Colomb V, et al. Vitamin D in the healthy European paediatric population. Journal of pediatric gastroenterology and nutrition 2013;**56**(6):692-701 doi: 10.1097/MPG.0b013e31828f3c05[published Online First: Epub Date]|.

29. Vierucci F, Del Pistoia M, Fanos M, Erba P, Saggese G. Prevalence of hypovitaminosis D and predictors of vitamin D status in Italian healthy adolescents. Italian journal of pediatrics 2014;**40**:54 doi: 10.1186/1824-7288-40-54[published Online First: Epub Date]|.

30. Gonzalez-Gross M, Valtuena J, Breidenassel C, et al. Vitamin D status among adolescents in Europe: the Healthy Lifestyle in Europe by Nutrition in Adolescence study. The British journal of nutrition 2012;**107**(5):755-64 doi: 10.1017/s0007114511003527[published Online First: Epub Date]|.

31. Vierucci F, Del Pistoia M, Fanos M, et al. Vitamin D status and predictors of hypovitaminosis D in Italian children and adolescents: a cross-sectional study. European journal of pediatrics 2013;**172**(12):1607-17 doi: 10.1007/s00431-013-2119-z[published Online First: Epub Date]|.

32. Kassi EN, Stavropoulos S, Kokkoris P, et al. Smoking is a significant determinant of low serum vitamin D in young and middle-aged healthy males. Hormones (Athens, Greece) 2015;**14**(2):245-50 doi: 10.14310/horm.2002.1521[published Online First: Epub Date]|.

33. Gordon CM, DePeter KC, Feldman HA, Grace E, Emans SJ. Prevalence of vitamin D deficiency among healthy adolescents. Archives of pediatrics & adolescent medicine 2004;**158**(6):531-7 doi: 10.1001/archpedi.158.6.531[published Online First: Epub Date]|.

34. Dong Y, Pollock N, Stallmann-Jorgensen IS, et al. Low 25-hydroxyvitamin D levels in adolescents: race, season, adiposity, physical activity, and fitness. Pediatrics 2010;**125**(6):1104-11 doi: 10.1542/peds.2009-2055[published Online First: Epub Date]|.

35. Rockell JE, Green TJ, Skeaff CM, et al. Season and ethnicity are determinants of serum 25-hydroxyvitamin D concentrations in New Zealand children aged 5-14 y. The Journal of nutrition 2005;**135**(11):2602-8

36. Byun EJ, Heo J, Cho SH, Lee JD, Kim HS. Suboptimal vitamin D status in Korean adolescents: a nationwide study on its prevalence, risk factors including cotinine-verified smoking status and association with atopic dermatitis and asthma. BMJ open 2017;**7**(7):e016409 doi: 10.1136/bmjopen-2017-016409[published Online First: Epub Date]|.

37. Zhang FF, Al Hooti S, Al Zenki S, et al. Vitamin D deficiency is associated with high prevalence of diabetes in Kuwaiti adults: results from a national survey. BMC public health 2016;**16**:100 doi: 10.1186/s12889-016-2758-x[published Online First: Epub Date]|.

38. Al-Daghri NM. Vitamin D in Saudi Arabia: Prevalence,distribution and disease associations. The Journal of steroid biochemistry and molecular biology 2016 doi: 10.1016/j.jsbmb.2016.12.017[published Online First: Epub Date]|.

39. Golbahar J, Al-Saffar N, Altayab Diab D, Al-Othman S, Darwish A, Al-Kafaji G. Predictors of vitamin D deficiency and insufficiency in adult Bahrainis: a cross-sectional study. Public health nutrition 2014;**17**(4):732-8 doi: 10.1017/s136898001300030x[published Online First: Epub Date]|.

40. Mahdy S, Al-Emadi SA, Khanjar IA, et al. Vitamin D status in health care professionals in Qatar. Saudi medical journal 2010;**31**(1):74-7

41. Sulimani RA, Mohammed AG, Alfadda AA, et al. Vitamin D deficiency and biochemical variations among urban Saudi adolescent girls according to season. Saudi medical journal 2016;**37**(9):1002-8 doi: 10.15537/smj.2016.9.15248[published Online First: Epub Date]|.

For peer review only - http://bmjopen.bmj.com/site/about/guidelines.xhtml

42. Thalib L, Al-Taiar A. Dust storms and the risk of asthma admissions to hospitals in Kuwait. The Science of the total environment 2012;**433**:347-51 doi: 10.1016/j.scitotenv.2012.06.082[published Online First: Epub Date]|.

43. Holick MF. The D-lightful vitamin D for child health. JPEN. Journal of parenteral and enteral nutrition 2012;**36**(1 Suppl):9S-19S doi: 10.1177/0148607111430189[published Online First: Epub Date]|.

44. Al-Daghri NM, Al-Saleh Y, Aljohani N, et al. Vitamin D Deficiency and Cardiometabolic Risks: A Juxtaposition of Arab Adolescents and Adults. PloS one 2015;**10**(7):e0131315 doi: 10.1371/journal.pone.0131315[published Online First: Epub Date]|.

45. Yao TC, Tu YL, Chang SW, et al. Suboptimal vitamin D status in a population-based study of Asian children: prevalence and relation to allergic diseases and atopy. PloS one 2014;**9**(6):e99105 doi: 10.1371/journal.pone.0099105[published Online First: Epub Date]|.

46. Wortsman J, Matsuoka LY, Chen TC, Lu Z, Holick MF. Decreased bioavailability of vitamin D in obesity. The American journal of clinical nutrition 2000;**72**(3):690-3

47. Walker GE, Ricotti R, Roccio M, et al. Pediatric obesity and vitamin D deficiency: a proteomic approach identifies multimeric adiponectin as a key link between these conditions. PloS one 2014;**9**(1):e83685 doi: 10.1371/journal.pone.0083685[published Online First: Epub Date]|.

48. Tsuji K, Maeda T, Kawane T, Matsunuma A, Horiuchi N. Leptin stimulates fibroblast growth factor 23 expression in bone and suppresses renal 1alpha,25-dihydroxyvitamin D3 synthesis in leptin-deficient mice. J Bone Miner Res 2010;**25**(8):1711-23 doi: 10.1002/jbmr.65[published Online First: Epub Date]|.

49. O'Connor TM, Yang SJ, Nicklas TA. Beverage intake among preschool children and its effect on weight status. Pediatrics 2006;**118**(4):e1010-8 doi: 10.1542/peds.2005-2348[published Online First: Epub Date]|.

50. Scragg R, Holdaway I, Jackson R, Lim T. Plasma 25-hydroxyvitamin D3 and its relation to physical activity and other heart disease risk factors in the general population. Annals of epidemiology 1992;**2**(5):697-703

51. Aboraia AS, Makowski B, Bahja A, et al. Synthesis and CYP24A1 inhibitory activity of (E)-2-(2-substituted benzylidene)- and 2-(2-substituted benzyl)-6-methoxy-tetralones. European journal of medicinal chemistry 2010;**45**(10):4427-34 doi: 10.1016/j.ejmech.2010.07.001[published Online First: Epub Date]|.

For peer review only

For peer review only - http://bmjopen.bmj.com/site/about/guidelines.xhtml

Figure legends

Figure (1): Parathyroid hormone (PTH) with Serum 25-hydroxyvitamin D (25-OH-D) concentration. Individuals above the horizontal line (PTH ≥ 65.0 ng/L) are adolescents with secondary hyperthyroidism. Vertical lines represent the 25-OH-D cut-off used to define vitamin D deficiency, insufficiency and sufficiency (seven participants with 25-OH-D>150 nmol/L were omitted).

For peer review only

For peer review only - http://bmjopen.bmj.com/site/about/guidelines.xhtml

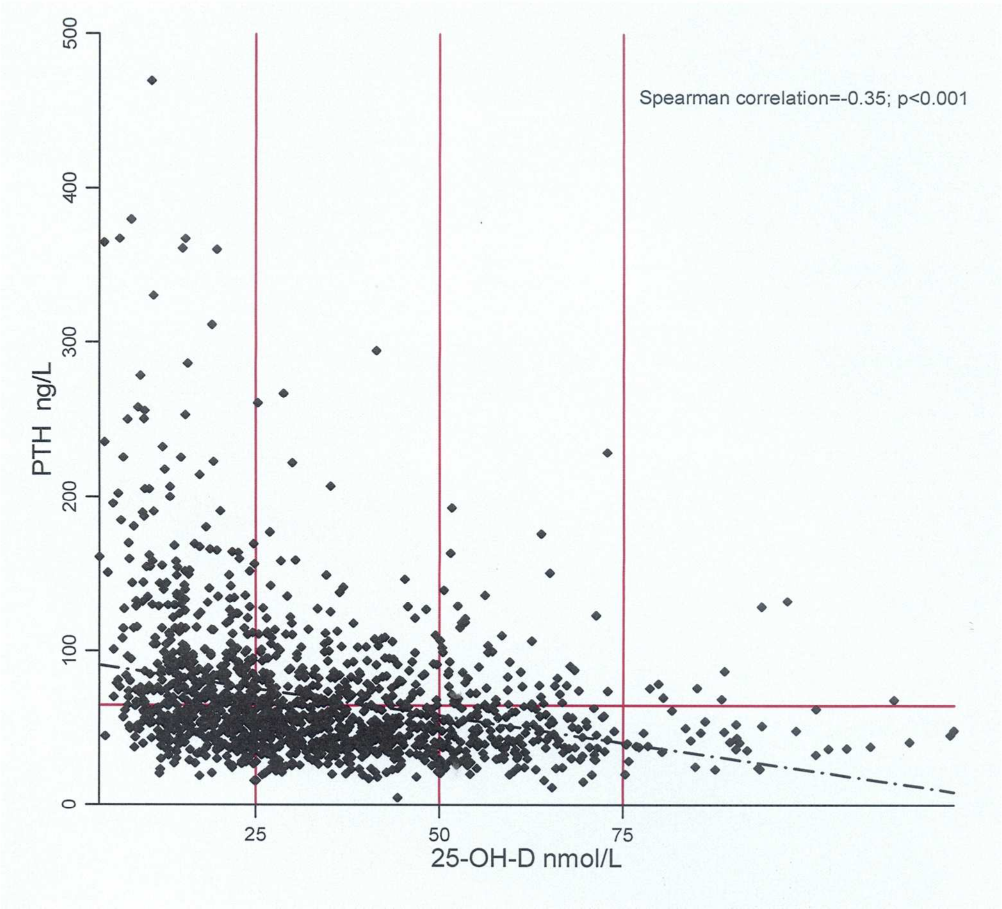

Figure (1): Parathyroid hormone (PTH) with Serum 25-hydroxyvitamin D (25-OH-D) concentration. Individuals above the horizontal line (PTH ≥ 65.0 ng/L) are adolescents with secondary hyperthyroidism. Vertical lines represent the 25-OH-D cut-off used to define vitamin D deficiency, insufficiency and sufficiency (seven participants with 25-OH-D>150 nmol/L were omitted).

138x127mm (300 x 300 DPI)

Supplementary Table (1). Factors associated with vitamin D deficiency among adolescents in univariate analysis.

Characteristics	Total	Prevalence of vitamin D deficiency n=1150	(%)	Prevalence Ratio [95%CI]		p
**Gender**						
Male	694	488	(70.32)	1	[Ref.]	<0.001
Female	722	662	(91.69)	1.30	[1.26- 1.33]	
**Age (year)**						
<12	527	409	(77.61)	1	[Ref.]	0.024
12-	439	369	(84.05)	1.07	[1.01- 1.12]	
≥13	450	372	(82.67)	1.06	[1.00- 1.10]	
**Nationality**						
Kuwaiti	1081	900	(83.26)	1	[Ref.]	<0.001
Non-Kuwait	335	250	(74.63)	0.90	[0.82-0.96]	
**Governorate**						
Capital	156	127	(81.41)	1	[Ref.]	0.003
Hawally	246	188	(76.42)	0.94	[0.82-1.03]	
Farawanya	236	183	(77.54)	0.95	[0.83-1.04]	
Jahra	239	214	(89.54)	1.10	[1.02-1.15]	
Mubarak al-Kabeer	148	124	(83.78)	1.03	[0.91-1.10]	
Ahmadi	371	296	(79.78)	0.98	[0.88-1.06]	
**Father's Education**						
Primary/Intermediate/no formal education	236	205	(86.86)	1	[Ref.]	<0.001
Secondary (high school)	344	297	(86.34)	0.99	[0.87-1.08]	
Diploma	261	220	(84.29)	0.95	[0.82-1.06]	
University & above	542	399	(73.62)	0.78	[0.65-0.90]	
**Mother's Education**						
Primary/Intermediate/no formal education	183	153	(83.61)	1	[Ref.]	<0.001
Secondary (high school)	304	263	(86.51)	1.04	[0.94-1.12]	
Diploma	304	259	(85.20)	1.02	[0.91-1.10]	
University & above	605	457	(75.54)	0.88	[0.76-0.99]	
**Total number of brother/sisters**						
Zero-two	298	223	(74.83)	1	[Ref.]	0.002
Three-four	538	434	(80.67)	1.05	[1.00-1.10]	
Five or more	553	469	(84.81)	1.10	[1.04-1.13]	
**Passive smoking at household**						
No	901	704	(78.14)	1	[Ref.]	<0.001
Yes	489	426	(87.12)	1.11	[1.06-1.15]	

Times per week consumed breakfast not prepared at home							
Zero	624	484	(77.56)	1	[Ref.]	0.019
One-two times	569	478	(84.01)	1.08	[1.02-1.12]	
Three-four	117	98	(83.76)	1.07	[0.97-1.14]	
Five or more	78	67	(85.90)	1.10	[1.00-1.17]	
**Times per week consumed dinner not prepared at home**						
Zero	157	114	(72.61)	1	[Ref.]	0.018
One-two times	878	719	(81.89)	1.09	[1.03-1.14]	
Three-four	257	211	(82.10)	1.09	[1.01-1.15]	
Five or more	86	75	(87.21)	1.14	[1.04-1.19]	
**Consumption of sugary drinks per week**						
None	171	123	(71.93)	1	[Ref.]	0.002
Once-Three times	772	633	(81.99)	1.09	[1.04-1.14]	
Four-Six	171	135	(78.95)	1.07	[0.98-1.13]	
Seven/more	299	256	(85.62)	1.12	[1.07-1.17]	
**Currently taking supplements**						
No	1,256	1,045	(83.20)	1	[Ref.]	<0.001
Yes	158	103	(65.19)	0.78	[0.68-0.88]	
**Number of times walking to school per week**						
None	1,158	982	(84.80)	1	[Ref.]	<0.001
1-8 times	155	99	(63.87)	0.74	[0.63-0.83]	
Every day	103	69	(66.99)	0.77	[0.65-0.89]	
**Time walking to school**						
None	1,158	982	(84.80)	1	[Ref.]	<0.001
≤5 minutes	80	50	(62.50)	0.71	[0.56-0.84]	
6- 10 minutes	76	53	(69.74)	0.80	[0.65-0.93]	
11-15	56	38	(67.86)	0.77	[0.60-0.92]	
16 minutes or more	45	26	(57.78)	0.65	[0.46-0.82]	
**Time spent on physical activity per week**						
Low (lower tertile)	469	396	(84.43)	1	[Ref.]	0.005
Medium (middle tertile)	473	391	(82.66)	0.97	[0.89-1.04]	
High (higher tertile)	474	363	(76.58)	0.89	[0.80-0.96]	
**Body Mass Index Categories[1]**						
Normal weight	601	465	(77.37)	1	[Ref.]	0.001
Overweight	320	270	(84.38)	1.07	[1.02-1.11]	

Obese	471	400 (84.93)	1.08	[1.03-1.11]	
Under weight	24	15 (62.50)	0.84	[0.59-1.02]	
**Time outside per day 10:00 am- 4:00 pm week days**					
Less than 30 minutes	657	563 (85.69)	1	[Ref.]	<0.001
31 minutes to 1 hour	450	342 (76.00)	0.87	[0.80-0.94]	
More than 1 hour to 2 hours	222	178 (80.18)	0.93	[0.84-1.00]	
More than 2 hours	87	67 (77.01)	0.88	[0.74-0.99]	
**Time outside per day 10:00 am- 4:00 pm week ends**					
Less than 30 minutes	494	425 (86.03)	1	[Ref.]	0.003
31 minutes to 1 hour	312	257 (82.37)	0.94	[0.85-1.02]	
More than 1 hour to 2 hours	255	198 (77.65)	0.87	[0.76-0.96]	
More than 2 hours to 3 hours	185	142 (76.76)	0.85	[0.74-0.96]	
More than 3 hours	169	127 (75.15)	0.83	[0.71-0.94]	
**Wearing sunscreen**					
Never	1,100	874 (79.45)	1	[Ref.]	0.013
Rarely	77	65 (84.42)	1.06	[0.94-1.13]	
Sometimes	160	140 (87.50)	1.09	[1.02-1.14]	
Often/always	79	71 (89.87)	1.12	[1.02-1.18]	
**Staying in shade or under an umbrella**					
Never	493	396 (80.32)	1	[Ref.]	0.04
Rarely	97	70 (72.16)	0.90	[0.78-1.01]	
Sometimes	363	301 (82.92)	1.03	[0.96-1.08]	
Often	205	162 (79.02)	0.98	[0.90-1.05]	
Always	257	220 (85.60)	1.06	[0.99-1.11]	

%s are row percentages. [1]according to WHO growth charts.

STROBE 2007 (v4) checklist of items to be included in reports of observational studies in epidemiology*
Checklist for cohort, case-control, and cross-sectional studies (combined)

Section/Topic	Item #	Recommendation	Reported on page #
**Title and abstract**	1	(*a*) Indicate the study's design with a commonly used term in the title or the abstract	Page 1
		(*b*) Provide in the abstract an informative and balanced summary of what was done and what was found	Page 3
**Introduction**			
Background/rationale	2	Explain the scientific background and rationale for the investigation being reported	Page 5
Objectives	3	State specific objectives, including any pre-specified hypotheses	Page 5
**Methods**			
Study design	4	Present key elements of study design early in the paper	Page 6
Setting	5	Describe the setting, locations, and relevant dates, including periods of recruitment, exposure, follow-up, and data collection	Page 6- Page 8 and Page 14
Participants	6	(*a*) *Cohort study*—Give the eligibility criteria, and the sources and methods of selection of participants. Describe methods of follow-up<br>*Case-control study*—Give the eligibility criteria, and the sources and methods of case ascertainment and control selection. Give the rationale for the choice of cases and controls<br>*Cross-sectional study*—Give the eligibility criteria, and the sources and methods of selection of participants	Page 6
		(*b*) *Cohort study*—For matched studies, give matching criteria and number of exposed and unexposed<br>*Case-control study*—For matched studies, give matching criteria and the number of controls per case	
Variables	7	Clearly define all outcomes, exposures, predictors, potential confounders, and effect modifiers. Give diagnostic criteria, if applicable	Page 6 and Page 7
Data sources/ measurement	8*	For each variable of interest, give sources of data and details of methods of assessment (measurement). Describe comparability of assessment methods if there is more than one group	*Page 6 and Page 7*
Bias	9	Describe any efforts to address potential sources of bias	Page 6, Page 12 and Page 17
Study size	10	Explain how the study size was arrived at	Page 13
Quantitative variables	11	Explain how quantitative variables were handled in the analyses. If applicable, describe which groupings were chosen and why	Page 7 and Page 8
Statistical methods	12	(*a*) Describe all statistical methods, including those used to control for confounding	Page 7 and Page 8
		(*b*) Describe any methods used to examine subgroups and interactions	Page 7 and Page 8
		(*c*) Explain how missing data were addressed	These were minimum.

For peer review only

		(*d*) *Cohort study*—If applicable, explain how loss to follow-up was addressed<br>*Case-control study*—If applicable, explain how matching of cases and controls was addressed<br>*Cross-sectional study*—If applicable, describe analytical methods taking account of sampling strategy	Page 8
		(*e*) Describe any sensitivity analyses	
**Results**			
Participants	13*	(a) Report numbers of individuals at each stage of study—eg numbers potentially eligible, examined for eligibility, confirmed eligible, included in the study, completing follow-up, and analysed	Page 8
		(b) Give reasons for non-participation at each stage	Page 8
		(c) Consider use of a flow diagram	
Descriptive data	14*	(a) Give characteristics of study participants (eg demographic, clinical, social) and information on exposures and potential confounders	Table 1 page 9
		(b) Indicate number of participants with missing data for each variable of interest	Table 1 , Table 2 and also supplementary table
		(c) *Cohort study*—Summarise follow-up time (eg, average and total amount)	
Outcome data	15*	*Cohort study*—Report numbers of outcome events or summary measures over time	
		Case-control study—Report numbers in each exposure category, or summary measures of exposure	
		Cross-sectional study—Report numbers of outcome events or summary measures	Page 10
Main results	16	(*a*) Give unadjusted estimates and, if applicable, confounder-adjusted estimates and their precision (eg, 95% confidence interval). Make clear which confounders were adjusted for and why they were included	
		(*b*) Report category boundaries when continuous variables were categorized	All tables
		(*c*) If relevant, consider translating estimates of relative risk into absolute risk for a meaningful time period	
Other analyses	17	Report other analyses done—eg analyses of subgroups and interactions, and sensitivity analyses	Page 12
**Discussion**			
Key results	18	Summarise key results with reference to study objectives	Page 13
Limitations	19	Discuss limitations of the study, taking into account sources of potential bias or imprecision. Discuss both direction and magnitude of any potential bias	Page 16
Interpretation	20	Give a cautious overall interpretation of results considering objectives, limitations, multiplicity of analyses, results from similar studies, and other relevant evidence	Page 17
Generalisability	21	Discuss the generalisability (external validity) of the study results	Page 17
**Other information**			
Funding	22	Give the source of funding and the role of the funders for the present study and, if applicable, for the original study on which the present article is based	Page 18

*Give information separately for cases and controls in case-control studies and, if applicable, for exposed and unexposed groups in cohort and cross-sectional studies.

Note: An Explanation and Elaboration article discusses each checklist item and gives methodological background and published examples of transparent reporting. The STROBE checklist is best used in conjunction with this article (freely available on the Web sites of PLoS Medicine at http://www.plosmedicine.org/, Annals of Internal Medicine at http://www.annals.org/, and Epidemiology at http://www.epidem.com/). Information on the STROBE Initiative is available at www.strobe-statement.org.

For peer review only
